# Electrocatalytic hydrogenation of cyanoarenes, nitroarenes, quinolines, and pyridines under mild conditions with a proton-exchange membrane reactor

**DOI:** 10.3762/bjoc.20.139

**Published:** 2024-07-11

**Authors:** Koichi Mitsudo, Atsushi Osaki, Haruka Inoue, Eisuke Sato, Naoki Shida, Mahito Atobe, Seiji Suga

**Affiliations:** 1 Division of Applied Chemistry, Graduate School of Environmental, Life, Natural Science and Technology, Okayama University, 3-1-1 Tsushima-naka, Kita-ku, Okayama 700-8530, Japan; 2 Graduate School of Engineering Science and Advanced Chemical Energy Research Center, Yokohama National University, 79-5 Tokiwadai, Hodogaya-ku, Yokohama 240-8501, Japan; 3 PRESTO, Japan Science and Technology Agency (JST), 4-1-8 Honcho, Kawaguchi, Saitama 332-0012, Japan

**Keywords:** cyanoarene, nitroarene, PEM reactor, pyridine, quinoline

## Abstract

An electrocatalytic hydrogenation of cyanoarenes, nitroarenes, quinolines, and pyridines using a proton-exchange membrane (PEM) reactor was developed. Cyanoarenes were then reduced to the corresponding benzylamines at room temperature in the presence of ethyl phosphate. The reduction of nitroarenes proceeded at room temperature, and a variety of anilines were obtained. The quinoline reduction was efficiently promoted by adding a catalytic amount of *p*-toluenesulfonic acid (PTSA) or pyridinium *p*-toluenesulfonate (PPTS). Pyridine was also reduced to piperidine in the presence of PTSA.

## Introduction

Nitrogen-containing molecules are important bioactive compounds and intermediates in chemical synthesis. Therefore, the chemical transformations of nitrogen-containing compounds have been widely studied in the field of organic synthesis [1–4]. For instance, the reduction of cyanoarenes is a straightforward and powerful method for the synthesis of primary amines [5], and the reduction of nitroarenes is useful for the synthesis of aniline derivatives [6–11]. Nitrogen-containing aliphatic heterocycles, such as piperidines and tetrahydroquinolines, are key motifs in pharmaceuticals, and the reductive syntheses of these heterocycles from pyridines and quinolines have been well studied [12]. Although these transformations have been studied intensively, such reductive reactions usually require harsh reaction conditions such as high reaction temperatures and high pressure of hydrogen [13–18].

**Figure 1:**
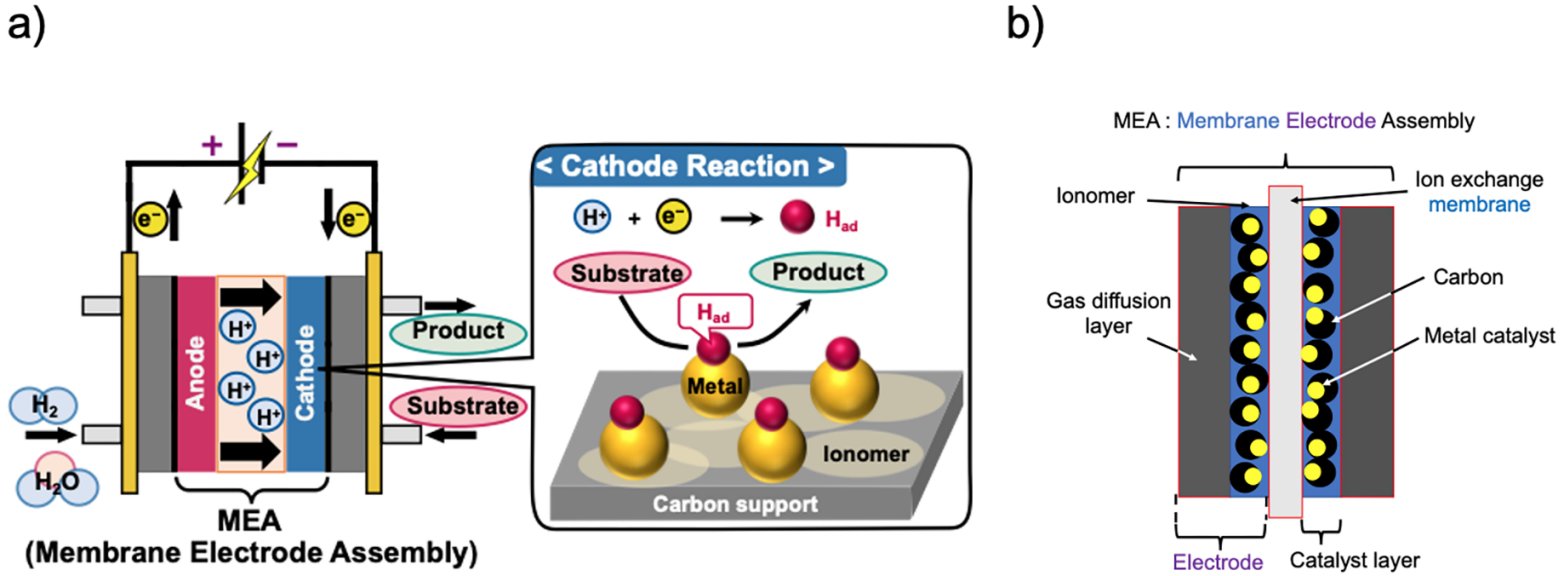
Schematic of (a) a PEM reactor and (b) MEA.

Meanwhile, electrochemical systems using solid polymer electrolytes (SPEs) have recently attracted significant attention [19]. Among these, proton-exchange membrane (PEM) reactors are powerful tools for hydrogenation [20–43]. The PEM reactor included a membrane electrode assembly (MEA) consisting of a PEM and an electro-catalyst supported on carbon (Figure 1). Humidified hydrogen gas (H_2_) or H_2_O was injected into the anodic chamber and the substrate passed through the cathodic chamber. The hydrogen (H_2_) or H_2_O were oxidized at the anode to form protons (H^+^) that moved to the cathodic chamber, and the protons were reduced to monoatomic hydrogen species (absorbed hydrogen, H_ad_). Thus-generated H_ad_ reduced the substrate passed through the cathodic chamber. MEA eliminates the need for a supporting electrolyte, which is necessary for conventional organic electrolysis, reduces the environmental impact, and facilitates product purification. In addition, using nanoparticles in the catalyst layer, which serve as the electrode, results in a large specific surface area and efficient reactions. As PEM reactors are flow reactors, they have an advantage over batch reactors in terms of continuous production.

Several reductive transformations taking advantage of the characteristics of the PEM reactor have been reported in recent years. For example, Atobe et al. reported the electrocatalytic semihydrogenation of alkynes to form *Z*-alkenes using a PEM reactor [31]. The Pd/C catalyst was essential for the reaction. They recently found that a PEM reactor with a Rh/C catalyst was effective for the stereoselective reduction of cyclic ketones [40]. Nagaki et al. reported the electrochemical deuteration of aryl halides [42]. PEM reactors are also gaining industrial attention. Weber et al. reported a series of large-scale syntheses using PEM reactors [43].

We studied electrochemical transformations [44–49] and recently reported the selective reduction of enones using a PEM reactor [50]. As mentioned above, there have been several reports on reductive reactions using PEM reactors; however, the application of PEM reactors for precise chemical transformations remains limited. To the best of our knowledge, no reports are available on the efficient reduction of cyanoarenes, quinolines, and pyridines using PEM reactors [51–52].

In this context, we have focused on the synthesis of nitrogen-containing molecules using a PEM reactor. Herein, we report the application of a PEM reactor for the reduction of cyanoarenes, nitroarenes, quinolines, and pyridines. These reductions proceeded smoothly to afford benzylamines, anilines, tetrahydroquinolines, and piperidines using a PEM reactor under ambient conditions.

## Results and Discussion

### Reduction of cyanoarenes to benzylamines

Benzonitrile (**1a**) was chosen as the model substrate, and the electroreductive hydrogenation of **1a** was performed with a PEM reactor (Table 1). Humidified hydrogen was used as a proton source, and 4.0 F mol^−1^ of electricity was passed to a circulated solution of **1a** (for the details, see the Supporting Information File 1). When Pd/C was used as the cathode catalyst, benzylamine (**2a**) was not obtained (Table 1, entry 1). Ru/C, Pt/C, and an alloy catalyst PtRu/C were also ineffective (Table 1, entries 2–4). Further screening revealed that trace amounts of **2a** were obtained when an alloy catalyst PtPd/C was used as the cathode catalyst (Table 1, entry 5). Although the desired compound **2a** was obtained using PtPd/C, undesired dibenzylamine (**3a**), was obtained as a major product [53]. To suppress the generation of **3a**, we examined the effect of solvent (Table 1, entries 6 and 7). When the reaction was performed in a mixed solvent consisting of CH_2_Cl_2_ and 1,1,1,3,3,3-hexafluoropropan-2-ol (HFIP), the yield of **2a** increased; however, the generation of **3a** was not completely suppressed (Table 1, entry 6). The use of CH_2_Cl_2_/2,2,2-trifluoroethanol (TFE) gave similar results (Table 1, entry 7).

**Table 1:** Effect of cathode catalyst for the electrochemical reduction of **1a** using a PEM reactor^a^.

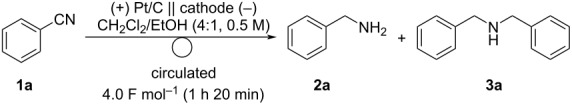				

entry	cathode catalyst	**2a** (%)^b^	**3a** (%)^b^	recovered **1a** (%)^b^

1	Pd/C	N.D.^c^	N.D.	100
2	Ru/C	N.D.	N.D.	100
3	Pt/C	N.D.	N.D.	100
4	PtRu/C	N.D.	4	85
5	PtPd/C	4	18	78
6^d^	PtPd/C	11	7	79
7^e^	PtPd/C	13	19	65

^a^Reaction conditions: anode catalyst, Pt/C; **1a**, 2.5 mmol; solvent, CH_2_Cl_2_/EtOH (4:1, 0.5 M); flow rate of the solution of **1a**, 0.25 mL min^−1^; flow rate of H_2_ gas, 100 mL min^−1^; reaction temperature, room temperature; current density, 50 mA cm^−2^. The solution was circulated until the passage of 4.0 F mol^−1^ (1 h 20 min). ^b^Area ratio determined by gas chromatography analysis. ^c^Not detected. ^d^Performed using HFIP instead of EtOH. ^e^Performed using TFE instead of EtOH.

**Scheme 1:**
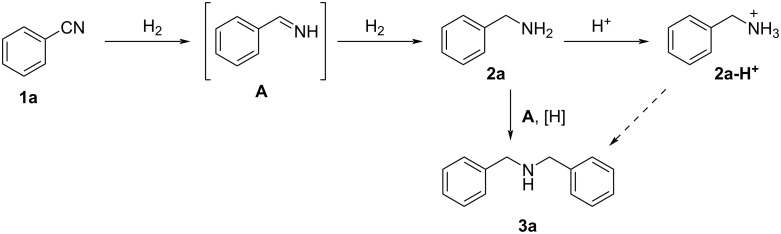
Plausible mechanism for the reduction of **1a** leading to benzylamine **2a** and dibenzylamine **3a**.

A plausible mechanism for the reduction of **1a** is shown in Scheme 1. First, the reduction of **1a** afforded phenylmethanimine (**A**). Further reduction of **A** afforded the desired benzylamine (**2a**). However, nucleophilic attack of **2a** on **A**, followed in situ by reduction, proceeded competitively to form dibenzylamine (**3a**). We considered that by protonating **2a** to form **2a-H^+^**, its nucleophilic nature could be suppressed, thereby inhibiting the formation of **3a**.

Based on this hypothesis, we examined the reduction of **1a** in the presence of several acids (Table 2). First, electrochemical reduction was performed with 0.2 or 1.0 equiv of acetic acid, but the yield of **3a** did not decrease, suggesting that **2a** could not be trapped by acetic acid (p*K*_a_ = 4.75). To ensure the capture of **2a**, we performed reduction with phosphoric acid (p*K*_a_ = 2.12). Although the generation of **3a** was suppressed, only a trace amount of **2a** was obtained, and almost **1a** was recovered (Table 2, entry 4). This was probably because the presence of water inhibited the reaction. Therefore, it was necessary to perform the reaction under anhydrous conditions. Hence, we used ethyl phosphate (mono- and di-mixture) (p*K*_a_ = 1.42), which reacts easily under anhydrous conditions. As expected, the generation of **3a** was suppressed and **2a** was selectively obtained (Table 2, entry 5). With the increase of the electricity to 16.0 F mol^−1^, **2a** was obtained selectively in 88% yield (Table 2, entry 6).

**Table 2:** Electrochemical reduction of **1a** in the presence of acids^a^.

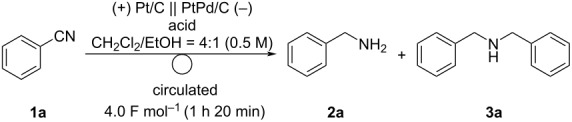				

entry	acid (equiv)	**2a** (%)^b^	**3a** (%)^b^	recovered **1a** (%)^b^

1	none	4	18	78
2	CH_3_COOH (0.2)	2	15	82
3	CH_3_COOH (1.0)	3	22	74
4	H_3_PO_4_ (1.0)	1	N.D.^c^	99
5	ethyl phosphate^d^ (1.0)	18	trace	82
6^e^	ethyl phosphate^d^ (1.0)	88^f^	trace^f^	7^f^

^a^Reaction conditions: anode catalyst, Pt/C; cathode catalyst, PtPd/C; **1a**, 2.5 mmol; solvent, CH_2_Cl_2_/EtOH (4:1, 0.5 M); flow rate of the solution of **1a**, 0.25 mL min^−1^; flow rate of H_2_ gas, 100 mL min^−1^; reaction temperature, room temperature; current density, 50 mA cm^−2^. The solution was circulated until the passage of 4.0 F mol^−1^ (1 h 20 min). ^b^Area ratio determined by gas chromatography analysis. ^c^Not detected. ^d^Mono- and di- ester mixture. ^e^16.0 F mol^−1^ (5 h 20 min). ^f^GC yield determined by GC analysis using dodecane as an internal standard.

**Scheme 2:**
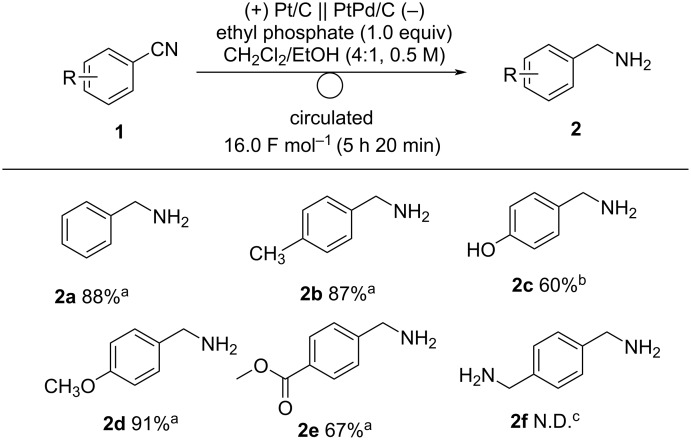
Electrochemical reduction of cyanoarenes under optimal conditions. Reaction conditions: anode catalyst, Pt/C; cathode catalyst, PtPd/C; **1**, 2.5 mmol; solvent, CH_2_Cl_2_/EtOH (4:1); flow rate of the solution of **1**, 0.25 mL min^−1^; flow rate of H_2_ gas, 100 mL min^−1^; reaction temperature, room temperature; current density, 50 mA cm^−2^. The solution was circulated until the passage of 16.0 F mol^−1^ (5 h 20 min). ^a^Determined by GC analysis using *n*-dodecane as an internal standard. ^b^Determined by ^1^H NMR analysis using 1,1,2,2-tetrachloroethane as an internal standard. ^c^Not detected.

Next, we examined the scope of the electrochemical reduction of cyanoarenes under optimal conditions (Scheme 2). The reactions of cyanoarenes bearing electron-donating methyl, hydroxy, and methoxy groups proceeded smoothly to afford the corresponding benzylamines **2b–d** in moderate-to-good yields. Electron-withdrawing groups, such as esters, can be tolerated under these conditions. Unfortunately, the electrochemical reduction of 1,4-dicyanobenzene (**1f**) did not give the desired product **2f** probably due to the oligomerization of the substrate.

### Reduction of nitroarenes to anilines

Next, we reduced nitroarenes using a PEM reactor. First, the electrocatalyst and solvent were optimized (Table 3). While 6.0 F mol^−1^ of electricity should be required for the reduction of nitrobenzene (**4a**) to aniline (**5a**), the charge for screening was set to 2.0 F mol^−1^ for rapid evaluation, and several cathode catalysts were examined. When Pd/C was used as the cathode, the reaction did not proceed (Table 3, entry 1). The desired reduction proceeded with Ru/C and **5a** was obtained in 79% of current efficiency (Table 3, entry 2). Pt/C afforded the best result (90% current efficiency, Table 3, entry 3). To increase the yield, the reaction was carried out until **4a** was consumed. After 7 h of electrolysis (23.2 F mol^−1^), **4a** was completely consumed and **5a** was obtained in 82% yield. Although Ir/C was inefficient (Table 3, entry 4), Rh/C was as efficient as Pt/C (Table 3, entry 5). As Rh is more expensive than Pt, Pt/C was selected as the best cathode catalyst.

**Table 3:** Electrochemical reduction of **4a** with several cathode catalysts^a^.

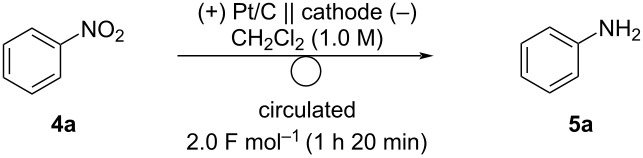				

entry	cathode catalyst	**5a** (%)^b^	current efficiency (%)^b^	recovered **4a** (%)^b^

1	Pd/C	N.D.^c^	–	–
2	Ru/C	25	79	65
3	Pt/C	30 (82)^d^	90 (20)^d^	76 (N.D.)^d^
4	Ir/C	6	18	70
5	Rh/C	29	90	59

^a^Reaction conditions: anode catalyst, Pt/C; **4a**, 5 mmol; solvent, CH_2_Cl_2_ (1.0 M); flow rate of the solution of **4a**, 0.25 mL min^−1^; flow rate of H_2_ gas, 100 mL min^−1^; reaction temperature, room temperature; current density, 50 mA cm^−2^. The solution was circulated until the passage of 2.0 F mol^−1^ (1 h 20 min). ^b^Determined by GC using *n*-dodecane as an internal standard. ^c^Not detected. ^d^Performed until **4a** was consumed using 2.25 mmol of **4a** (7 h, 23.2 F mol^−1^).

Although **5a** was obtained in good yield with Pt/C, the current efficiency was low. We assumed that this was due to the recombination of H_ad_ to form hydrogen. To suppress hydrogen generation, we varied the flow rate of the reaction solution (Table 4). The reaction time was set to 2.5 h (2.25 mmol, 8.3 F mol^–1^) and the flow rate of the reaction solution was changed from 0.25 to 1.0 mL min^−1^. As expected, increasing the flow rate increased the current efficiency and yield of **5a**. With 0.75 mL min^−1^ of flow rate, **5a** was obtained in 88% yield with 62% of current efficiency (Table 4, entry 3). In contrast to the previous report on electrocatalytic hydrogenation of nitrobenzene using a PEM reactor [21], cyclohexylamine was not observed in each reaction.

**Table 4:** Effect of flow rate in the electrochemical reduction of **4a**^a^.

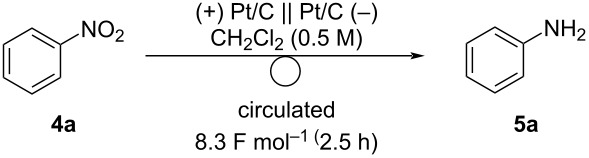				

entry	flow rate (mL min^-1^)	**5a** (%)^b^	current efficiency (%)^b^	recovered **4a** (%)^b^

1	0.25	75	53	14
2	0.50	78	56	7
3	0.75	88	62	3
4	1.0	88	63	2

^a^Reaction conditions: anode catalyst, Pt/C; cathode catalyst, Pt/C; **4a**, 2.25 mmol; solvent, CH_2_Cl_2_ (0.5 M); flow rate of the solution of **4a**, 0.25–1.0 mL min^−1^; flow rate of H_2_ gas, 100 mL min^−1^; reaction temperature, room temperature; current density, 50 mA cm^−2^. The solution was circulated for 2.5 h (8.3 F mol^−1^). ^b^Determined by GC analysis using *n*-dodecane as an internal standard.

**Scheme 3:**
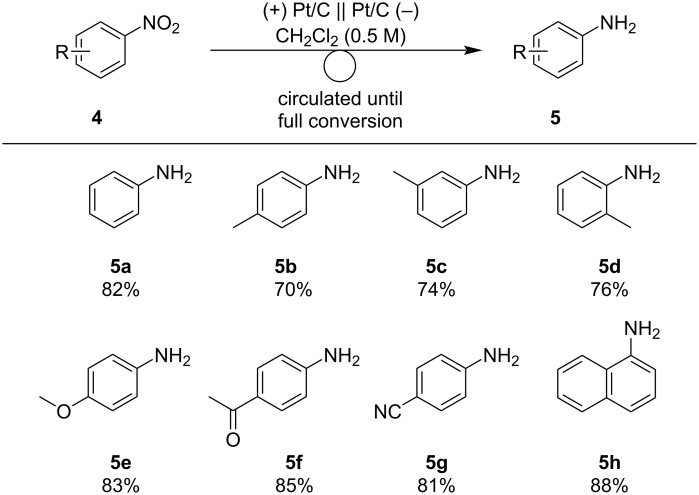
Scope of the electrochemical reduction of nitroarenes. Reaction conditions: anode catalyst, Pt/C; cathode catalyst, Pt/C; **4**, 2.25 mmol; solvent, CH_2_Cl_2_ (0.5 M); flow rate of the solution of **4**, 0.75 mL min^−1^; flow rate of H_2_ gas, 100 mL min^−1^; reaction temperature, room temperature; current density, 50 mA cm^−2^. The solution was circulated until the full conversion of **4**. Isolated yield.

Next, the scope of the nitroarene electro-reduction was explored (Scheme 3). To obtain products in high yields, the electrolysis was performed until the substrates were consumed. First, nitroarenes bearing electron-donating groups were investigated. Nitroarenes **4b**–**d** bearing methyl groups gave the corresponding anilines **5b–d** in 70–76% yield. *p*-Methoxyaniline (**5e**) was obtained in 83% yield. Nitroarenes bearing electron-withdrawing groups are also useful. Acetyl and cyano groups were tolerated under the reaction conditions and anilines **5f** and **5g** were obtained in 85% and 81% yields, respectively. 1-Naphthylamine, a more π-extended aniline was easily obtained in a high yield.

### Reduction of quinolines to tetrahydroquinolines

**Figure 2:**
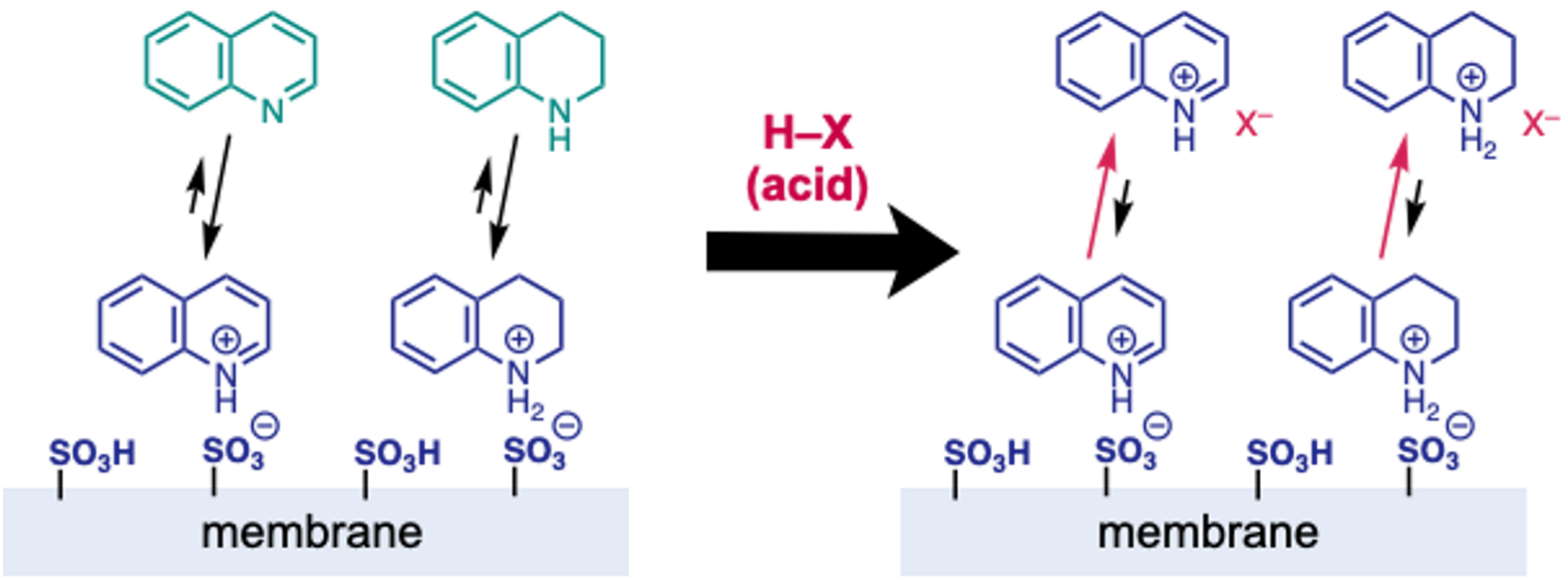
Hypothesis of the trap of quinoline on membrane and tetrahydroquinoline and the effect of adding an acid.

The electrochemical reduction of quinolines was performed using a PEM reactor. First, several different cathode catalyst were examined for the reduction of quinoline (**6a**) (Table 5). Because 4.0 F mol^−1^ of electricity should be required ideally to reduce quinoline (**6a**) to 1,2,3,4-tetrahydroquinoline (**7a**), 4.0 F mol^−1^ of electricity was applied for the reactions. Pd/C, Ir/C, Ru/C, and Pt/C were used as cathode catalysts, and 3–5% yields of **7a** were obtained by the use of each catalyst (Table 5, entries 1–4). We chose Pt/C, one of the most common catalysts used in fuel-cell reactors, and increased the charge to complete the reaction. With 50 F mol^−1^ of electricity, **6a** was completely consumed and **7a** was obtained in 96% yield (Table 5, entry 5). However, the electrochemical reaction of **6a** with reused MEA did not give **7a**, and MEA was torn after electrolysis, probably because **6a** and/or **7a** were trapped on the membrane having sulfonic acids, and the desired electrolysis was disturbed. We assumed that this could be resolved by adding a strong acid to liberate **6a** and/or **7a** from the membrane by the equilibrium (Figure 2).

**Table 5:** Electrochemical reduction of **6a** with several cathode catalysts^a^.

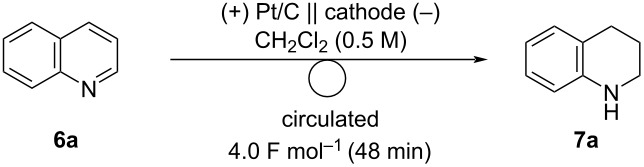			

entry	cathode catalyst	**7a** (%)^b^	recovered **6a** (%)^b^

1	Pd/C	5	86
2	Ir/C	3	86
3	Ru/C	4	70
4	Pt/C	3	86
5^c^	Pt/C	96	N.D.^d^
6^e^	Pt/C	N.D.	N.D.

^a^Reaction conditions: anode catalyst, Pt/C; **6a**, 1.5 mmol; solvent, CH_2_Cl_2_ (0.5 M); flow rate of the solution of **6a**, 0.75 mL min^−1^; flow rate of H_2_ gas, 100 mL min^−1^; reaction temperature, room temperature; current density, 50 mA cm^−2^. The solution was circulated until the passage of 4.0 F mol^−1^ (48 min). ^b^Determined by ^1^H NMR spectroscopy using 1,1,2,2-tetrachloroethane as an internal standard. ^c^50 F mol^−1^ (1st run). ^d^Not detected. ^e^50 F mol^−1^ (2nd run).

**Figure 3:**
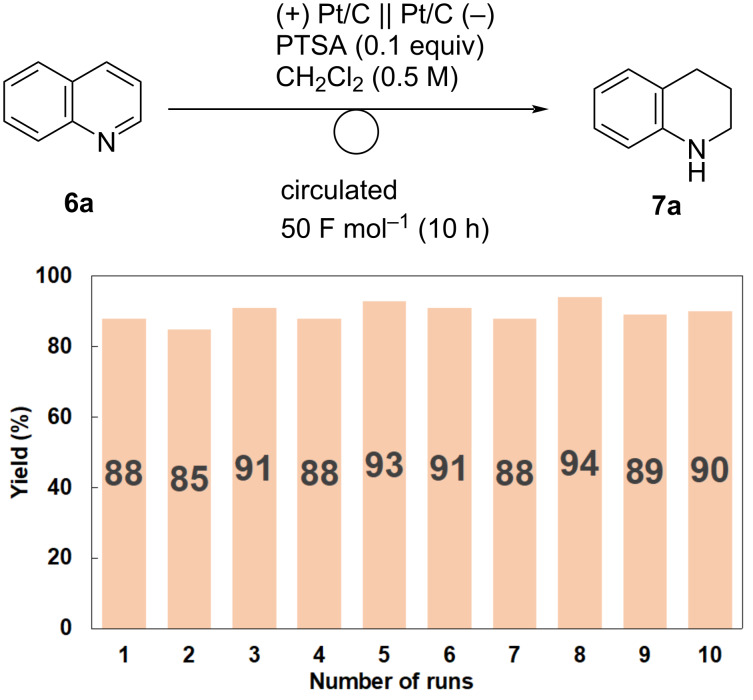
Recycled use of MEA for the electroreduction of **6a** in the presence of PTSA (0.10 equiv). Reaction conditions: anode catalyst, Pt/C; cathode catalyst, Pt/C; **6a**, 1.5 mmol; solvent, CH_2_Cl_2_ (0.5 M); flow rate of the solution of **6a**, 0.75 mL min^−1^; flow rate of H_2_ gas, 100 mL min^−1^; reaction temperature, room temperature; current density, 50 mA cm^−2^. The solution was circulated until the passage of 50 F mol^−1^ (10 h). The yields were determined by ^1^H NMR analysis using 1,1,2,2-tetrachloroethane as an internal standard.

**Figure 4:**
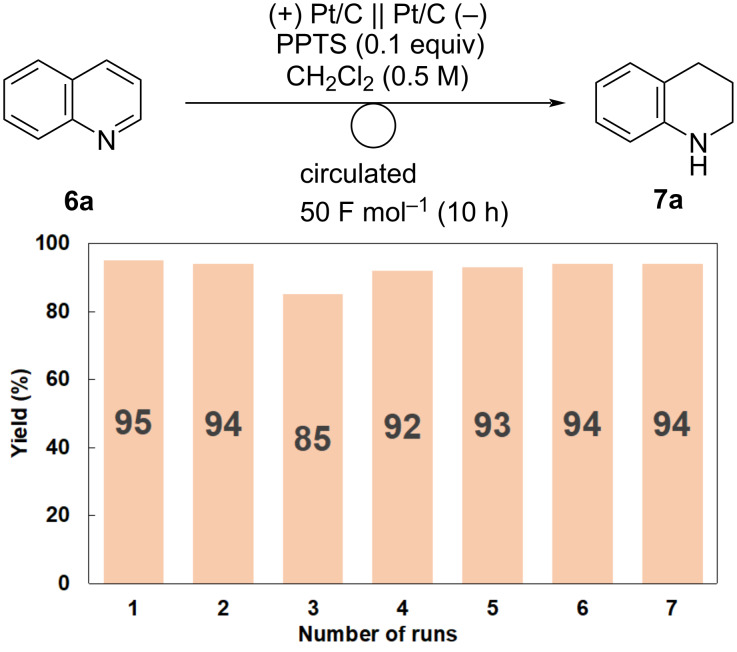
Recycled use of MEA for the electroreduction of **6a** in the presence of PPTS (0.10 equiv). Reaction conditions: anode catalyst, Pt/C; cathode catalyst, Pt/C; **6a**, 1.5 mmol; solvent, CH_2_Cl_2_ (0.5 M); flow rate of the solution of **6a**, 0.75 mL min^−1^; flow rate of H_2_ gas, 100 mL min^−1^; reaction temperature, room temperature; current density, 50 mA cm^−2^ (10 h). The solution was circulated until the passage of 50 F mol^−1^. The yields were determined by ^1^H NMR analysis using 1,1,2,2-tetrachloroethane as an internal standard.

Based on this hypothesis, we examined several acids and found that the addition of a catalytic amount (0.10 equiv) of *p*-toluenesulfonic acid (PTSA) was sufficient (Figure 3). The first run gave **7a** in 88% yield and the second run with the MEA gave **7a** in 85% yield. MEA was reused eight times, and **7a** was obtained in high yield in each run. The addition of pyridinium *p*-toluenesulfonate (PPTS) was also efficient, and MEA was repeatedly used to afford **7a** in high yield (Figure 4).

**Scheme 4:**
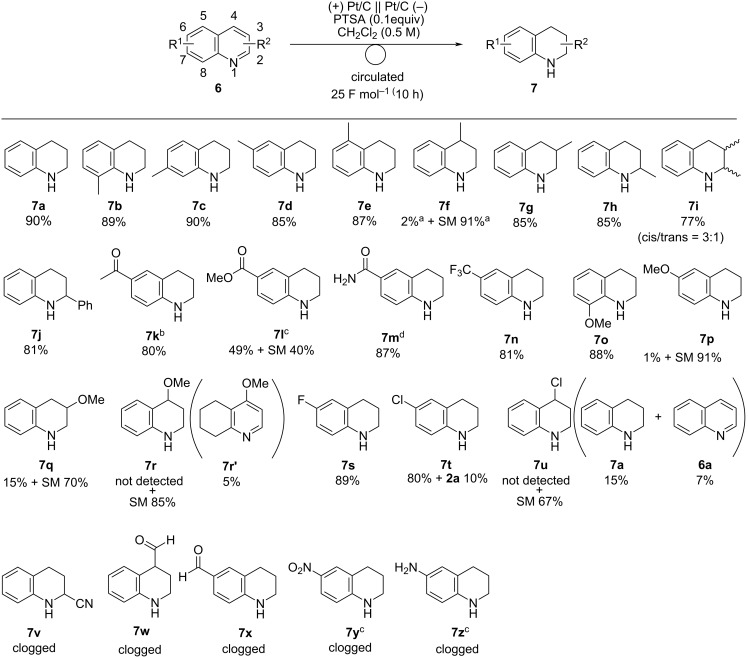
Scope of the electroreduction of **6** in the presence of PTSA (0.10 equiv). Reaction conditions: anode catalyst, Pt/C; cathode catalyst, Pt/C; **6**, 1.5 mmol; solvent, CH_2_Cl_2_ (0.5 M); flow rate of the solution of **6**, 0.75 mL min^−1^; flow rate of H_2_ gas, 100 mL min^−1^; reaction temperature, room temperature; current density, 25 mA cm^−2^. The solution was circulated until the passage of 25 F mol^−1^ (10 h). Isolated yield. ^a^Determined by ^1^H NMR analysis using 1,1,2,2-tetrachloroethane as an internal standard. ^b^0.125 M. ^c^0.25 M. ^d^1,4-dioxane/H_2_O (7:1, 0.25 M).

After further tunings, we found that the charge for electro-reduction of **6a** could be reduced to 25 F mol^−1^ when decreasing the current density to 25 mA cm^−2^, and **7a** was obtained in 90% yield (see the Supporting Information File 1). Next, the substrate scope was examined under optimal conditions (Scheme 4). Substrates bearing a methyl group afforded the corresponding products in high yields (**7a–e**, **7g**, and **7h**), except for **6f** which contained a methyl group at the 4-position. The reaction of 2,3-dimethylquinoline (**6i**) gave the desired product **7i** (*cis*/*trans* = 3:1) in 77% yield. Substrates bearing acetyl (**6k**), ester (**6l**), and amide (**6m**) groups were tolerated under these conditions and selectively afforded the desired products. A substrate with a methoxy group at the 8-position (**6o**) afforded the desired product **7o** in high yield. However, **6p** and **6q** with a methoxy group at the 6- and 3-position gave only a small amount of the target product, and **6r** with a methoxy group at the 4-position gave compound **7r′**, in which the benzene ring was hydrogenated. Substrates with chloro groups produced the dechlorinated products (**7t** and **7u**). Unfortunately, during the electrolysis of quinolines with cyano (**6v**), formyl (**6w** and **6x**), nitro (**6y**), and amino (**6z**) groups, the flow path was clogged probably due to the decomposition of the substrates, and the desired products were not obtained.

**Scheme 5:**
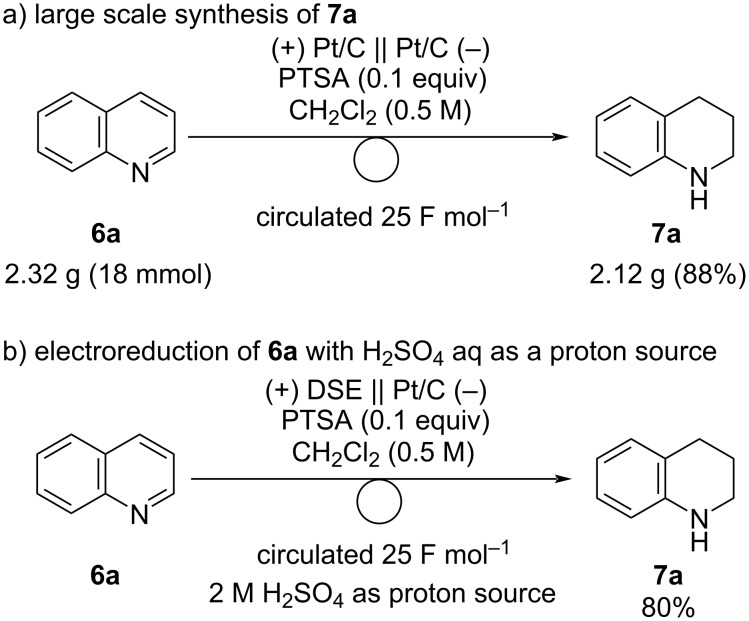
a) Large scale synthesis of **7a** and b) electoreduction of **6a** using H_2_SO_4_ as a proton source.

This system can be applied to large-scale syntheses. A similar yield of **7a** was obtained when the reaction was scaled up (Scheme 5a). The electrolysis of 2.32 g of **6a** gave 2.12 g of **7a** (88% yield). Next, we examined the electroreduction of **6a** using an aqueous proton source instead of hydrogen. The use of DSE^®^ as an anode and H_2_SO_4_ aq as an anolyte was effective, and **7a** was obtained in 80% yield (Scheme 5b).

### Reduction of pyridines to piperidines

As mentioned previously, reduction of pyridines to piperidines is important for organic synthesis. Therefore, the electroreduction of pyridine (**8a**) was performed (Table 6). First, the electroreduction of **8a** was performed with 0.1 equiv of PTSA, and 1.0 equiv of PTSA was added after electrolysis to determine the yield by ^1^H NMR analysis (Table 6, entry 1). The yield of **9a**·PTSA (26% yield) was low and **8a**·PTSA was obtained as the major product (60% yield), suggesting that the catalytic amount of PTSA was not sufficient because it would be completely trapped with **9a**. These results suggest that stoichiometric amount of PTSA was required to liberate **8a** from the membrane. As expected, the yield of **9a**·PTSA increased upon increasing the amount of PTSA used for electrolysis (Table 6, entries 1–4). Electrolysis with 1 equiv of PTSA afforded **9a**·PTSA quantitatively (Table 6, entry 4). MEA was used repeatedly, and the target compound was obtained quantitatively in each run (Table 6, entries 5 and 6). Finally, the reaction was examined using an aqueous proton source instead of humidified H_2_ gas. Similar to the reaction of quinoline (**6a**), **9a**·PTSA was obtained in 85% yield (Table 6, entry 7) by the use of DSE^®^ electrode and 2 M H_2_SO_4_ aq as a proton source.

**Table 6:** Electroreduction of pyridine (**8a**) using a PEM reactor^a^.

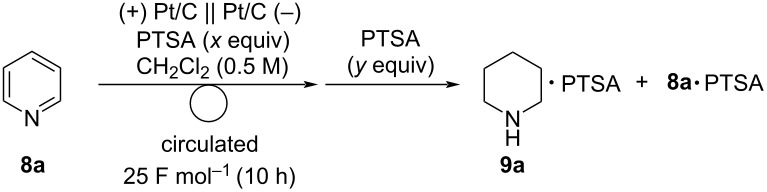				

entry	*x* (equiv)	*y* (equiv)	**9a**·PTSA (%)^b^	**8a**·PTSA (%)^b^

1	0.1	1	26	60
2	0.5	0.5	48	47
3	0.75	0.25	77	23
4	1	0	quant	N.D.^c^
5^d^	1	0	99	N.D.
6^e^	1	0	quant	N.D.
7^f^	1	0	85	N.D.

^a^Reaction conditions: anode catalyst, Pt/C; cathode catalyst, Pt/C; **8a**, 1.5 mmol; solvent, CH_2_Cl_2_ (0.5 M); flow rate of the solution of **8a**, 0.75 mL min^−1^; flow rate of H_2_ gas, 100 mL min^−1^; reaction temperature, room temperature; current density, 25 mA cm^−2^. The solution was circulated until the passage of 25 F mol^−1^ (10 h). ^b^Determined by ^1^H NMR analysis using 1,1,2,2-tetrachloroethane as an internal standard. ^c^Not detected. ^d^2nd run. ^e^3rd run. ^f^Performed with DSE^®^ electrode and 2 M H_2_SO_4_ aq was used instead of humidified H_2_ gas.

**Scheme 6:**
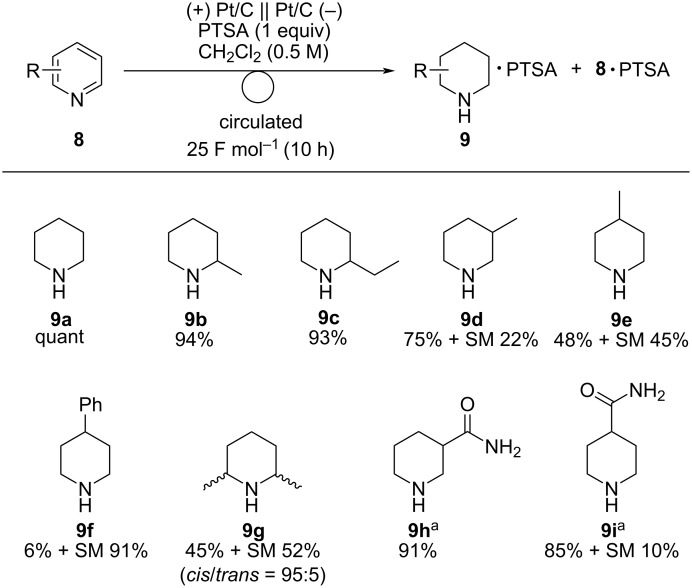
Scope of the electroreduction of **6** in the presence of PTSA (1 equiv). Reaction conditions: anode catalyst, Pt/C; cathode catalyst, Pt/C; **8**, 1.5 mmol; solvent, CH_2_Cl_2_ (0.5 M); flow rate of the solution of **8**, 0.75 mL min^−1^; flow rate of H_2_ gas, 100 mL min^−1^; reaction temperature, room temperature; current density, 25 mA cm^−2^. The solution was circulated until the passage of 25 F mol^−1^ (10 h). Yields were determined by ^1^H NMR analysis of PTSA salts using 1,1,2,2-tetrachloroethane as an internal standard. ^a^Performed in 1,4-dioxane/H_2_O (7:1), and the yield was determined by ^1^H NMR analysis using ethylene carbonate as an internal standard.

The substrate scope was investigated under the optimized reaction conditions (Scheme 6). Electroreduction of 2-methylpyridine (**8b**) and 2-ethylpyridine (**8c**) afforded the corresponding 2-substituted piperidines **9b** and **9c** in 94% and 93% yields, respectively. The reaction of 3-methylpyridine (**8d**) gave 3-methylpiperidine (**9d**) in good yield. In contrast to quinolines, 4-methylpyridine (**8e**) gave 4-methylpiperidine (**9e**) in a moderate yield. 4-Phenylpyridine (**8f**) afforded a small amount of the target product **9f** and 91% of **8f** was recovered, probably because of steric hindrance of **8f**. 2,6-Disubstituted pyridine such as 2,6-lutidine (**8g**) was also applicable and 2,6-dimethylpiperidine (**9g**) was obtained in moderate yield. In contrast to quinolines, pyridines bearing an amide group were also applicable and amidylpiperidines **9h** and **9i** were obtained in high yields.

## Conclusion

We established the electrochemical reduction of cyanoarenes, nitroarenes, quinolines, and pyridines using a PEM reactor. All the reactions proceeded under ambient conditions, and benzylamines, anilines, 1,2,3,4-tetrahydroquinolines, and piperidines were obtained. For the electrochemical reduction using a PEM reactor, the addition of an acid sometimes helped the progress of the reactions. For instance, the addition of ethyl phosphate is essential for the electroreduction of cyanoarenes. The generation of dibenzylamine was suppressed and benzylamines were obtained efficiently. The PEM system was effective in reducing nitroarenes. Several functional groups were tolerated under these conditions, and the nitro group was selectively reduced. The addition of an acid was also effective in reducing quinolines to 1,2,3,4-tetrahydroquinolines. In the presence a catalytic amount of PTSA, various 1,2,3,4-tetrahydroquinolines were obtained. Although a stoichiometric amount of PTSA was required, this system was applicable to the reduction of pyridines to quinolines. An aqueous proton source could also be used in this system. The fact that the addition of appropriate strength and amount of acid makes the reaction system more efficient is a key factor in the reduction of nitrogen-containing compounds with the PEM-type reactor. The chemoselective reduction of nitrogen-containing compounds under mild conditions is important for organic synthesis, and we believe that the PEM reaction system is a powerful tool that can be applied to a wide variety of nitrogen-containing compounds.

## Supporting Information

File 1 Experimental part.

## Data Availability

All data that supports the findings of this study is available in the published article and/or the supporting information to this article.
