# Supplementary material for: Electrocatalytic hydrogenation of cyanoarenes, nitroarenes, quinolines, and pyridines under mild conditions with a proton-exchange membrane reactor
Source: Beilstein J Org Chem. 2024 Jul 11;20:1560–71. doi: 10.3762/bjoc.20.139 (PMC11250234; doi:10.3762/bjoc.20.139)

BEILSTEIN JOURNAL OF ORGANIC CHEMISTRY

Supporting Information

for

Electrocatalytic hydrogenation of cyanoarenes, nitroarenes, quinolines, and pyridines under mild conditions with a proton-exchange membrane reactor

Koichi Mitsudo, Atsushi Osaki, Haruka Inoue, Eisuke Sato, Naoki Shida, Mahito Atobe and Seiji Suga

Beilstein J. Org. Chem. **2024,** *20,* 1560–1571. doi:10.3762/bjoc.20.139

Experimental part

License and Terms: This is a supporting information file under the terms of the Creative Commons Attribution License (https://creativecommons.org/licenses/by/4.0). Please note that the reuse, redistribution and reproduction in particular requires that the author(s) and source are credited and that individual graphics may be subject to special legal provisions.
The license is subject to the *Beilstein Journal of Organic Chemistry* terms and conditions: (https://www.beilstein-journals.org/bjoc/terms)

General

Gas chromatography (GC) analyses were performed by using a SHIMADZU gas chromatograph (GC2014s with a capillary column thickness of film: 0.25 μm, length: 25.0 m, inner diameter: 0.22 mm (CBP1-M25-025, SHIMADZU GLC Ltd.), carrier gas: helium). Constant-current electrolysis was carried out with a DC power supply (KIKUSUI PMX350-0.2A). Nuclear magnetic resonance (NMR) spectra were recorded on JEOL JNM-ECZ600R (^{1}H 600 MHz, ^{13}C 150 MHz, ^{19}F 565 MHz), JEOL JNM-ECS400 (^{1}H 400 MHz, ^{13}C 100 MHz, ^{19}F 376 MHz), Varian 400-MR ASW (^{1}H 400 MHz, ^{13}C 100 MHz) and Varian NMR System PS600 (^{1}H 600 MHz, ^{13}C 150 MHz) spectrometers. Chemical shifts for ^{1}H NMR are expressed in parts per million (ppm) relative to TMS (δ 0.00 ppm) or residual $CHCl_3$ in $CDCl_3$ (δ 7.26 ppm) or H_2O in D_2O (δ 4.79 ppm). Chemical shifts for ^{13}C NMR are expressed in parts per million (ppm) relative to TMS (δ 0.00 ppm) or $CDCl_3$ (δ 77.16 ppm) with a complete proton decoupling. Chemical shifts for ^{19}F NMR are expressed in ppm relative to α,α,α-trifluorotoluene (δ −63.9 ppm) or hexafluorobenzene (δ −162.9 ppm). IR spectra were recorded on a SHIMADZU IRAffinity-1 spectrophotometer. High-resolution mass spectrometry was performed on a Bruker micrOTOF II-SKA (ESI-MS). Analytic thin layer chromatography (TLC) was performed on Merck, pre-coated plate silica gel 60 F_{254} (0.25 mm thickness). Column chromatography was performed on KANTO CHEMICAL silica gel 60N (40–50 μm).

Materials

All chemicals were purchased from commercial suppliers. All chemicals were used without further purification. Stainless steel end plates and carbon separators for a PEM reactor were purchased from CHEMIX Co., Ltd. As for manufacturing the membrane electrode assembly (MEA), Nafion® perfluorinated membrane (Nafion® NR212) was purchased from Furukawa agency Co., Ltd. Nafion® perfluorinated resin solution (5 wt % in mixture of lower aliphatic and water, Nafion® DE521) as ionomer solution was purchased from Sigma-Aldrich Co. or Wako Pure Chemical Industries. Fuel cell catalysts (TEC10E50E; Pt/C, TECRU(ONLY)E30; Ru/C, TEC66E50; Pt50Ru50/C and TEC62E58; Pt33Ru67/C) were purchased from Tanaka Kikinzoku Kogyo K.K. (TKK). Pd/C was purchased from ISHIFUKU Metal Industry Co., Ltd. PtPd/C, and Ir/C were supplied by ISHIFUKU Metal Industry Co., Ltd. DSE® electrode was supplied by De Nora Permelec Ltd. Gas diffusion layer (GDL39BB) was purchased from SGL CARBON GmbH. 2,3-Dimethylquinoline (**6i**)[1] and 6-(trifluoromethyl)quinoline (**6t**)[2] were synthesized according to the literature.

Preparation of MEAs

Fuel cell catalyst (244 mg), deionized water (1.2 g), Nafion® perfluorinated resin solution, and 1-propanol (1.4 g) were stirred in a glass vial. This mixture and zirconia balls (diameter: 3.0 mm, 10 pieces) were added to a Teflon® vessel. The vessel was placed in a planetary rotation pot mill (PULVERISETTE, Fritsch Japan Co. Ltd.) and rotated at 200 rpm for 20 min. After the rotation, catalyst dispersion was obtained. Gas diffusion electrodes (GDE, 5 cm × 5 cm) were homemade by spraying catalyst inks (Nafion®/catalysts weight ratio of 0.8:1, 1-propanol as dispersant) on commercialized GDL39BB to form a catalyst layer and then drying at 120 °C, 3 min. After spraying, the GDE sheet was cut into 1 cm × 4 cm pieces. Two sheets of this catalyst layer were used for the anode and the cathode. Nafion® perfluorinated membrane (3 cm × 10 cm) was put between the anode and the cathode, and their catalyst sides faced to the membrane. Finally, this was hot-pressed (0.4 MPa, 150 °C) for 10 min. Pt/C was used as an anode

catalyst material and Pt/C, Pd/C, Ir/C, Ru/C, PtPd/C, and PtRu/C were used as cathode catalyst materials. The loading amount of each catalyst was 0.5 mg/cm^2.

Preparation of a PEM reactor

As shown in Figure S1, stainless steel, carbon separators, Teflon® gaskets, and MEA were used to fabricate the PEM reactor, which was tightened to 2.0 Nm with M6 size screws using a torque wrench.

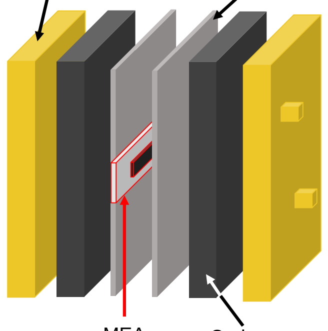

Figure S1. PEM reactor

General procedure for hydrogenation of cyanoarenes and nitroarenes using a PEM reactor

Electrocatalytic hydrogenations were carried out by using a PEM reactor (Figure S2). The MEA (3 cm × 10 cm, 1 cm × 4 cm: active area) was integrated into the PEM reactor. For the cathodic chamber, humidified nitrogen gas was supplied for 1 hour. After that, the solution of substrate solution was provided by a circular pump (YAMAZEN, MSP-001-0). For the anodic chamber, humidified hydrogen gas (flow rate: 100 mL/min) was supplied. A constant current was supplied at room temperature. The yields of products were determined by GC or NMR analyses.

General procedure for hydrogenation of quinoline (6) using a PEM reactor

Electrocatalytic hydrogenation was carried out by using a PEM reactor. The MEA (3 cm × 10 cm, 1 cm × 4 cm: active area) was integrated into the PEM reactor. For the cathodic chamber, humidified nitrogen gas was supplied for 1 hour. After that, the solution of the substrate (1.5 mmol) in CH_2Cl_2 (3 mL) was provided with 0.75 mL/min by a circular pump (YAMAZEN, MSP-001-0). For the anodic chamber, humidified hydrogen gas (flow rate: 100 mL/min) was supplied. A constant current (25 mA/cm^2, 25 F/mol, 10 h) was supplied at room temperature. To the obtained cathodic reaction solution was added NaOH aq (1.0 M, 5 mL), and the mixture was extracted with CH_2Cl_2 (3 × 10 mL). The combined organic phase was washed with brine, dried over Na_2SO_4, filtered, and concentrated under reduced pressure. The residue was purified by column chromatography on silica gel eluting with hexane/EtOAc to afford 1,2,3,4-tetrahydroquinoline.

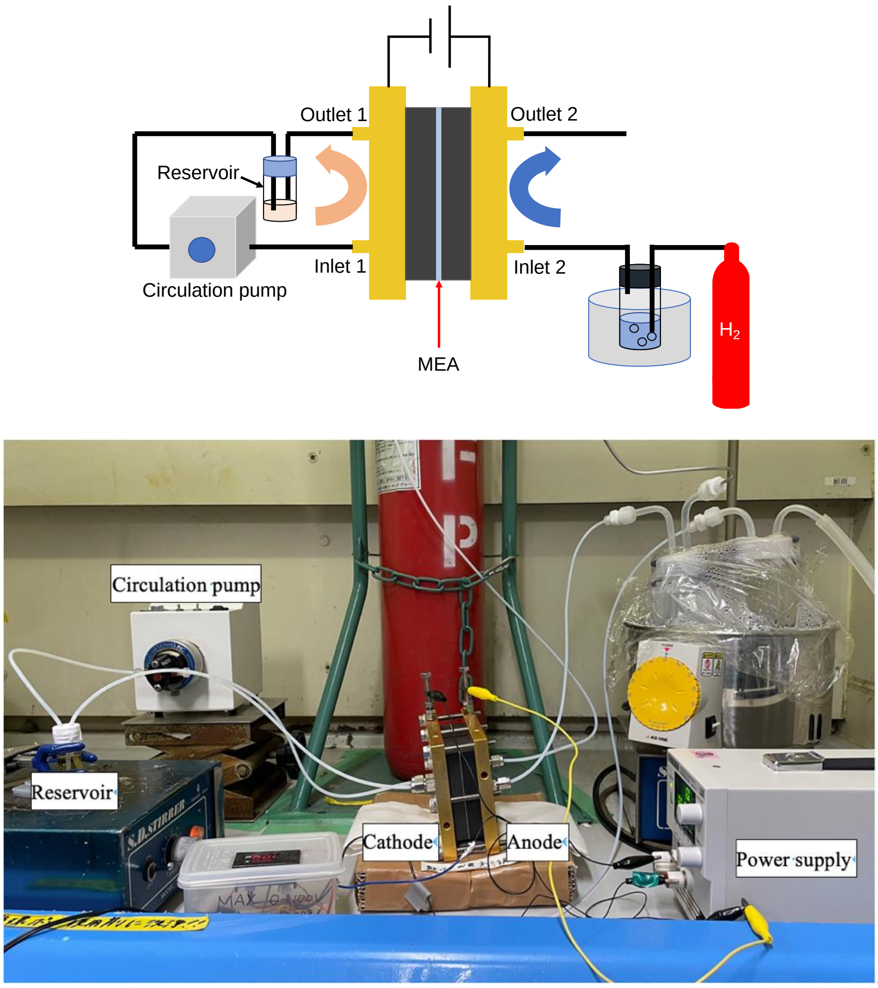

Figure S2. Hydrogenation system using a PEM reactor

1,2,3,4-Tetrahydroquinoline (7a)

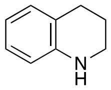

Prepared according to the general procedure from quinoline (**6a**, 194 mg, 1.50 mmol) and *p*-toluenesulfonic acid monohydrate (29 mg, 0.15 mmol) in CH_2Cl_2 (3 mL). A constant current (25 mA/cm^2, 25 F/mol, 10 h) was supplied at room temperature. After general work-up, the residue was purified by column chromatography on silica gel (hexane/EtOAc 2:1) to afford the title compound as yellow liquid (180 mg, 1.35 mmol, 90%). ^{1}H NMR (600 MHz, $CDCl_3$) δ 6.95 (td, *J* = 7.2, 1.0 Hz, 1H), 6.93 (d, *J* = 7.2 Hz, 1H), 6.59 (td, *J* = 7.2 Hz, 1H), 3.80 (brs, 1H), 3.29 (t, *J* = 6.5 Hz, 2H), 2.75 (t, *J* = 6.5 Hz, 2H), 1.93 (quint, *J* = 6.5 Hz, 2H); ^{13}C{^{1}H} NMR (150 MHz, $CDCl_3$) δ 144.9, 129.6, 126.8, 121.6, 117.1, 114.3, 42.1, 27.1, 22.3; IR (neat) 3408, 2928, 2839, 2361, 1607, 1504, 1312, 1096, 1007, 746 cm^{-1}. The spectral data were in good agreement with literature.[3]

8-Methyl-1,2,3,4-tetrahydroquinoline (7b)

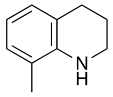

Prepared according to the general procedure from 8-methylquinoline (**6b**, 215 mg, 1.50 mmol) and *p*-toluenesulfonic acid monohydrate (28 mg, 0.15 mmol) in CH_2Cl_2 (3 mL). A constant current (25 mA/cm^2, 25 F/mol, 10 h) was supplied at room temperature. After general work-up, the residue was purified by column chromatography on silica gel (hexane/EtOAc 2:1) to afford the title compound as colorless liquid (196 mg, 1.33 mmol, 89%). ^{1}H NMR (600 MHz, $CDCl_3$) δ 6.89 (d, *J* = 7.6 Hz, 1H), 6.86 (d, *J* = 7.6 Hz, 1H), 6.57 (td, *J* = 7.6, 1.0 Hz, 1H), 3.65 (brs, 1H), 3.38 (t, *J* = 6.2 Hz, 2H), 2.80 (t, *J* = 6.2 Hz, 2H), 2.09 (s, 3H), 1.95 (quint, *J* = 6.2 Hz, 2H); ^{13}C{^{1}H} NMR (150 MHz, $CDCl_3$) δ 142.8, 127.9, 127.5, 121.3, 121.0, 116.5, 42.5, 27.4, 22.3, 17.3; IR (neat) 3426, 2928, 2841, 2359, 1599, 1495, 1308, 1267, 1109, 759 cm^{-1}. The spectral data were in good agreement with literature.[4]

7-Methyl-1,2,3,4-tetrahydroquinoline (7c)

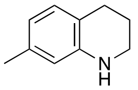

Prepared according to the general procedure from 7-methylquinoline (**6c**, 215 mg, 1.50 mmol) and *p*-toluenesulfonic acid monohydrate (28 mg, 0.15 mmol) in CH_2Cl_2 (3 mL). A constant current (25 mA/cm^2, 25 F/mol, 10 h) was supplied at room temperature. After general work-up, the residue was purified by column chromatography on silica gel (hexane/EtOAc 2:1) to afford the title compound as colorless liquid (198 mg, 1.35 mmol, 90%). ^{1}H NMR (600 MHz, $CDCl_3$) δ 6.89 (d, *J* = 7.6 Hz, 1H), 6.86 (d, *J* = 7.6 Hz, 1H), 6.57 (td, *J* = 7.6, 1.0 Hz, 1H), 3.65 (brs, 1H), 3.38 (t, *J* = 6.2 Hz, 2H), 2.80 (t, *J* = 6.2 Hz, 2H), 2.09 (s, 3H), 1.95 (quint, *J* = 6.2 Hz, 2H); ^{13}C{^{1}H} NMR (150 MHz, $CDCl_3$) δ 142.8, 127.9, 127.5, 121.3, 121.0, 116.5, 42.5, 27.4, 22.3, 17.3; IR (neat) 3426, 2928, 2841, 2359, 1599, 1495, 1308, 1267, 1109, 759 cm^{-1}. The spectral data were in good agreement with literature.[4]

6-Methyl-1,2,3,4-tetrahydroquinoline (7d)

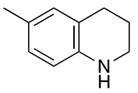

Prepared according to the general procedure from 6-methylquinoline (**6d**, 214 mg, 1.50 mmol) and *p*-toluenesulfonic acid monohydrate (29 mg, 0.15 mmol) in CH_2Cl_2 (3 mL). A constant current (25 mA/cm^2, 25 F/mol, 10 h) was supplied at room temperature. After general work-up, the residue was purified by column chromatography on silica gel (hexane/EtOAc 2:1) to afford the title compound as colorless solid (187 mg, 1.27 mmol, 85%). ^{1}H NMR (600 MHz, $CDCl_3$) δ 6.78–6.77 (m, 2H), 6.41–6.40 (m, 1H), 3.27 (t, *J* = 5.5 Hz, 2H), 2.73 (t, *J* = 6.5 Hz, 2H), 2.20 (s, 3H), 1.92 (tt, *J* = 6.5, 5.5 Hz, 2H); ^{13}C{^{1}H} NMR (150 MHz, $CDCl_3$) δ 142.5, 130.2, 127.4, 126.4, 121.7, 114.6, 42.3, 27.0, 22.5, 20.5; IR (KBr) 3364, 2911, 2837, 1616, 1512, 1350, 1306, 1260, 810, 635 cm^{-1}. The spectral data were in good agreement with literature.[3]

5-Methyl-1,2,3,4-tetrahydroquinoline (7e)

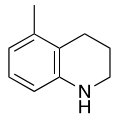

Prepared according to the general procedure from 5-methylquinoline (**6e**, 215 mg, 1.50 mmol) and *p*-toluenesulfonic acid monohydrate (29 mg, 0.15 mmol) in CH_2Cl_2 (3 mL). A constant current (25 mA/cm^2, 25 F/mol, 10 h) was supplied at room temperature. After general work-up, the residue was purified by column chromatography on silica gel (hexane/EtOAc 2:1) to afford the title compound as colorless liquid (192 mg, 1.30 mmol, 87%). ^{1}H NMR (600 MHz, $CDCl_3$) δ 6.89 (t, *J* = 7.3 Hz, 1H), 6.52 (d, *J* = 7.3 Hz, 1H), 6.38 (d, *J* = 7.3 Hz, 1H), 3.78 (brs, 1H), 3.26 (t, *J* = 5.5 Hz, 2H), 2.65 (t, *J* = 5.5 Hz, 2H), 2.18 (s, 3H), 1.99 (quint, *J* = 5.5 Hz, 2H); ^{13}C{^{1}H} NMR (150 MHz, $CDCl_3$) δ 145.1, 137.4, 126.3, 120.4, 119.1, 112.6, 41.7, 24.2, 22.7, 19.5; IR (neat) 3401, 3046, 2941, 1589, 1348, 1306, 1269, 1113, 768, 714 cm^{-1}. The spectral data were in good agreement with literature.[4]

3-Methyl-1,2,3,4-tetrahydroquinoline (7g)

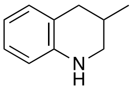

Prepared according to the general procedure from 3-methylquinoline (**6g**, 215 mg, 1.50 mmol) and *p*-toluenesulfonic acid monohydrate (29 mg, 0.15 mmol) in CH_2Cl_2 (3 mL). A constant current (25 mA/cm^2, 25 F/mol, 10 h) was supplied at room temperature. After general work-up, the residue was purified by column chromatography on silica gel (hexane/EtOAc 2:1) to afford the title compound as colorless liquid (188 mg, 1.28 mmol, 85%). ^{1}H NMR (600 MHz, $CDCl_3$) δ 6.96 (t, *J* = 7.4 Hz, 1H), 6.94 (d, *J* = 7.4 Hz, 1H), 6.60 (td, *J* = 7.4, 0.9 Hz, 1H), 6.48 (d, *J* = 7.4 Hz, 1H), 3.45 (brs, 1H), 3.27 (ddd, *J* = 10.6, 3.7, 1.8 Hz, 1H), 2.89 (t, *J* = 10.6 Hz, 1H), 2.77 (dd, *J* = 16.1, 5.0 Hz, 1H), 2.43 (dd, *J* = 16.1, 10.6 Hz, 1H), 2.10–2.02 (m, 1H), 1.05 (d, *J* = 6.9 Hz, 3H); ^{13}C{^{1}H} NMR (150 MHz, $CDCl_3$) δ 144.3, 129.4, 126.6, 120.9, 116.7, 113.8, 48.7, 35.4, 27.1, 19.0; IR (neat) 3410, 2955, 2832, 1607, 1504, 1371, 1273, 1153, 835, 746 cm^{-1}. The spectral data were in good agreement with literature.[4]

2-Methyl-1,2,3,4-tetrahydroquinoline (7h)

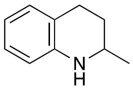

Prepared according to the general procedure from 2-methylquinoline (**6h**, 215 mg, 1.50 mmol) and *p*-toluenesulfonic acid monohydrate (29 mg, 0.15 mmol) in CH_2Cl_2 (3 mL). A constant current (25 mA/cm^2, 25 F/mol, 10 h) was supplied at room temperature. After general work-up, the residue was purified by column chromatography on silica gel (hexane/EtOAc 2:1) to afford the title compound as colorless liquid (189 mg, 1.28 mmol, 85%). ^{1}H NMR (600 MHz, $CDCl_3$) δ 6.98–6.96 (m, 2H), 6.61 (td, *J* = 7.4, 1.4 Hz, 1H), 6.48 (dd, *J* = 7.4, 1.4 Hz, 1H), 3.40 (dqd, *J* = 10.0, 6.2, 3.4 Hz, 1H), 2.85 (ddd, *J* = 16.2, 11.7, 5.2 Hz, 1H), 2.74 (ddd, *J* = 16.2, 5.2, 3.4 Hz, 1H), 1.94 (ddt, *J* = 12.5, 5.2, 3.4 Hz, 1H), 1.60 (dddd, *J* = 12.5, 11.7, 10.0, 5.2 Hz, 1H), 1.21 (d, *J* = 6.2 Hz, 3H); ^{13}C{^{1}H} NMR (150 MHz, $CDCl_3$) δ 144.7, 129.2, 126.6, 120.9, 116.8, 114.0, 46.9, 30.1, 26.5, 22.5; IR (neat) 3393, 2843,

1609, 1487, 1377, 1310, 1258, 1153, 1124, 746 cm^{-1}. The spectral data were in good agreement with literature.[3]

2,3-Dimethyl-1,2,3,4-tetrahydroquinoline (7i)

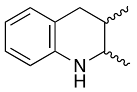

Prepared according to the general procedure from 2,3-dimethylquinoline (**6i**, 235 mg, 1.49 mmol) and *p*-toluenesulfonic acid monohydrate (28 mg, 0.15 mmol) in CH_2Cl_2 (3 mL). A constant current (25 mA/cm^2, 25 F/mol, 10 h) was supplied at room temperature. After general work-up, the residue was purified by column chromatography on silica gel (hexane/EtOAc 2:1) to afford the title compound as yellow liquid (185 mg, 1.15 mmol, 77%). ^{1}H NMR (600 MHz, $CDCl_3$) δ 6.98 (t, *J* = 7.8 Hz, 1H), 6.97 (dd, *J* = 7.8, 3.2 Hz, 1H), 6.63–6.60 (m, 1H), 6.48 (d, *J* = 7.8 Hz, 1H), 3.69 (brs, 1H), 3.47 (qd, *J* = 6.4, 3.2 Hz, 1H, *cis* isomer), 3.04–3.00 (m, 1H, *trans* isomer), 2.92 (dd, *J* = 16.1, 5.0 Hz, 1H, *cis* isomer), 2.74 (dd, *J* = 16.1, 5.0 Hz, 1H, *trans* isomer), 2.50 (dd, *J* = 16.5, 6.7 Hz, 1H), 2.08–2.02 (m, 1H, *cis* isomer), 1.68–1.60 (m, 1H, *trans* isomer), 1.21 (d, *J* = 6.4 Hz, 3H, *trans* isomer), 1.13 (d, *J* = 6.4 Hz, 3H, *cis* isomer), 1.04 (d *J* = 6.4 Hz, 3H, *trans* isomer), 0.95 (d, *J* = 6.4 Hz, 3H, *cis* isomer); ^{13}C{^{1}H} NMR (150 MHz, $CDCl_3$) δ 144.6, 144.0, 129.9, 129.2, 126.8, 126.7, 121.5, 120.2, 117.0, 116.9, 114.0, 113.5, 53.4, 50.0, 35.6, 33.9, 33.6, 30.6, 20.9, 18.4, 18.2, 14.5; IR (neat) 3397, 2968, 2899, 2837, 1609, 1381, 1314, 1279, 1155, 748 cm^{-1}. The spectral data were in good agreement with literature.[5]

2-Phenyl-1,2,3,4-tetrahydroquinoline (7j)

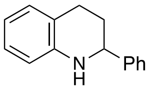

Prepared according to the general procedure from 2-phenylquinoline (**6j**, 308 mg, 1.50 mmol) and *p*-toluenesulfonic acid monohydrate (28 mg, 0.15 mmol) in CH_2Cl_2 (3 mL). A constant current (25 mA/cm^2, 25 F/mol, 10 h) was supplied at room temperature. After general work-up, the residue was purified by column chromatography on silica gel (hexane/EtOAc 2:1) to afford the title compound as colorless oil (256 mg, 1.22 mmol, 81%). ^{1}H NMR (600 MHz, $CDCl_3$) δ 7.39 (d, *J* = 6.9 Hz, 2H), 7.35 (t, *J* = 6.9 Hz, 2H), 7.28 (t, *J* = 6.9 Hz, 1H), 7.01 (t, *J* = 7.6 Hz, 1H), 7.00 (d, *J* = 7.6 Hz, 1H), 6.65 (t, *J* = 7.6 Hz, 1H), 6.54 (d, *J* = 7.6 Hz, 1H), 4.43 (dd, *J* = 10.7, 5.5 Hz, 1H), 4.03 (brs, 1H), 2.92 (ddd, *J* = 16.5, 10.7, 5.5 Hz, 1H), 2.73 (dt, *J* = 16.5, 5.5 Hz, 1H), 2.14–2.10 (m, 1H), 2.02–1.96 (m, 1H); ^{13}C{^{1}H} NMR (150 MHz, $CDCl_3$) δ 144.9, 144.8, 129.4, 128.6, 127.5, 127.0, 126.6, 120.9, 117.2, 114.1, 56.3, 31.0, 26.4; IR (neat) 3406, 3383, 3015, 2945, 2926, 2841, 1113, 926, 758, 700 cm^{-1}. The spectral data were in good agreement with literature.[3]

6-Acetyl-1,2,3,4-tetrahydroquinoline (7k)

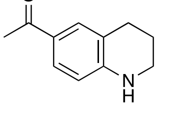

Prepared according to the general procedure from 6-acetylquinoline (**6k**, 256 mg, 1.50 mmol) and *p*-toluenesulfonic acid monohydrate (28 mg, 0.15 mmol) in CH_2Cl_2 (12 mL). A constant current (25 mA/cm^2, 25 F/mol, 10 h) was supplied at room temperature. After general work-up, the residue was purified by column chromatography on silica gel (hexane/EtOAc 2:1) to afford the title compound as colorless solid (210 mg, 1.20 mmol, 80%). ^{1}H NMR (600 MHz, $CDCl_3$) δ 7.62–7.60 (m, 2H), 6.40 (dd, *J* = 6.4, 2.8 Hz 1H), 4.40 (brs, 1H), 3.37 (td, *J* = 6.0, 2.8 Hz 2H), 2.78 (t, *J* = 6.0 Hz, 2H), 2.45 (s, 3H), 1.94 (quint, *J* = 6.0 Hz, 2H); ^{13}C{^{1}H} NMR (150 MHz, $CDCl_3$) δ 196.4, 149.1, 1330.5, 128.6, 126.1, 119.8, 112.5, 41.7, 27.0, 26.0, 21.4; IR (KBr) 3339, 1647, 1580, 1533, 1435, 1319, 1290, 1236, 1146, 827 cm^{-1}; HRMS (ESI) *m/z* calcd for $C_{11}H_{13}NNaO$ $[M + Na]^+$ 198.0889, found 198.0880; mp 101.5–102.9 °C.

Methyl 1,2,3,4-tetrahydroquinoline-6-carboxylate (7l)

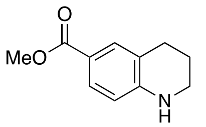

Prepared according to the general procedure from methyl quinoline-6-carboxylate (**6l**, 281 mg, 1.50 mmol) and *p*-toluenesulfonic acid monohydrate (29 mg, 0.15 mmol) in CH_2Cl_2 (6 mL). A constant current (25 mA/cm^2, 25 F/mol, 10 h) was supplied at room temperature. After general work-up, the residue was purified by column chromatography on silica gel (hexane/EtOAc 2:1) to afford the title compound as colorless solid (141 mg, 0.74 mmol, 49%). ^{1}H NMR (600 MHz, $CDCl_3$) δ 7.65–7.64 (m, 2H), 6.40–6.38 (m, 1H), 4.30 (brs, 1H), 3.83 (s, 3H), 3.36 (t, *J* = 6.2 Hz, 2H), 2.77 (t, *J* = 6.2 Hz, 2H), 1.93 (quint, *J* = 6.2 Hz, 2H); ^{13}C{^{1}H} NMR (150 MHz, $CDCl_3$) δ 167.6, 148.1, 131.5, 129.2, 120.7, 118.3, 113.4, 51.7, 41.9, 26.9, 21.4; IR (KBr) 3381, 2943, 1682, 1605, 1531, 1441, 1325, 1288, 1234, 1144 cm^{-1}. The spectral data were in good agreement with literature.[4]

1,2,3,4-Tetrahydroquinoline-6-carboxamide (7m)

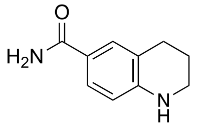

Prepared according to the general procedure from methyl quinoline-6-carboxamide (**6m**, 258 mg, 1.50 mmol) and *p*-toluenesulfonic acid monohydrate (29 mg, 0.15 mmol) in 1,4-dioxane/H_2O 7:1 (6 mL). A constant current (25 mA/cm^2, 25 F/mol, 10 h) was supplied at room temperature. After general work-up, the residue was purified by column chromatography on silica gel (EtOAc) to afford the title compound as colorless solid (230 mg, 1.31 mmol, 87%). ^{1}H NMR (600 MHz, $CDCl_3$) δ 7.46–7.46 (m, 1H), 7.41 (dd, *J* = 8.3, 2.1 Hz 1H), 6.41 (d, *J* = 8.3 Hz 1H), 4.26 (brs, 1H), 3.35 (td, *J* = 6.2, 2.4 Hz 2H), 2.78 (t, *J* = 6.2 Hz, 2H), 1.93 (quint, *J* = 6.2 Hz, 2H); ^{13}C{^{1}H} NMR (150 MHz, $CDCl_3$) δ 196.6, 148.2, 129.5, 126.8, 120.6, 120.4, 112.9, 41.9, 27.1, 21.6; IR (KBr) 3343, 2943, 2839, 1647, 1611, 1523, 1437, 1387, 1317, 772 cm^{-1}. The spectral data were in good agreement with literature.[6]

6-(Trifluoromethyl)-1,2,3,4-tetrahydroquinoline (7n)

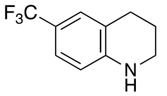

Prepared according to the general procedure from methyl 6-(trifluiromethyl)quinoline (**6n**, 296 mg, 1.50 mmol) and *p*-toluenesulfonic acid monohydrate (30 mg, 0.16 mmol) in CH_2Cl_2 (3 mL). A constant current (25 mA/cm^2, 25 F/mol, 10 h) was supplied at room temperature. After general work-up, the residue was purified by column chromatography on silica gel (hexane/EtOAc 2:1) to afford the title compound as colorless liquid (244 mg, 1.21 mmol, 81%). ^{1}H NMR (600 MHz, $CDCl_3$) δ 7.17 (d, *J* = 7.9 Hz, 1H), 7.17 (s, 1H), 6.44 (d, *J* = 7.9 Hz, 1H), 4.15 (brs, 1H), 3.34 (t, *J* = 6.5 Hz, 2H), 2.77 (t, *J* = 6.5 Hz, 2H), 1.96–1.92 (m, 2H); ^{13}C{^{1}H} NMR (150 MHz, $CDCl_3$) δ 147.5, 126.5 (q, $^3J_{C-F}$ = 4.3 Hz), 125.3 (q, $^1J_{C-F}$ = 270.2 Hz), 124.1 (q, $^3J_{C-F}$ = 4.3 Hz), 120.1, 117.9 (q, $^2J_{C-F}$ = 31.8 Hz), 113.1, 41.7, 27.0, 21.5; ^{19}F NMR (565 MHz, $CDCl_3$) δ −62.1; IR (neat) 3422, 2957, 2936, 2843, 1620, 1526, 1331, 1246, 1099, 816 cm^{-1}. The spectral data were in good agreement with literature.[7]

8-Methoxy-1,2,3,4-tetrahydroquinoline (7o)

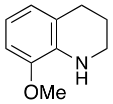

Prepared according to the general procedure from 8-methoxyquinoline (**6o**, 238 mg, 1.49 mmol) and *p*-toluenesulfonic acid monohydrate (28 mg, 0.15 mmol) in CH_2Cl_2 (3 mL). A constant current (25 mA/cm^2, 25 F/mol, 10 h) was supplied at room temperature. After general work-up, the residue was purified by column chromatography on silica gel (hexane/EtOAc 2:1) to afford the title compound as colorless liquid (214 mg, 1.31 mmol, 88%). ^{1}H NMR (600 MHz, $CDCl_3$) δ 6.63–6.61 (m, 2H), 6.57 (td, *J* = 8.3, 0.9 Hz, 1H), 4.23 (brs, 1H), 3.83 (s, 3H), 3.34 (t, *J* = 6.0 Hz, 2H), 2.78 (t, *J* = 6.0 Hz, 2H), 1.96 (quint, *J* = 6.0 Hz, 2H); ^{13}C{^{1}H} NMR (150 MHz, $CDCl_3$) δ 146.4, 134.6, 121.8, 121.5, 115.8, 107.5, 55.5, 41.6, 26.8, 22.2; IR (neat) 3418, 2947, 2833, 1612, 1587, 1331, 1248, 1267, 1192, 1105 cm^{-1}. The spectral data were in good agreement with literature.[4]

6-Methoxy-1,2,3,4-tetrahydroquinoline (7p)

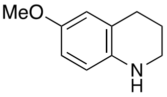

Prepared according to the general procedure from 6-methoxyquinoline (**6p**, 239 mg, 1.50 mmol) and *p*-toluenesulfonic acid monohydrate (28 mg, 0.15 mmol) in CH_2Cl_2 (3 mL). A constant current (25 mA/cm^2, 25 F/mol, 10 h) was supplied at room temperature. After general work-up, the residue was purified by column chromatography on silica gel (hexane/EtOAc 2:1) to afford the title compound as colorless solid (3 mg, 0.02 mmol, 1%). ^{1}H NMR (600 MHz, $CDCl_3$) δ 6.59 (dd, *J* = 8.6, 3.1 Hz, 1H), 6.56 (d, *J* = 3.1 Hz, 1H), 6.46 (d, *J* = 8.6 Hz, 1H), 3.73 (s, 3H), 3.25 (t, *J* = 5.5 Hz, 2H), 2.75 (t, *J* = 6.5 Hz, 2H), 1.93 (tt, *J* = 6.5, 5.5 Hz, 2H); ^{13}C{^{1}H} NMR (150 MHz, $CDCl_3$) δ 152.0, 138.9, 123.1, 115.8, 115.0, 113.0, 55.9, 42.5, 27.3, 22.5; IR (KBr) 3367, 2361, 2332, 1506, 1298, 1254, 1233,

1152, 1040, 810 cm^{-1}. The spectral data were in good agreement with literature.[3]

3-Methoxy-1,2,3,4-tetrahydroquinoline (7q)

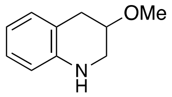

Prepared according to the general procedure from 3-methoxyquinoline (**6q**, 238 mg, 1.49 mmol) and *p*-toluenesulfonic acid monohydrate (28 mg, 0.15 mmol) in CH_2Cl_2 (3 mL). A constant current (25 mA/cm^2, 25 F/mol, 10 h) was supplied at room temperature. After general work-up, the residue was purified by column chromatography on silica gel (hexane/EtOAc 2:1) to afford the title compound as yellow liquid (37 mg, 0.23 mmol, 15%). ^{1}H NMR (600 MHz, $CDCl_3$) δ 6.99–6.97 (m, 2H), 6.64 (td, J = 7.5, 1.2 Hz 1H), 6.51 (d, J = 7.5 Hz 1H), 3.76 (dddd, J = 10.6, 6.9, 4.1, 3.2 Hz 1H), 3.45 (s, 3H), 3.43 (dd, J = 3.2, 1.4 Hz 1H), 3.22 (ddd, J = 10.6, 6.9, 1.4 Hz 1H), 3.03 (dd, J = 15.8, 4.1 Hz 1H), 2.81 (dd, J = 15.8, 6.9 Hz 1H); $^{13}C\{^1H\}$ NMR (150 MHz, $CDCl_3$) δ 143.9, 130.2, 127.2, 119.1, 117.8, 114.2, 72.9, 56.4, 45.3, 32.8; IR (neat) 3383, 2826, 2359, 1607, 1585, 1285, 1200, 1096, 984, 748 cm^{-1}; HRMS (ESI) *m/z* calcd for $C_{11}H_{13}NNaO$ $[M + Na]^+$ 186.0889, found 186.0887.

4-Methoxy-5,6,7,8-tetrahydroquinoline (7r′)

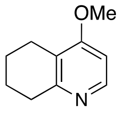

Prepared according to the general procedure from 4-methoxyquinoline (**6r**, 239 mg, 1.50 mmol) and *p*-toluenesulfonic acid monohydrate (29 mg, 0.15 mmol) in CH_2Cl_2 (3 mL). A constant current (25 mA/cm^2, 25 F/mol, 10 h) was supplied at room temperature. After general work-up, the residue was purified by column chromatography on silica gel (hexane/EtOAc 2:1) to afford the title compound as yellow liquid (11 mg, 0.07 mmol, 5%). ^{1}H NMR (600 MHz, $CDCl_3$) δ 8.25 (d, J = 6.0 Hz, 1H), 6.58 (d, J = 6.0 Hz, 1H), 3.84 (s, 3H), 2.86 (t, J = 6.0 Hz, 2H), 2.61 (t, J = 6.0 Hz, 2H), 1.86–1.75 (m, 4H); $^{13}C\{^1H\}$ NMR (150 MHz, $CDCl_3$) δ 163.6, 157.9, 148.0, 121.3, 103.3, 55.3, 32.6, 22.9, 22.34, 22.30; IR (neat) 3383, 3308, 2938, 1576, 1337, 1290, 1111, 1090, 997, 810 cm^{-1}. The spectral data were in good agreement with literature.[8]

6-Fluoro-1,2,3,4-tetrahydroquinoline (7s)

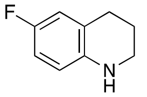

Prepared according to the general procedure from 6-fluoroquinoline (**6s**, 221 mg, 1.50 mmol) and *p*-toluenesulfonic acid monohydrate (29 mg, 0.15 mmol) in CH_2Cl_2 (3 mL). A constant current (25 mA/cm^2, 25 F/mol, 10 h) was supplied at room temperature. After general work-up, the residue was purified by column chromatography

on silica gel (hexane/EtOAc 2:1) to afford the title compound as yellow liquid (203 mg, 1.34 mmol, 89%). ^{1}H NMR (600 MHz, $CDCl_3$) δ 6.70–6.66 (m, 2H), 6.40 (dd, J = 9.4, 5.0 Hz, 1H), 3.66 (brs, 1H), 3.27 (t, J = 5.5 Hz, 2H), 2.74 (t, J = 5.5 Hz, 2H), 1.92 (quint, J = 5.5 Hz, 2H); ^{13}C{^{1}H} NMR (150 MHz, $CDCl_3$) δ 155.6 (d, $^{1}J_{C–F}$ = 234.1 Hz), 141.0, 122.9 (d, $^{4}J_{C–F}$ = 5.8 Hz), 115.7 (d, $^{2}J_{C–F}$ = 21.7 Hz), 115.0 (d, $^{3}J_{C–F}$ = 7.2 Hz), 113.3 (d, $^{2}J_{C–F}$ = 21.7 Hz), 42.2, 27.1, 22.1; ^{19}F NMR (565 MHz, $CDCl_3$) δ −129.6; IR (KBr) 3385, 2363, 1508, 1306, 1248, 1221, 1140, 926, 887, 772 cm^{-1}. The spectral data were in good agreement with literature.[9]

6-Chloro-1,2,3,4-tetrahydroquinoline (7t)

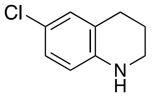

Prepared according to the general procedure from 6-chloroquinoline (**6t**, 246 mg, 1.50 mmol) and *p*-toluenesulfonic acid monohydrate (29 mg, 0.15 mmol) in CH_2Cl_2 (3 mL). A constant current (25 mA/cm^{2}, 25 F/mol, 10 h) was supplied at room temperature. After general work-up, the residue was purified by column chromatography on silica gel (hexane/EtOAc 2:1) to afford the title compound as colorless liquid (202 mg, 1.21 mmol, 80%). ^{1}H NMR (600 MHz, $CDCl_3$) δ 6.91–6.89 (m, 2H), 6.38 (d, J = 8.3 Hz, 1H), 3.81 (brs, 1H), 3.28 (t, J = 6.4 Hz, 2H), 2.72 (t, J = 6.4 Hz, 2H), 1.91 (quint, J = 6.4 Hz, 2H); ^{13}C{^{1}H} NMR (150 MHz, $CDCl_3$) δ 143.4, 129.2, 126.6, 123.0, 121.3, 115.2, 42.0, 27.0, 21.9; IR (neat) 3418, 2841, 1603, 1497, 1470, 1352, 1300, 1269, 1184, 806 cm^{-1}. The spectral data were in good agreement with literature.[3]

Table S1. Effect of current density on the electrocatalytic reduction of **6a** using a PEM reactor

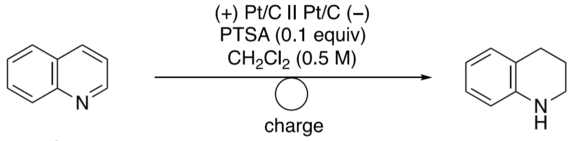

6a **7a**

Experimental conditions: cell temperature, rt; current density; flow rate of solution, 0.75 mL/min; flow rate of hydrogen, 100 mL/min.

entry	current density (mA/cm^{2})	charge (F/mol)	yield of 7**a** (%)^{a}	recov. of 6**a** (%)^{a}	current efficiency (%)^{a}
1 (10 runs)	50	50	85–94	N.D.^{b}	7–8
2	2.5	2.5	40	53	64
3	5	5	55	36	44
4	10	10	72	16	29
5	20	20	87	2	17
6	25	25	93	N.D.	15

^{a} Determined by ^{1}H NMR analysis using 1,1,2,2-tetrachloroethane as an internal standard. ^{b} Not Detected.

Table S2. Time course of the electrocatalytic reduction of **6a** using a PEM reactor

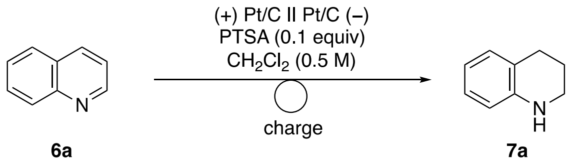

Experimental conditions: cell temperature, rt; current density, 25 mA/cm^2; flow rate of solution, 0.75 mL/min; flow rate of hydrogen, 100 mL/min.

charge (F/mol)	yield of **2a** (%)[a]	recov. of **1a** (%)[a]	current efficiency of **1a** (%)[a]
5	55	42	44
10	82	14	33
15	87	6	23
20	91	2	18
25	93	N.D.[b]	15

[a] Determined by ^{1}H NMR analysis using 1,1,2,2-tetrachloroethane as an internal standard.
[b] Not Detected.

General Procedure for hydrogenation of pyridines using a PEM reactor

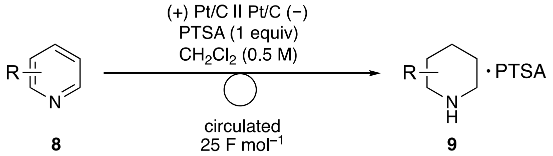

Experimental conditions: cell temperature, rt; current density, 25 mA/cm^2; flow rate of solution, 0.75 mL/min; flow rate of hydrogen, 100 mL/min.

Electrocatalytic hydrogenations were carried out by using a PEM reactor (Figure S2). The MEA (3 cm × 10 cm, 1 cm × 4 cm: active area) was integrated into the PEM reactor. For the cathodic chamber, humidified nitrogen gas was supplied for 1 hour. After that, the solution of substrate **8** (1.5 mmol) and *p*-toluenesulfonic acid monohydrate (1.5 mmol, 1 equiv) in CH_2Cl_2 (3 mL) was provided with 0.75 mL/min by a circular pump (YAMAZEN, MSP-001-0). For the anodic chamber, humidified hydrogen gas (flow rate: 100 mL/min) was supplied. A constant current (25 mA/cm^2, 25 F/mol, 10 h) was supplied at room temperature. The yield of **9**•PTSA was determined by ^{1}H NMR analysis of the obtained cathodic reaction solution using 1,1,2,2-tetrachloroethane or ethylene carbonate as an internal standard. The spectra data were in good agreement with authentic samples.

Procedure for Hydrogenation of Quinoline 6a in a PEM Reactor Using H_2O as a H^+ Source

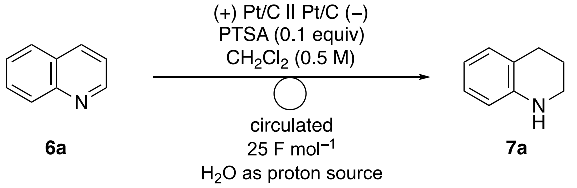

Experimental conditions: cell temperature, rt; current density, 25 mA/cm^2; flow rate of solution, 0.75 mL/min; flow rate of H_2O, 1.0 mL/min.

Electrocatalytic hydrogenations were carried out by using a PEM reactor (Figure S3). The MEA (3 cm × 10 cm, 1 cm × 4 cm: active area) including a DSE® electrode as an anolyte was integrated into the PEM reactor. For the

cathodic chamber, the solution of quinoline **6a** (193 mg, 1.5 mmol) and *p*-toluenesulfonic acid monohydrate (29 mg, 0.15 mmol) in CH_2Cl_2 (3 mL) was provided with 0.75 mL/min by a circular pump (YAMAZEN, MSP-001-0). For the anodic chamber, H_2O was provided with 1.0 mL/min by a circular pump. A constant current (25 mA/cm^2, 25 F/mol, 10 h) was supplied at room temperature. To the obtained cathodic reaction solution was added NaOH aq (1.0 M, 5 mL), and the mixture was extracted with CH_2Cl_2 (3 × 10 mL). The combined organic phase was washed with brine, dried over Na_2SO_4, filtered, and concentrated under reduced pressure. The residue was purified by column chromatography on silica gel eluting with hexane/EtOAc 2:1 to afford 1,2,3,4-tetrahydroquinoline (**7a**, 110 mg, 0.82 mmol, 55%).

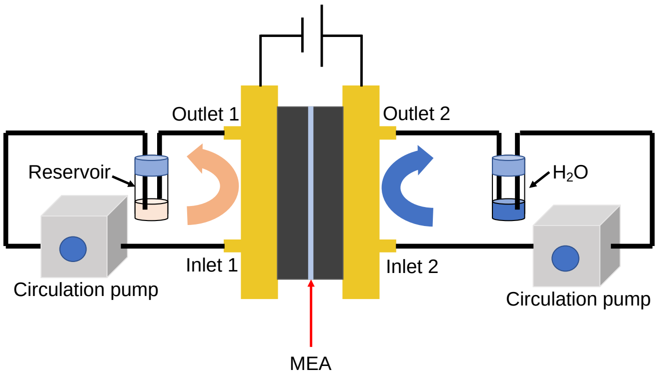

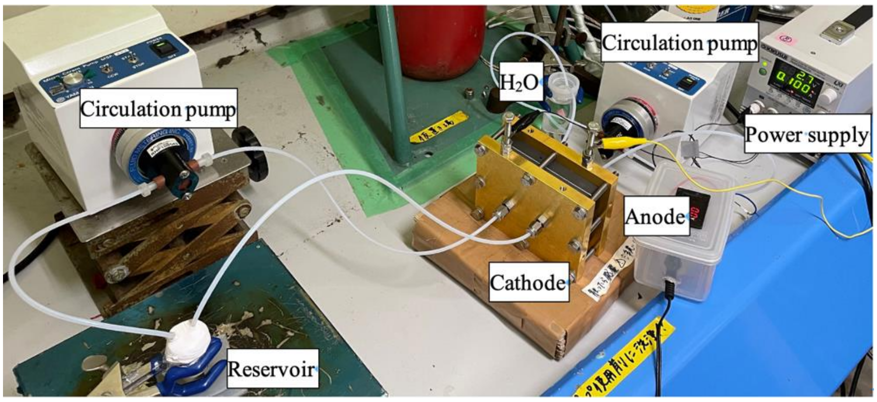

Figure S3. Hydrogenation system in a PEM reactor using H_2O as a proton source.

Procedure for hydrogenation of quinoline (6a) in a PEM reactor using 2 M H_2SO_4 as a H^+ source

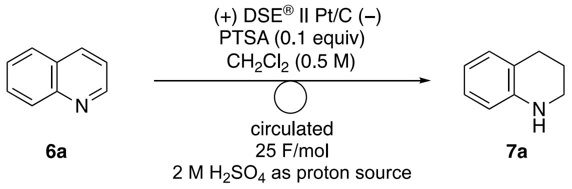

Experimental conditions: cell temperature, rt; current density, 25 mA/cm^2; flow rate of solution, 0.75 mL/min; flow rate of 2 MH_2SO_4 aq,1.0 mL/min.

Electrocatalytic hydrogenations were carried out by using a PEM reactor. The MEA (3 cm × 10 cm, 1 cm × 4 cm: active area) including a DSE® electrode as an anolyte was integrated into the PEM reactor. For the cathodic chamber, the solution of quinoline **6a** (193 mg, 1.5 mmol) and *p*-toluenesulfonic acid monohydrate (29 mg, 0.15 mmol) in CH_2Cl_2 (3 mL) was provided with 0.75 mL/min by a circular pump (YAMAZEN, MSP-001-0). For the anodic chamber, 2 M H_2SO_4 was provided with 1.0 mL/min by a circular pump. A constant current (25 mA/cm^2, 25 F/mol, 10 h) was supplied at room temperature. To the obtained cathodic reaction solution was added NaOH aq (1.0 M, 5 mL), and the mixture was extracted with CH_2Cl_2 (3 × 10 mL). The combined organic phase was washed with brine, dried over Na_2SO_4, filtered, and concentrated under reduced pressure. The residue was purified by column chromatography on silica gel eluting with hexane/EtOAc 2:1 to afford 1,2,3,4-tetrahydroquinoline (**7a**, 156 mg, 1.2 mmol, 80%).

Procedure for the hydrogenation of pyridine (8a) in a PEM reactor using 2 M H_2SO_4 as a proton source.

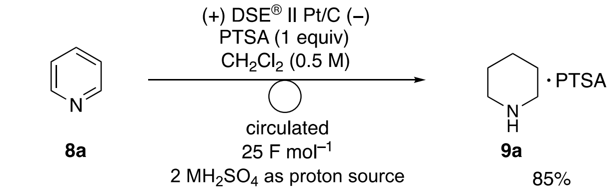

Experimental conditions: cell temperature, rt; current density, 25 mA/cm^2; flow rate of solution, 0.75 mL/min; flow rate of 2 M H_2SO_4 aq, 1.0 mL/min.

Electrocatalytic hydrogenations were carried out by using a PEM reactor. The MEA (3 cm × 10 cm, 1 cm × 4 cm: active area) including a DSE® electrode as an anolyte was integrated into the PEM reactor. For the cathodic chamber, the solution of pyridine **8a** (119 mg, 1.5 mmol) and *p*-toluenesulfonic acid monohydrate (286 mg, 1.5 mmol) in CH_2Cl_2 (3 mL) was provided with 0.75 mL/min by a circular pump (YAMAZEN, MSP-001-0). For the anodic chamber, 2 M H_2SO_4 aq was provided with 1.0 mL/min by a circular pump. A constant current (25 mA/cm^2, 25 F/mol, 10 h) was supplied at room temperature. The yield of **9a**•PTSA was determined by the ^{1}H NMR analysis of the obtained cathodic reaction solution using 1,1,2,2-tetrachloroethane as an internal standard (85% yield).

References

(1) Anand, N.; Koley, S.;Ramulu, J.; Singh, M. S. *Org. Biomol. Chem.* **2015**, *13*, 9570–9574.

(2) Ma, W.; Zhang, J.; Xu, C.; Chen, F.; He, Y.-M.; Fan, Q.-H. *Angew. Chem., Int. Ed.* **2016**, *55*, 12891–12894.

(3) Duan, Y.-N.; Du, Xi.; Cui, Z.; Zeng, Y.; Liu, Y.; Yang, T.; Wen, J.; Zhang, X. *J. Am. Chem. Soc.* **2019**, *141*, 20424–20433.

(4) Yang, C.-H.; Chen, X.; Li, H.; Wei, W.; Yang, Z.; Chang, J. *Chem. Commun*. **2018**, *54*, 8622–8625.

(5) Wu, J.; Wang, C.; Tang, W.; Pettman, A.; Xiao, J. *Chem. Eur. J.* **2012**, *18*, 9525–9529.

(6) Sorribes, I.; Liu, L.; Doménech-Carbó, A.; Corma, A. *ACS Catal*. **2018**, *8*, 4545–4557.

(7) Compain, G.; Bonneau, C.; Martin-Mingot, A.; Thibaudeau, S. *J. Org. Chem.* **2013**, *78*, 4463–4472.

(8) Kuwano, R.; Ikeda, R.; Hirasada, K. *Chem. Commun*. **2015**, *51*, 7558–7561.

(9) Yadav, S.; Chaudhary, D.; Maurya, N. K., Kumar, D.; Ishu, K.; Kuram, M. R. *Chem. Commun*. **2022**, *58*, 4255–4258.

1,2,3,4-Tetrahydroquinoline (7a) ^{1}H NMR (600 MHz, $CDCl_3$)

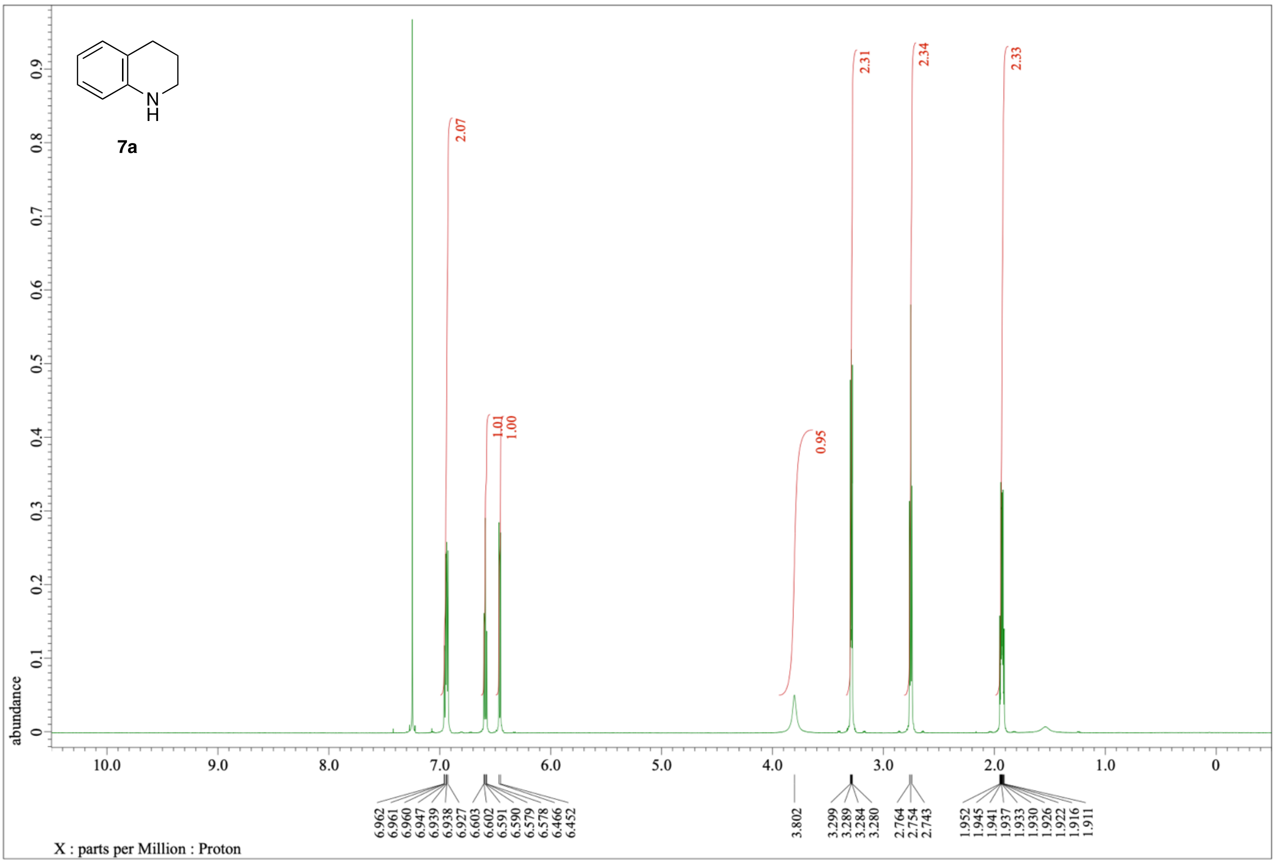

1,2,3,4-Tetrahydroquinoline (7a) ^{13}C{^{1}H} NMR (150 MHz, $CDCl_3$)

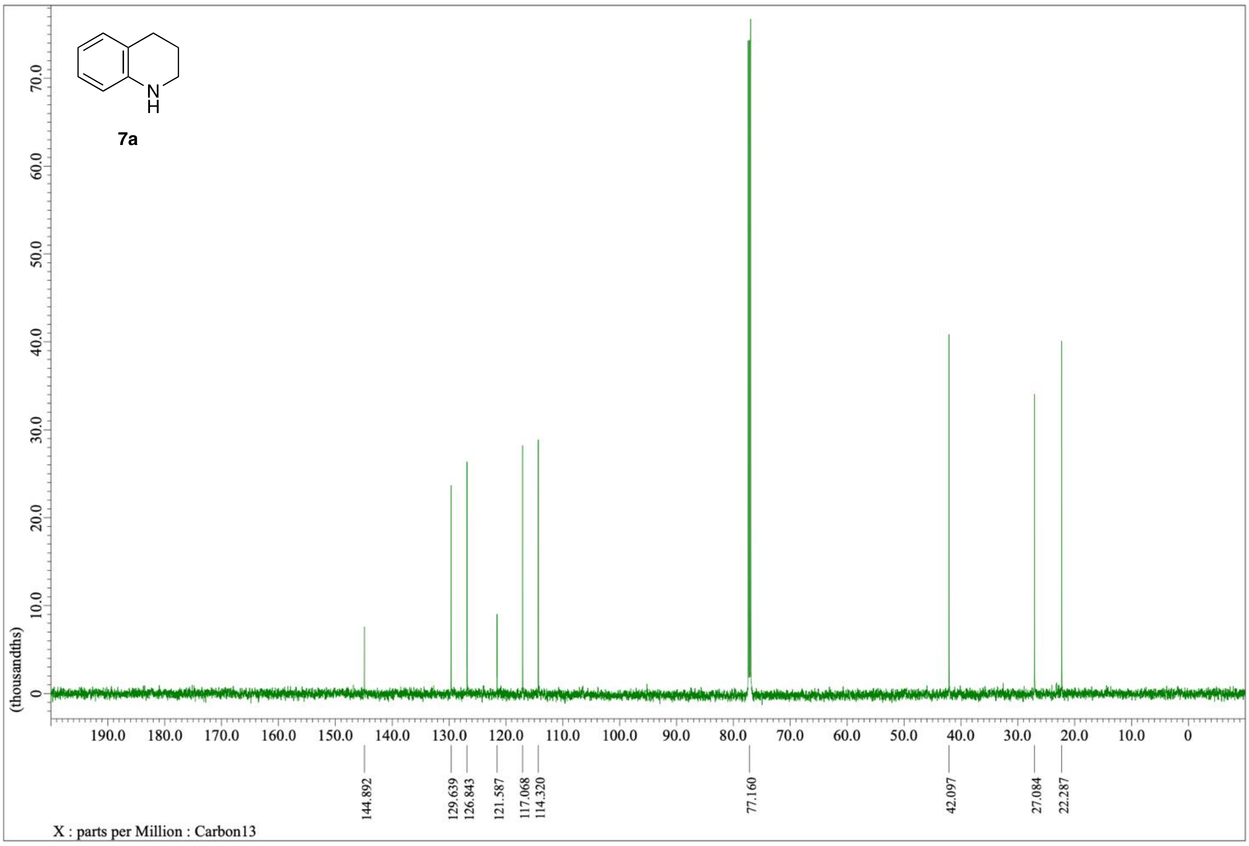

8-Methyl-1,2,3,4-tetrahydroquinoline (7b) ^{1}H NMR (600 MHz, $CDCl_3$)

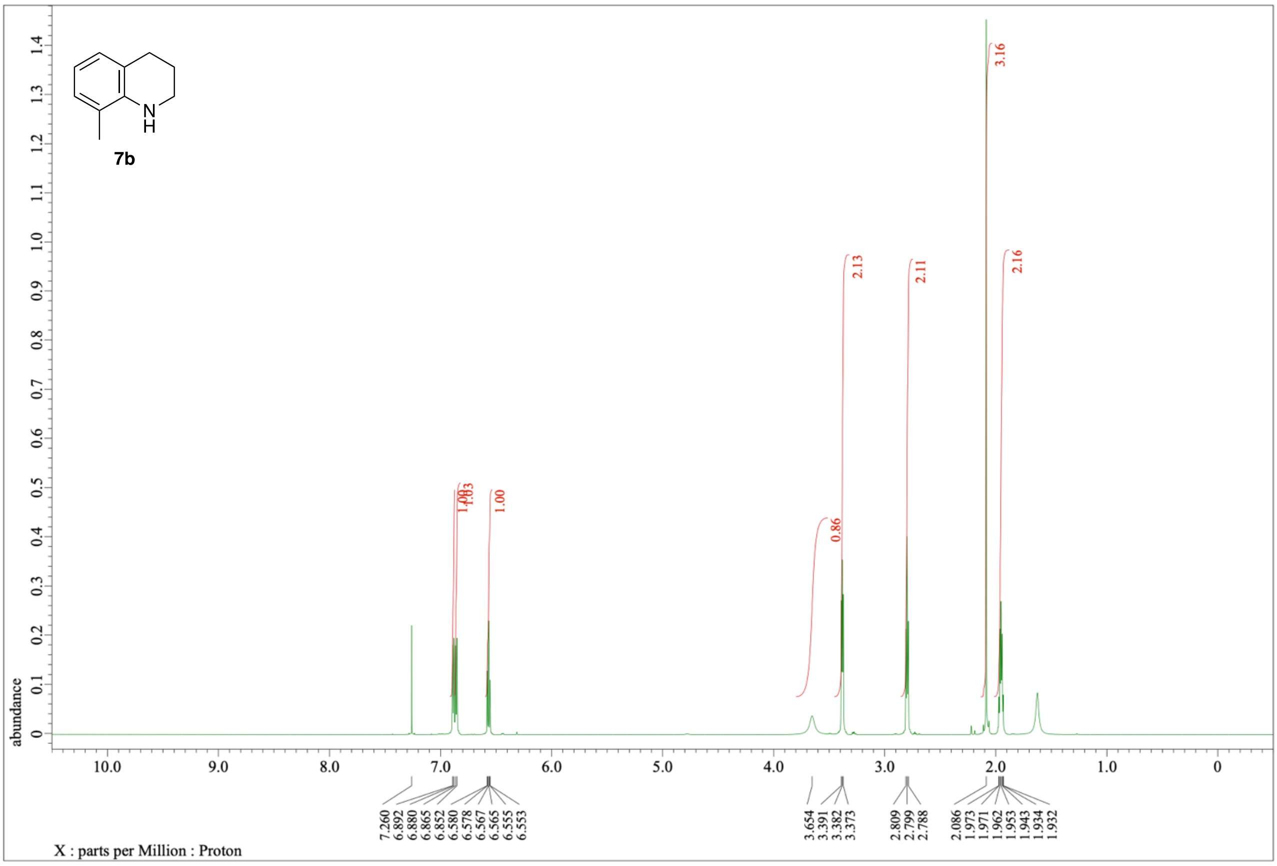

8-Methyl-1,2,3,4-tetrahydroquinoline (7b) $^{13}C\{^1H\}$ NMR (150 MHz, $CDCl_3$)

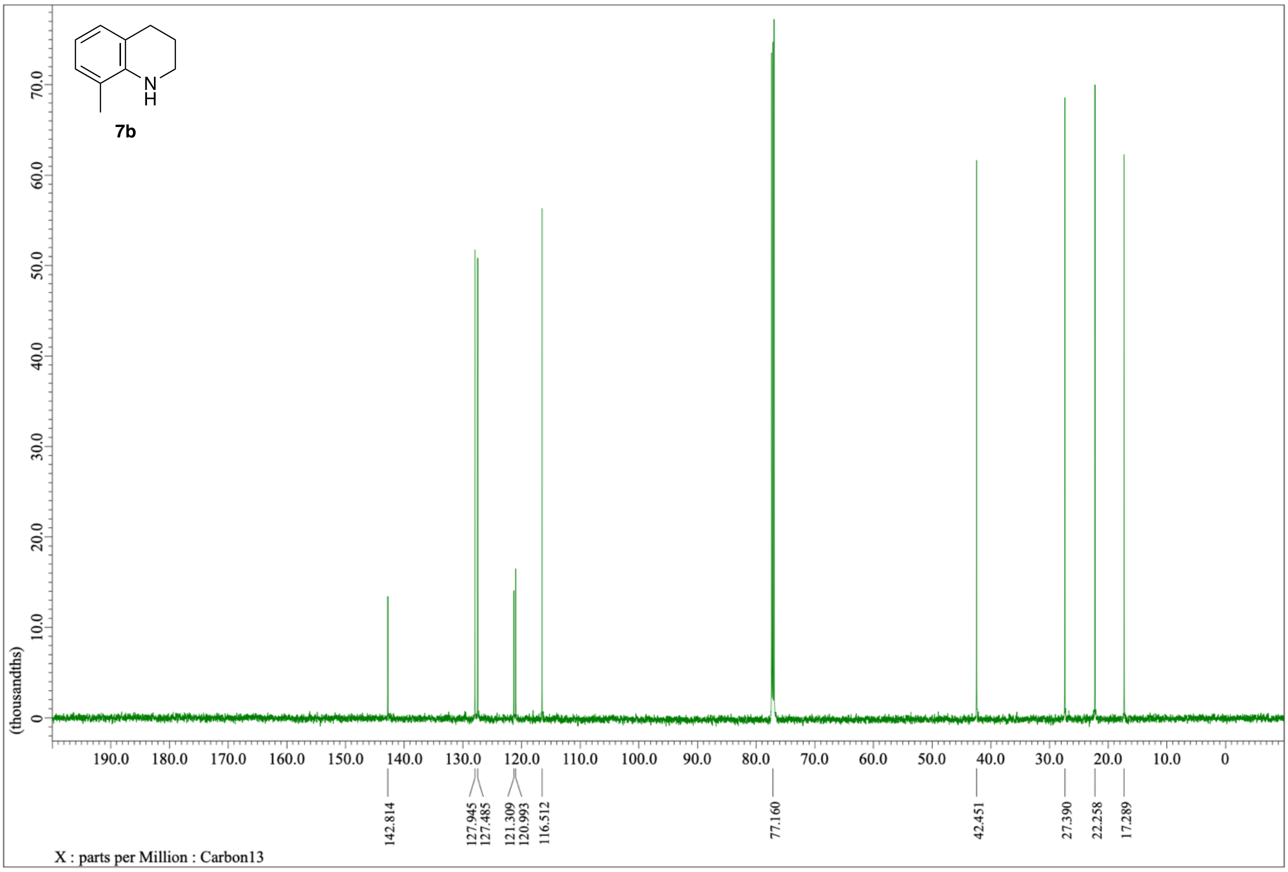

7-Methyl-1,2,3,4-tetrahydroquinoline (7c) ^{1}H NMR (600 MHz, $CDCl_3$)

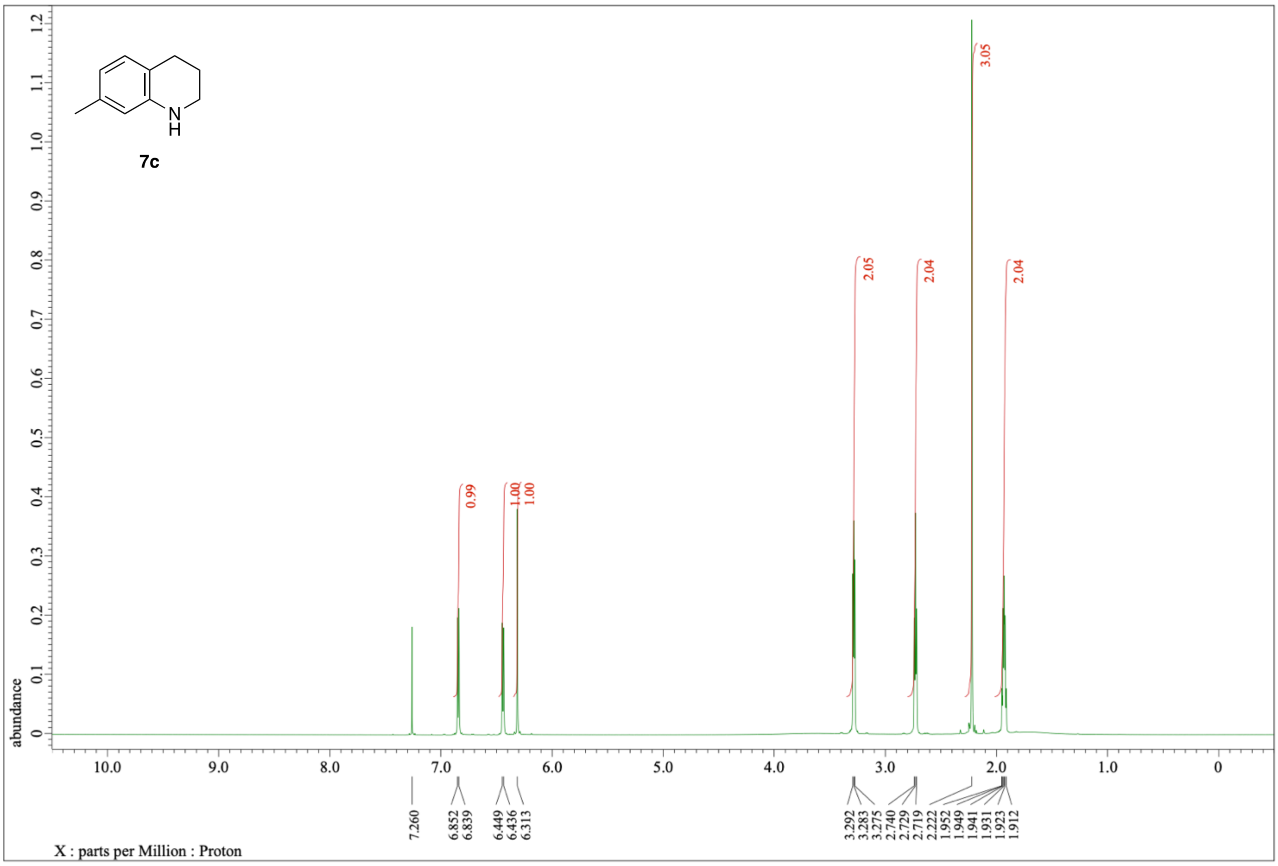

7-Methyl-1,2,3,4-tetrahydroquinoline (7c) $^{13}C\{^{1}H\}$ NMR (150 MHz, $CDCl_3$)

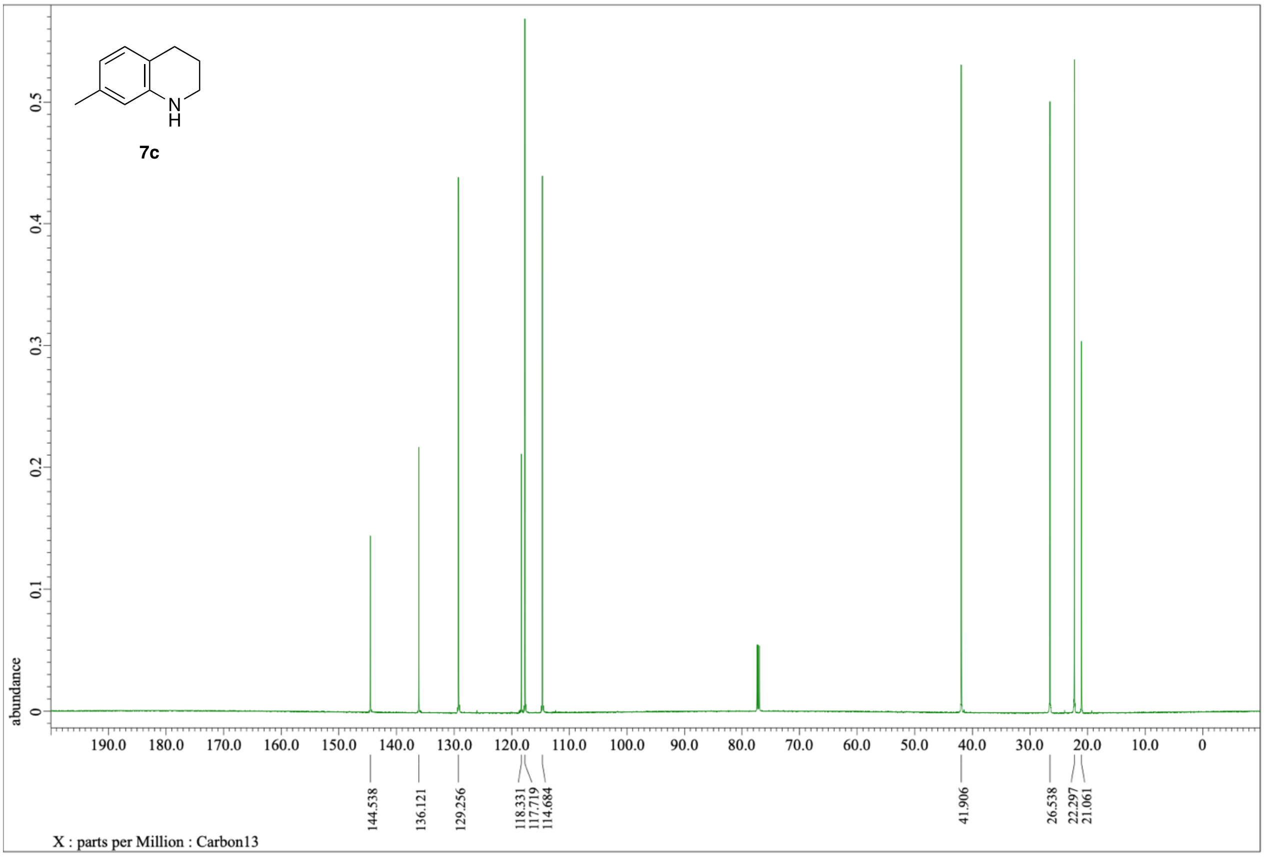

6-Methyl-1,2,3,4-tetrahydroquinoline (7d) ^{1}H NMR (600 MHz, $CDCl_3$)

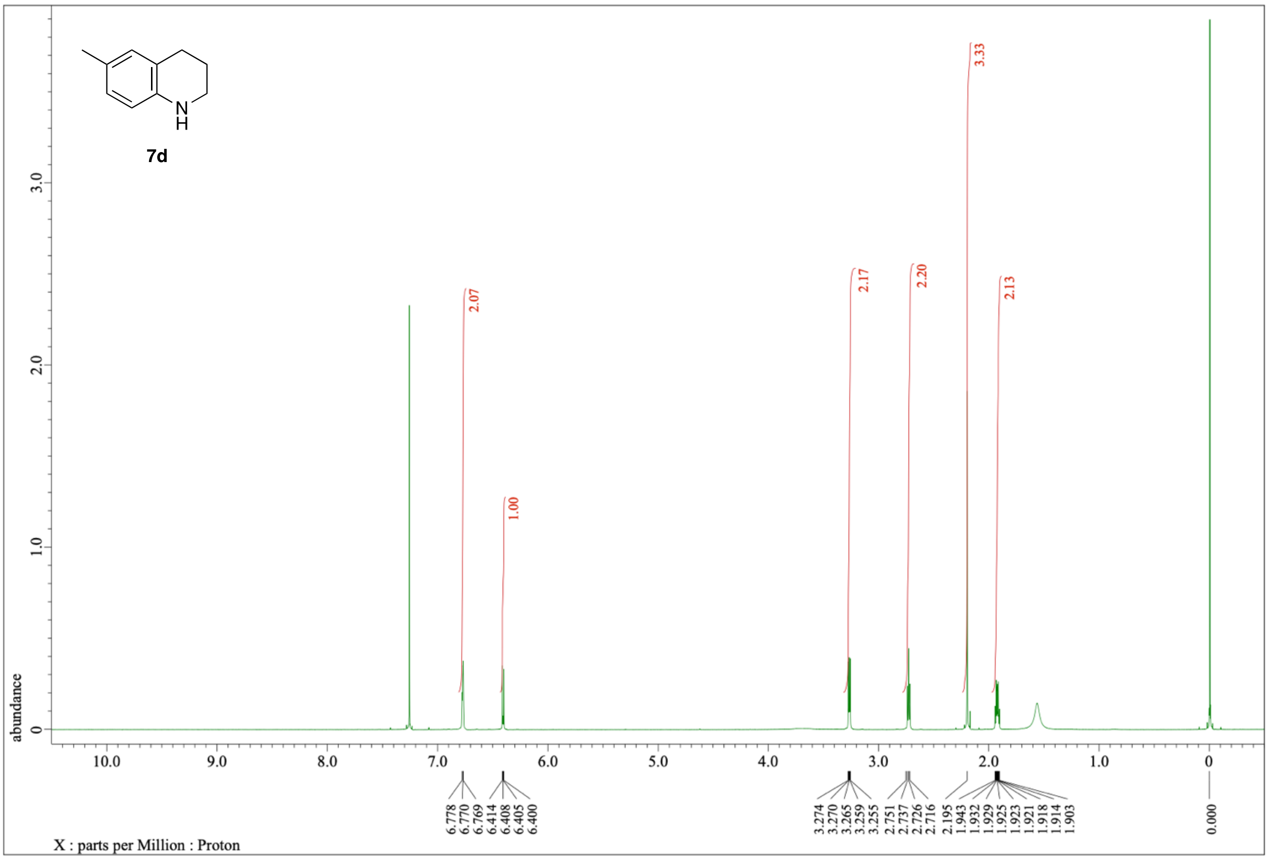

6-Methyl-1,2,3,4-tetrahydroquinoline (7d) ^{13}C{^{1}H} NMR (150 MHz, $CDCl_3$)

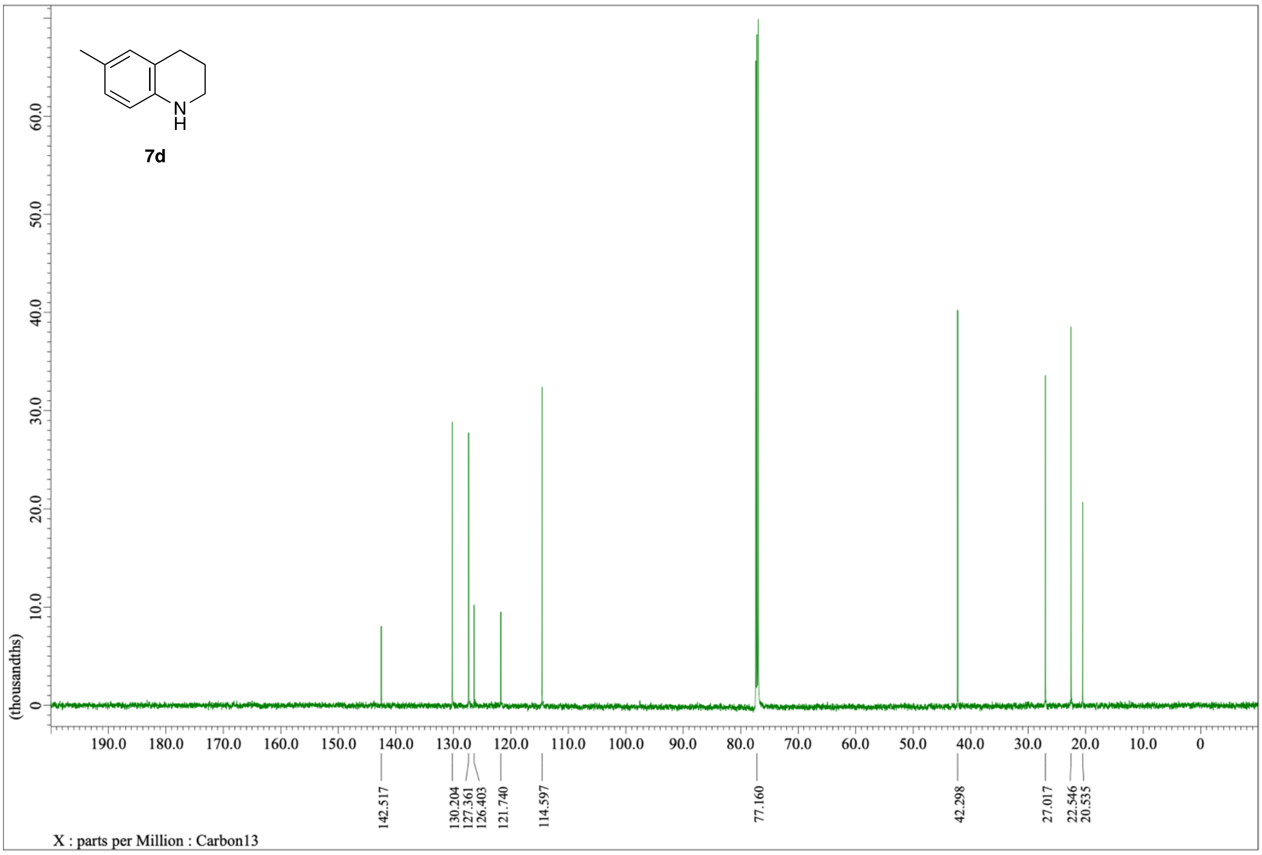

5-Methyl-1,2,3,4-tetrahydroquinoline (7e) ^{1}H NMR (600 MHz, $CDCl_3$)

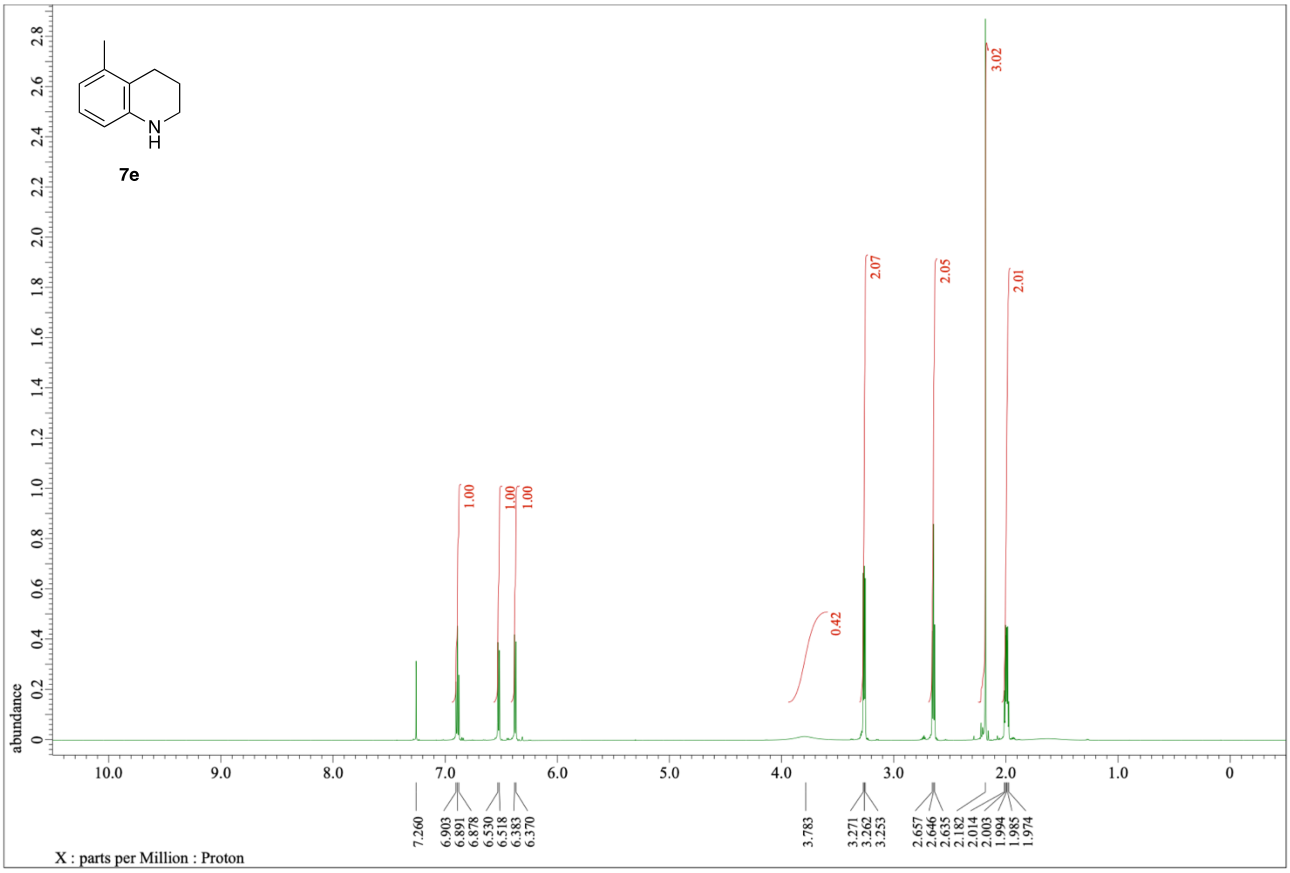

5-Methyl-1,2,3,4-tetrahydroquinoline (7e) ^{13}C{^{1}H} NMR (150 MHz, $CDCl_3$)

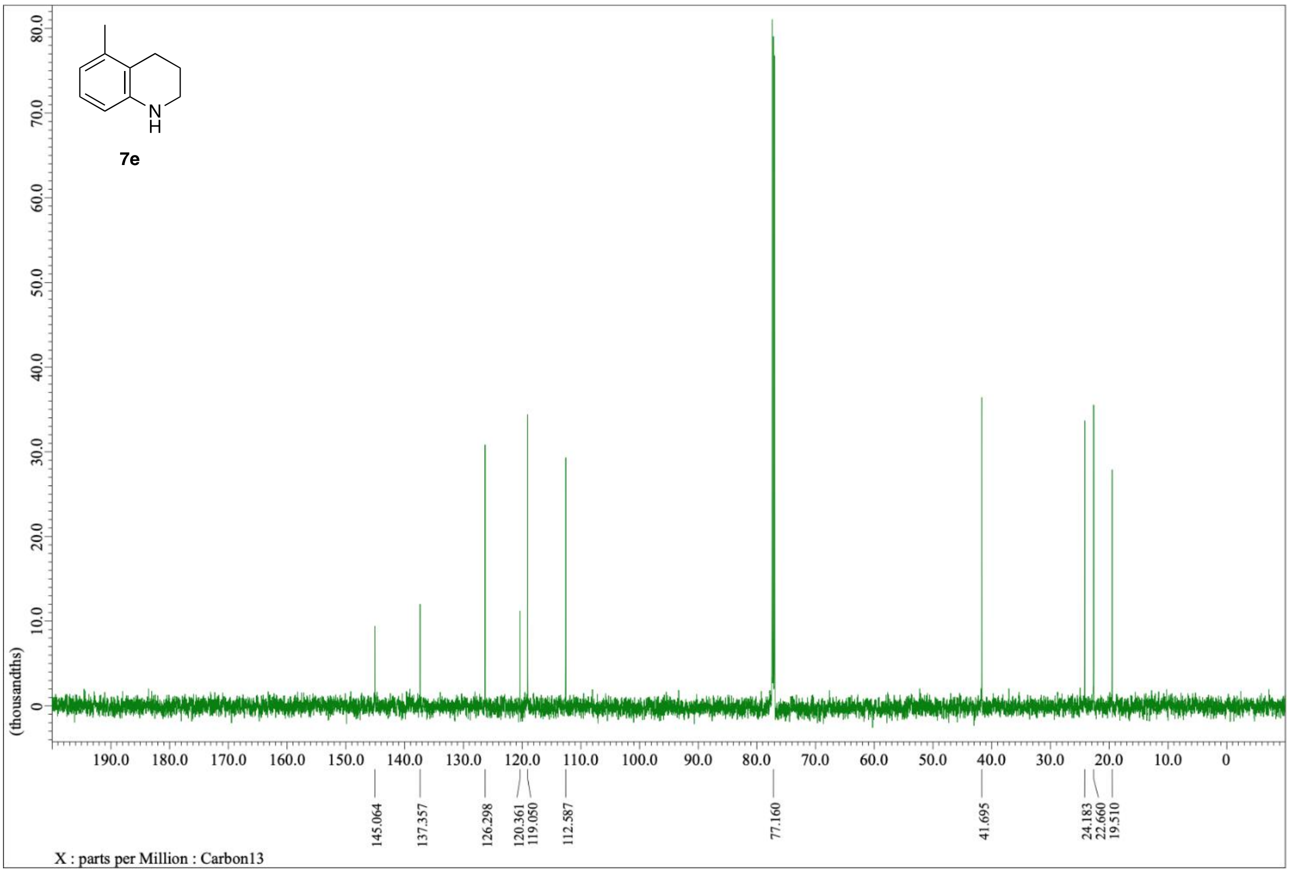

3-Methyl-1,2,3,4-tetrahydroquinoline (7g) ^{1}H NMR (600 MHz, CDCl$_3$)

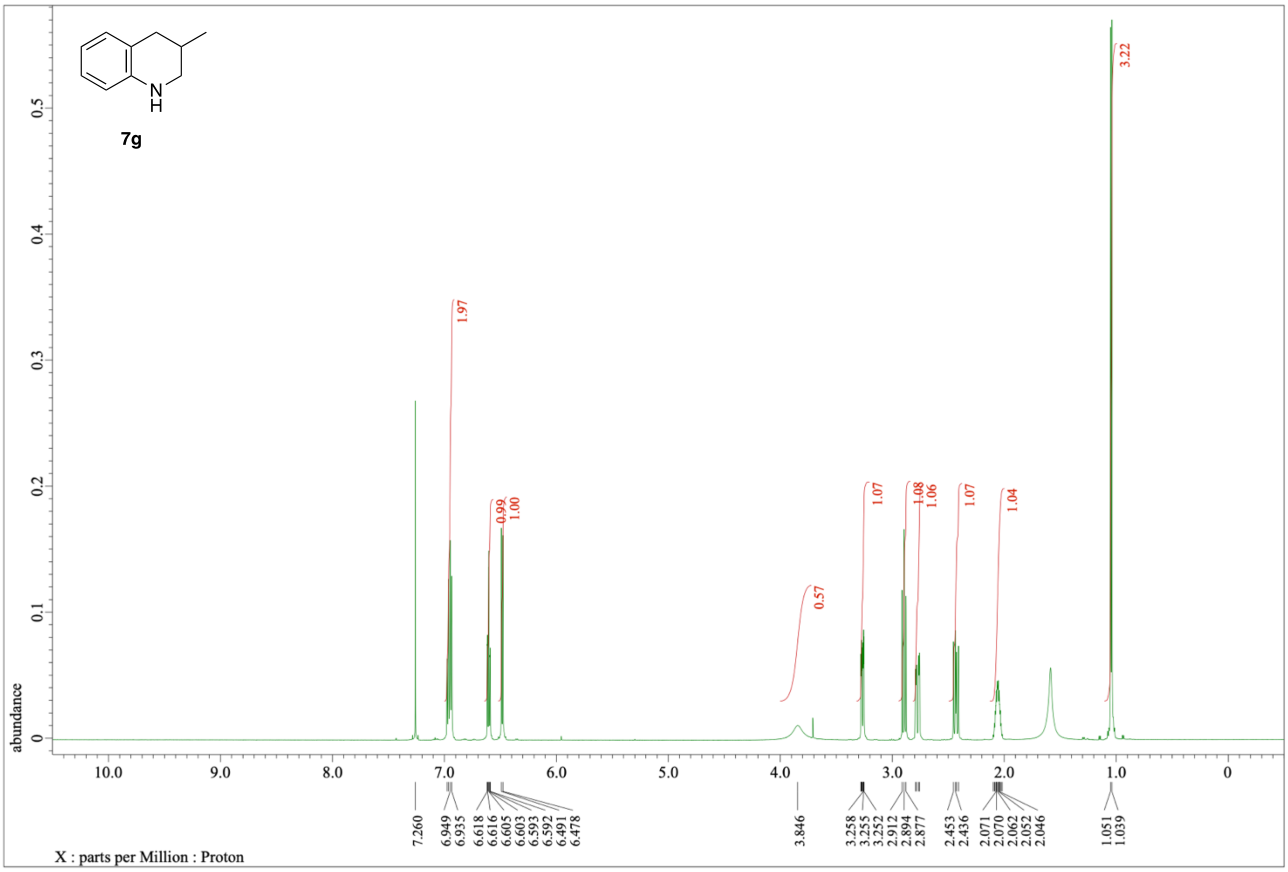

3-Methyl-1,2,3,4-tetrahydroquinoline (7g) ^{13}C{^{1}H} NMR (150 MHz, CDCl$_3$)

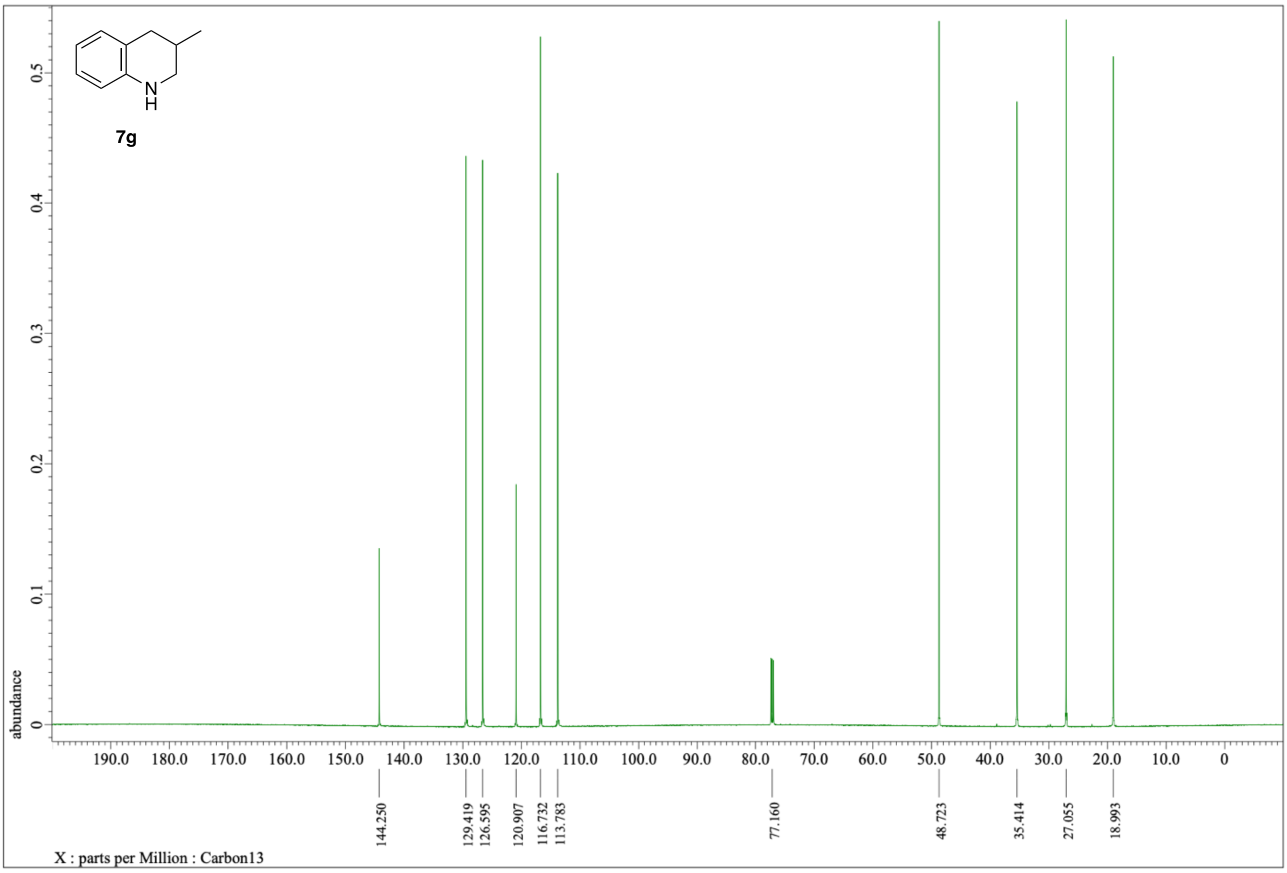

2-Methyl-1,2,3,4-tetrahydroquinoline (7h) ^{1}H NMR (600 MHz, $CDCl_3$)

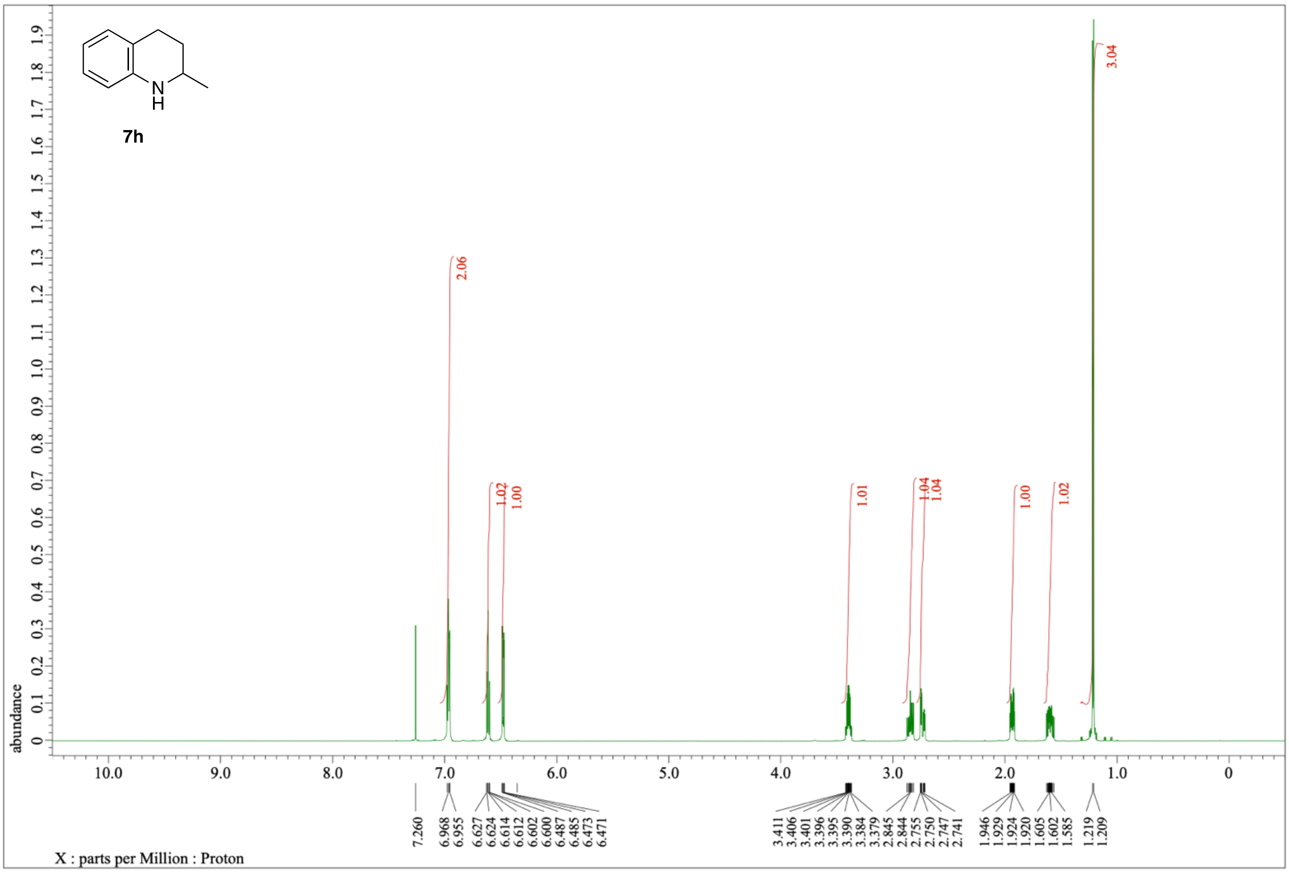

2-Methyl-1,2,3,4-tetrahydroquinoline (7h) $^{13}C\{^{1}H\}$ NMR (150 MHz, $CDCl_3$)

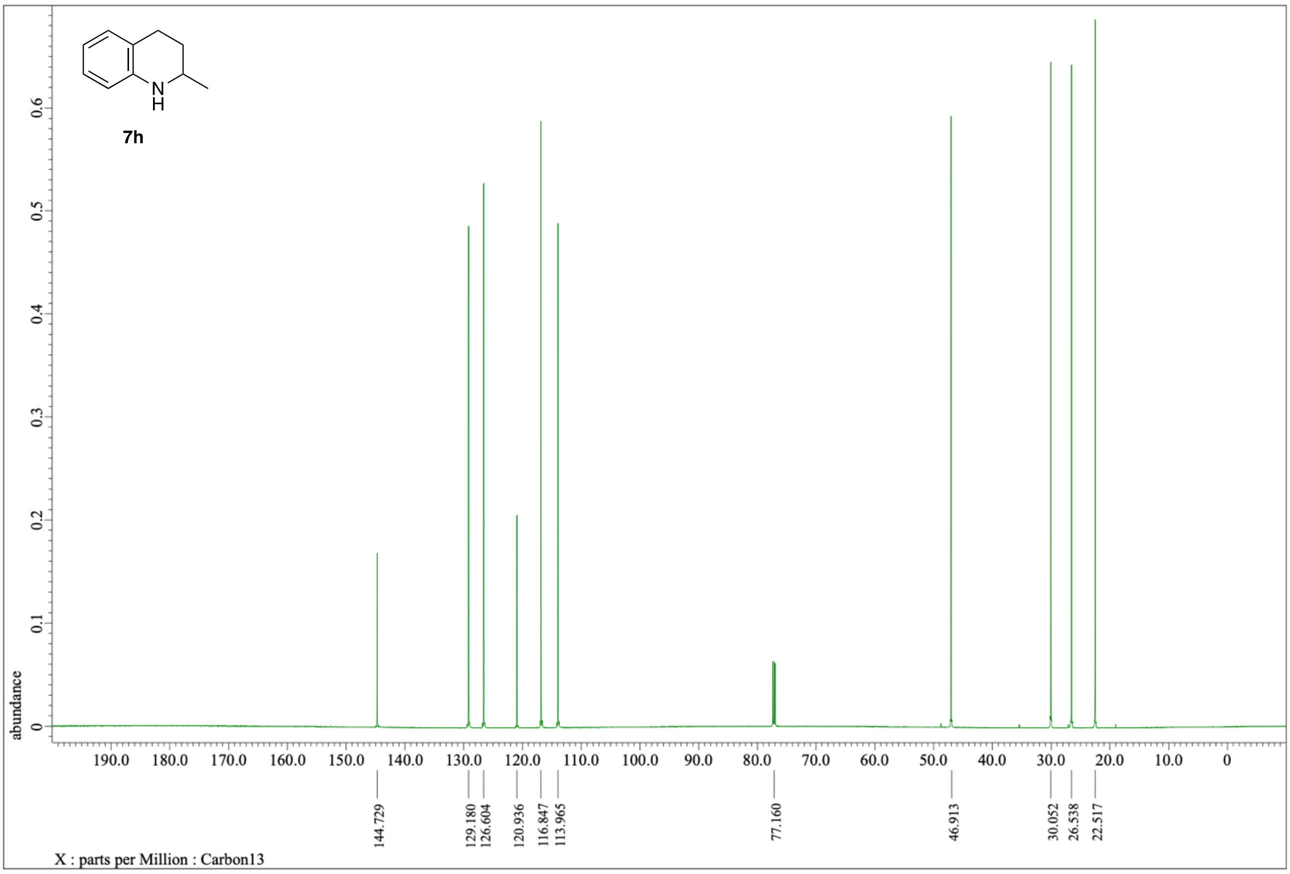

2,3-Dimethyl-1,2,3,4-tetrahydroquinoline (7i) ^{1}H NMR (600 MHz, $CDCl_3$)

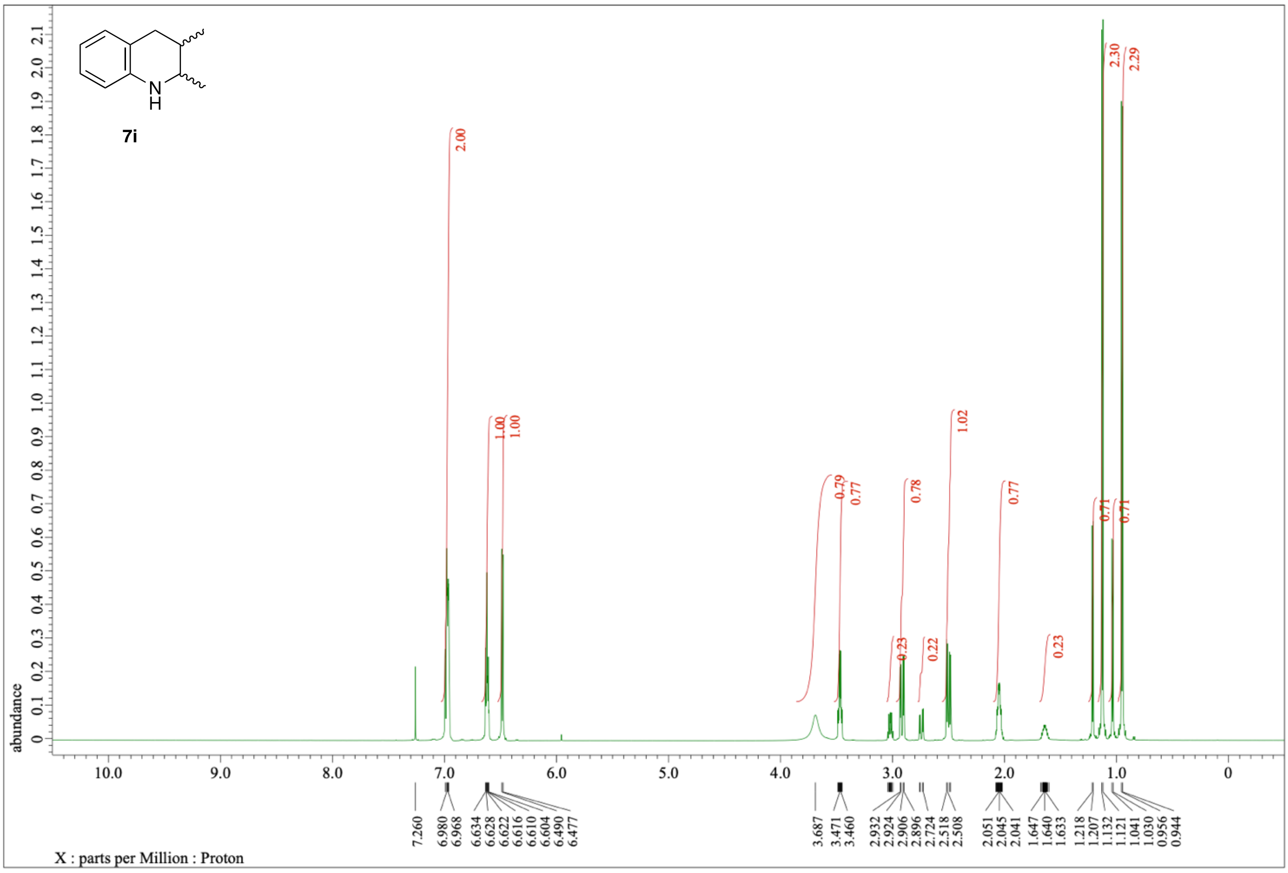

2,3-Dimethyl-1,2,3,4-tetrahydroquinoline (7i) ^{13}C {^{1}H} NMR (150 MHz, $CDCl_3$)

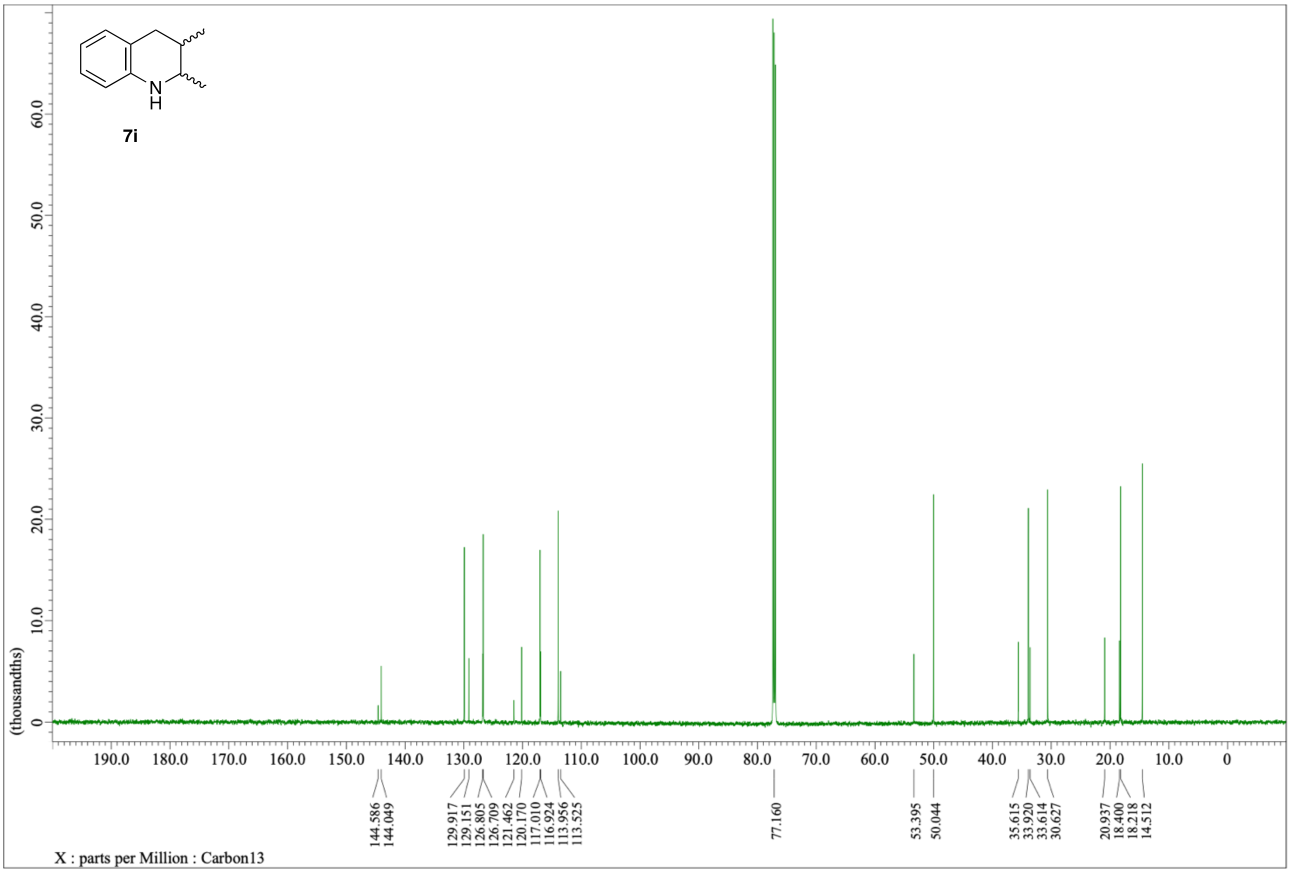

2-Phenyl-1,2,3,4-tetrahydroquinoline (7j) ^{1}H NMR (600 MHz, $CDCl_3$)

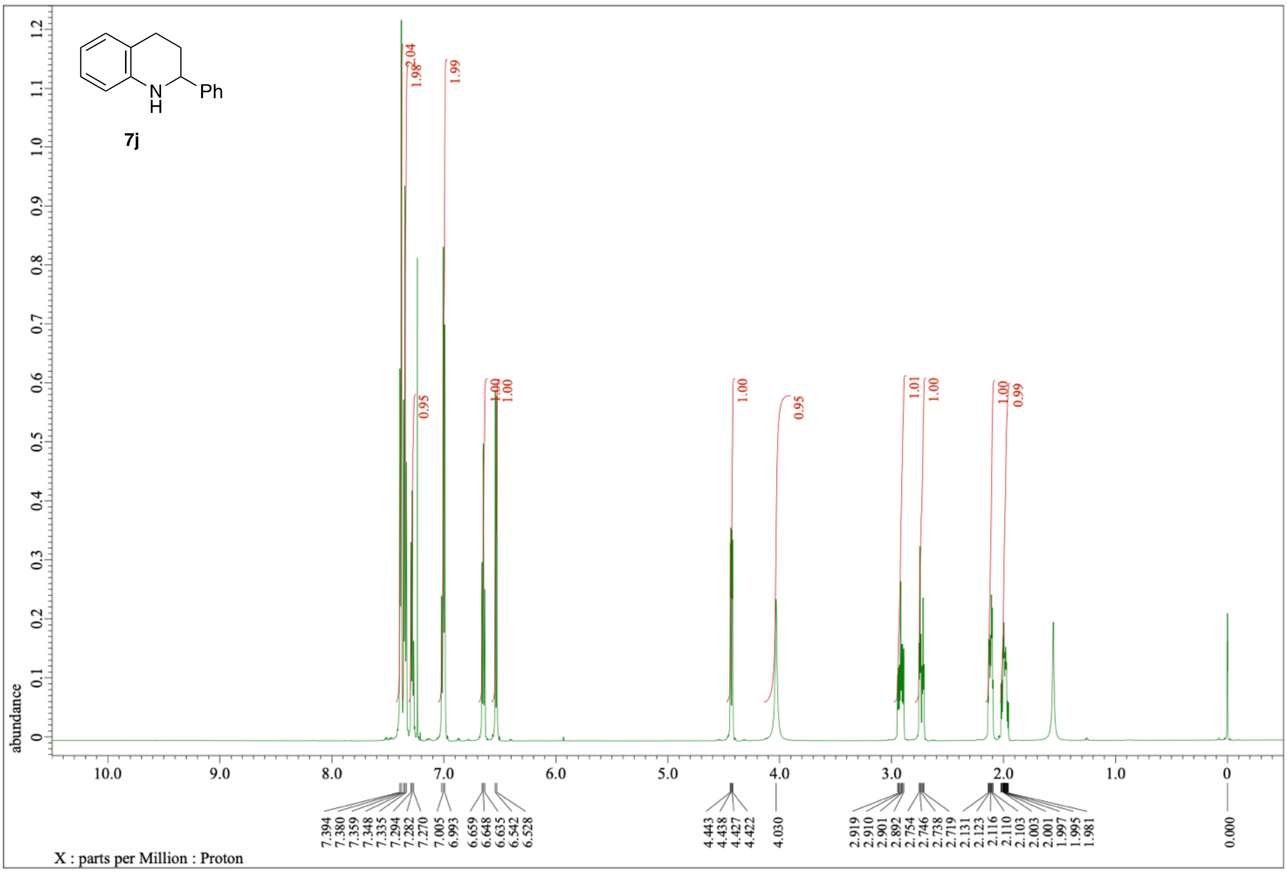

2-Phenyl-1,2,3,4-tetrahydroquinoline (7j) ^{13}C{^{1}H} NMR (150 MHz, $CDCl_3$)

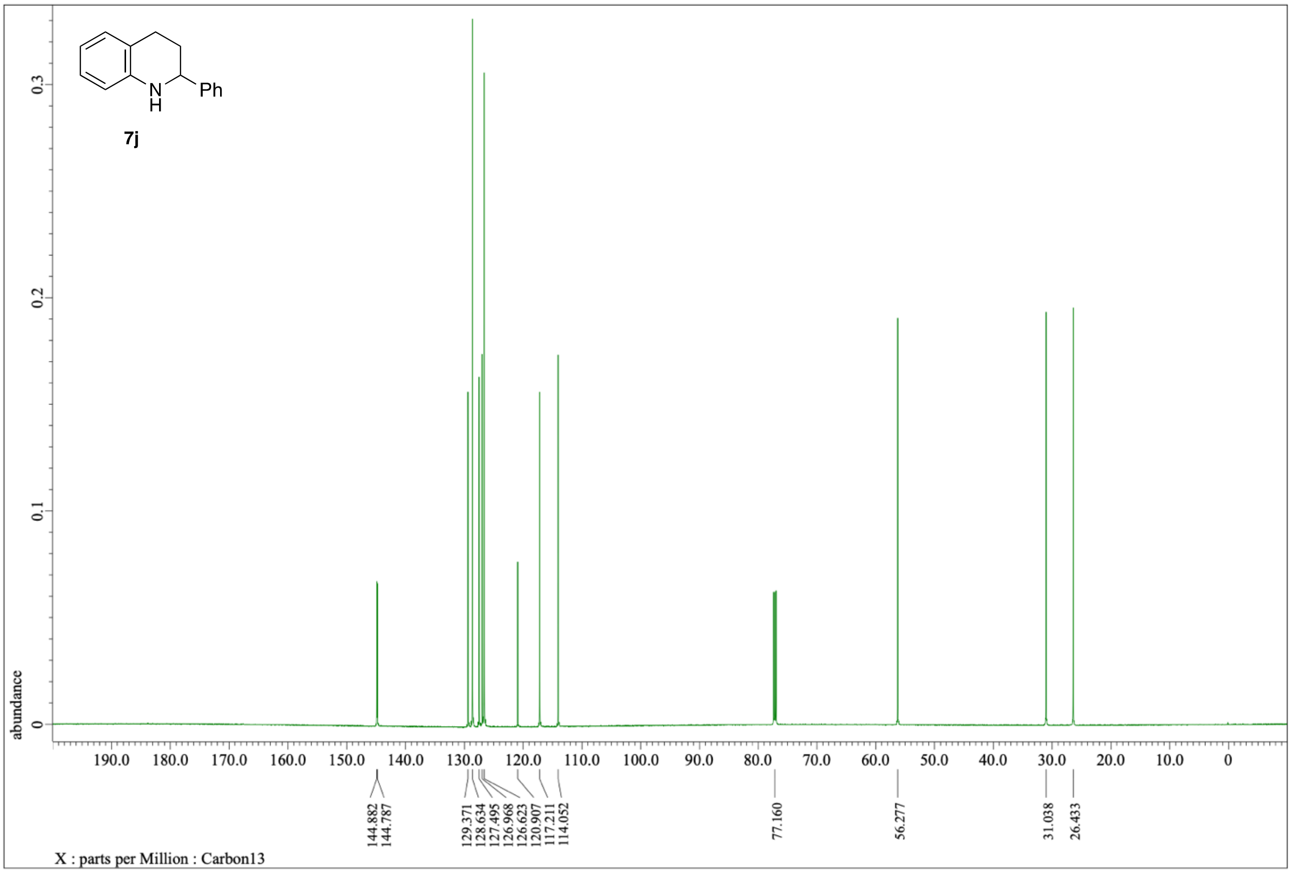

6-Acetyl-1,2,3,4-tetrahydroquinoline (7k) ^{1}H NMR (600 MHz, $CDCl_3$)

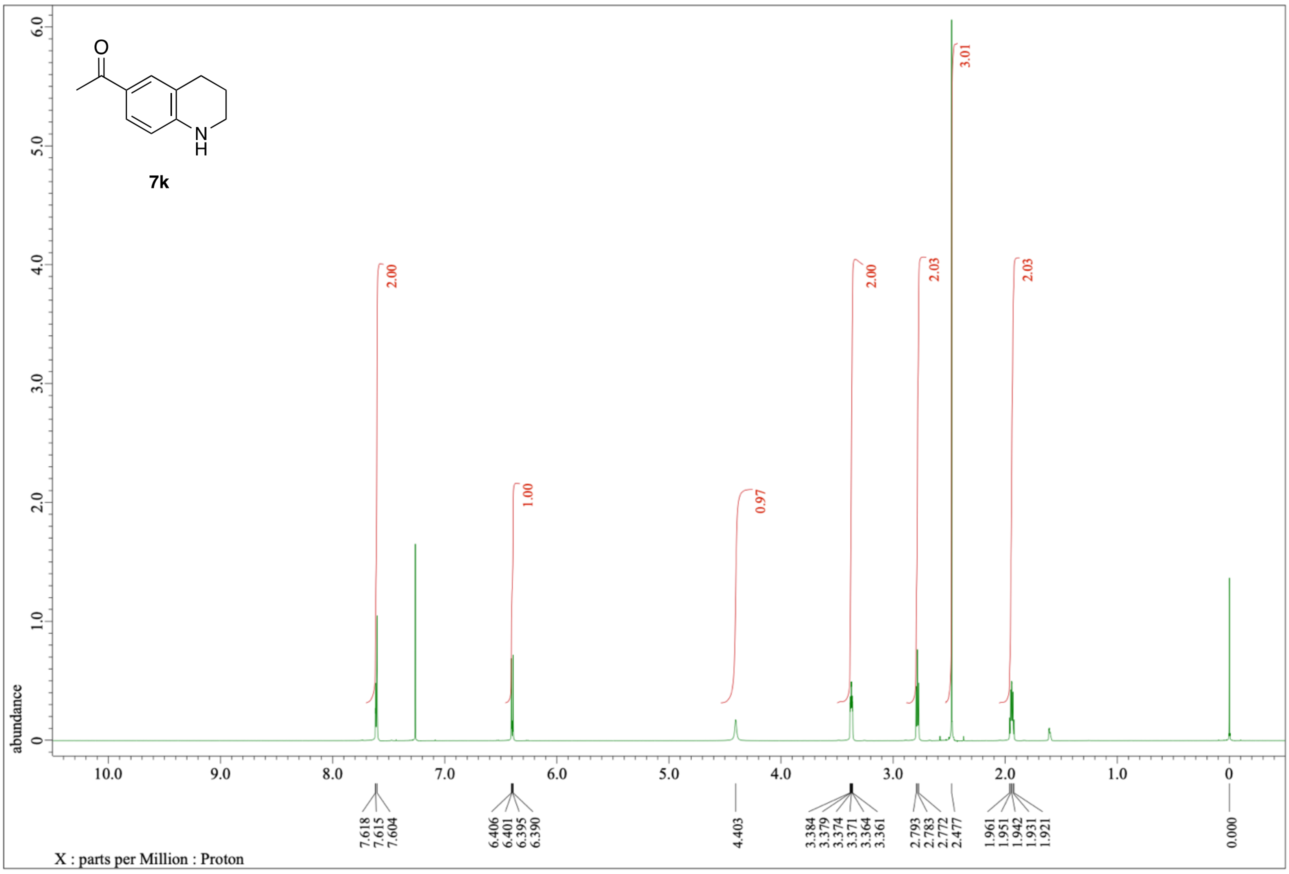

6-Acetyl-1,2,3,4-tetrahydroquinoline (7k) ^{13}C {^{1}H} NMR (150 MHz, $CDCl_3$)

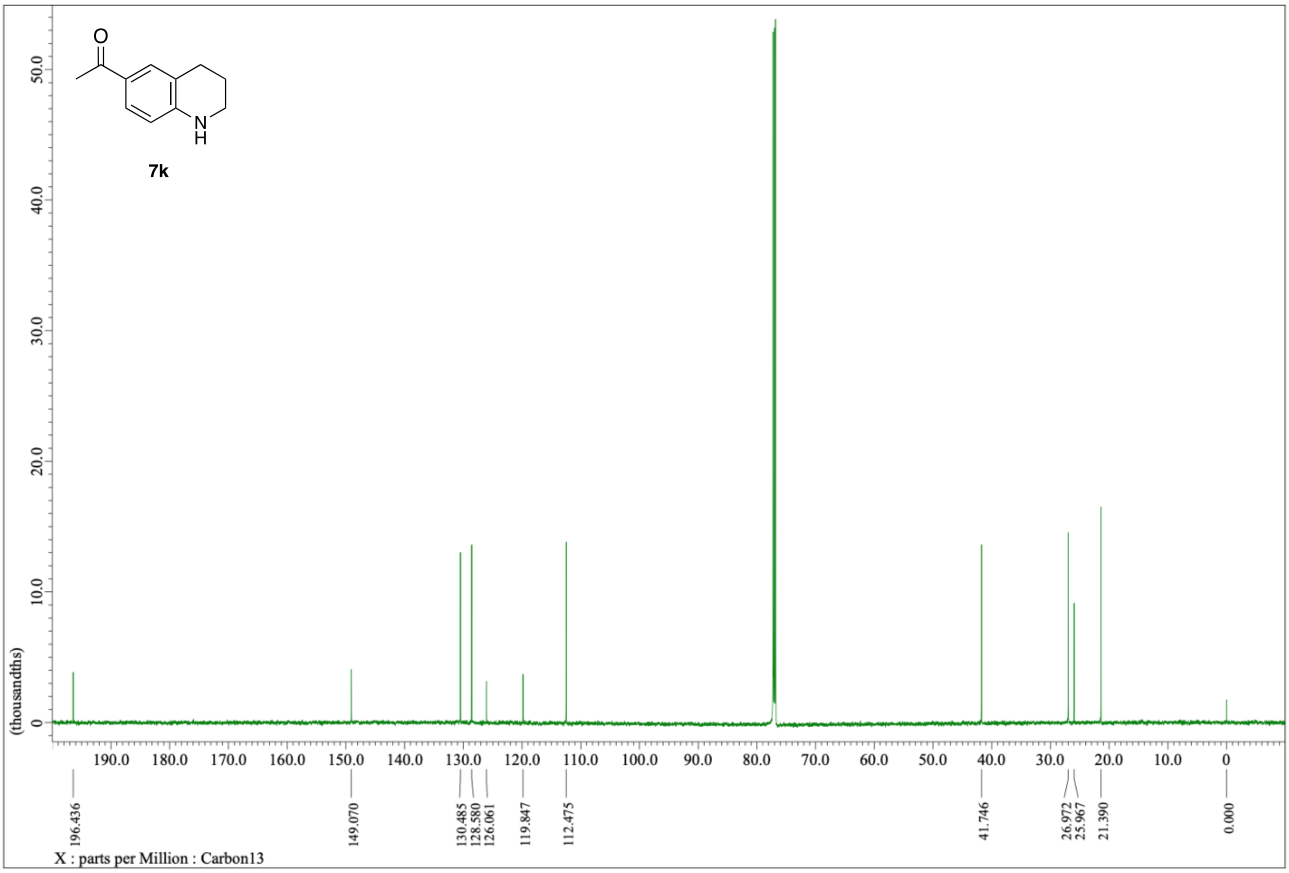

Methyl 1,2,3,4-tetrahydroquinoline-6-carboxylate (7l) ^{1}H NMR (600 MHz, CDCl$_3$)

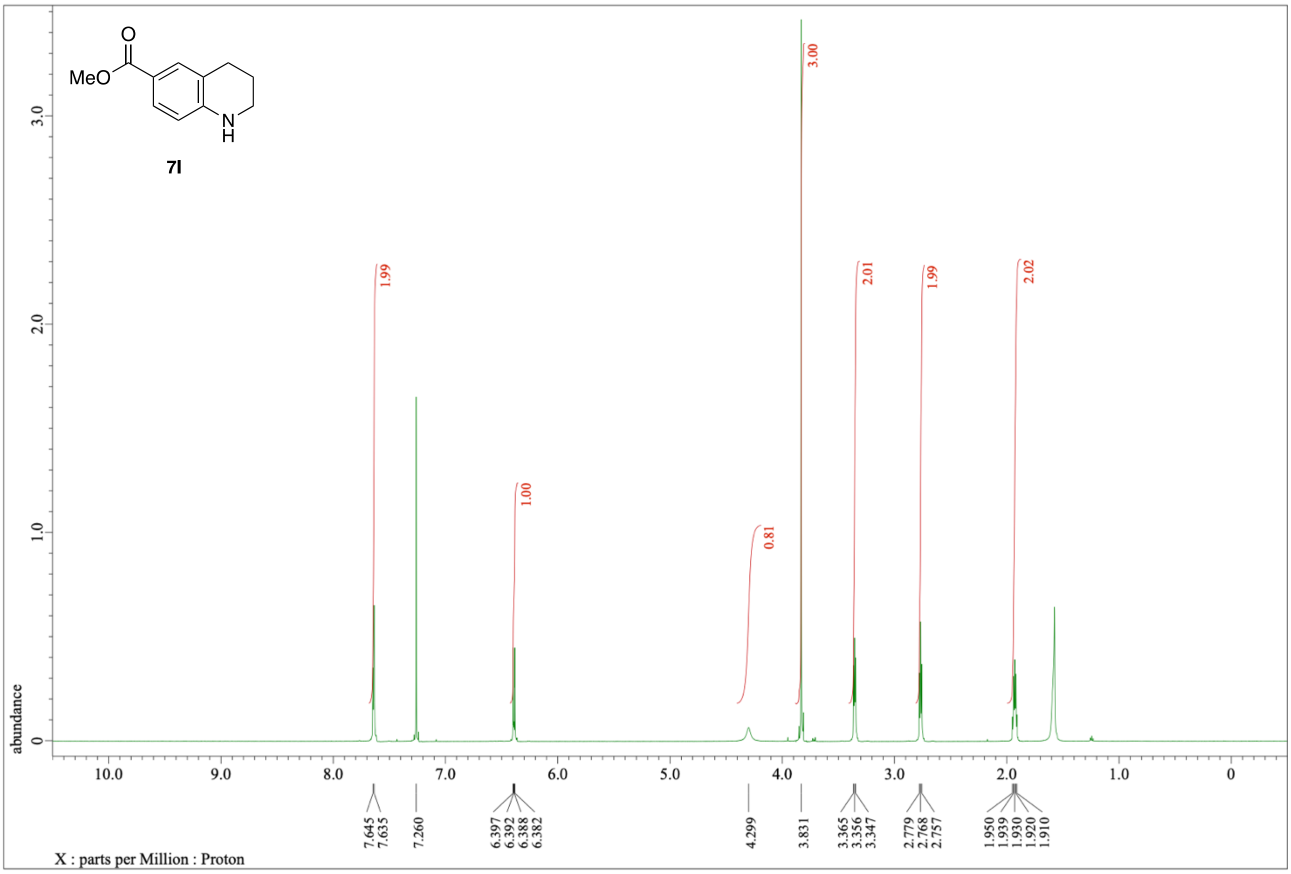

Methyl 1,2,3,4-tetrahydroquinoline-6-carboxylate (7l) ^{13}C {^{1}H} NMR (150 MHz, CDCl$_3$)

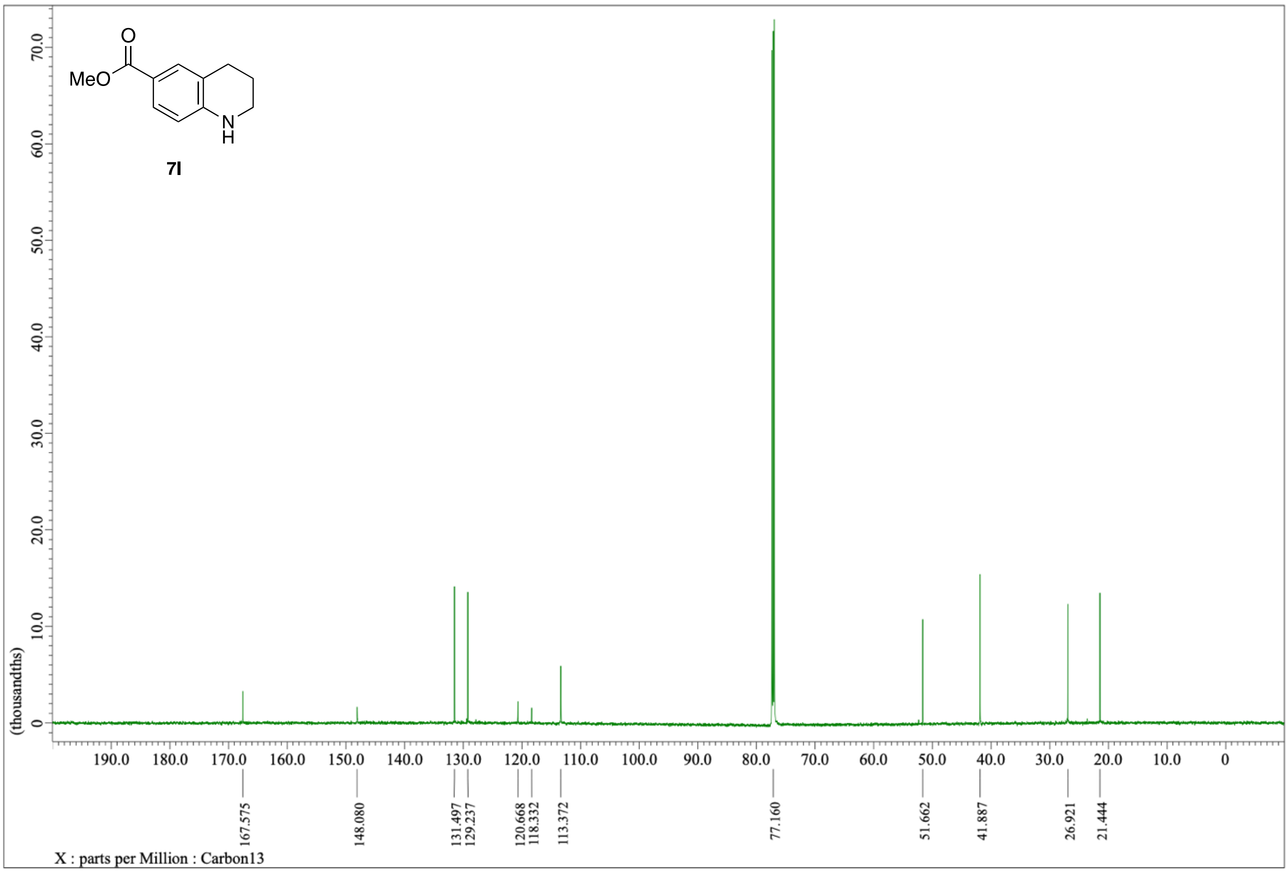

1,2,3,4-Tetrahydroquinoline-6-carboxamide (7m) ^{1}H NMR (600 MHz, CDCl$_3$)

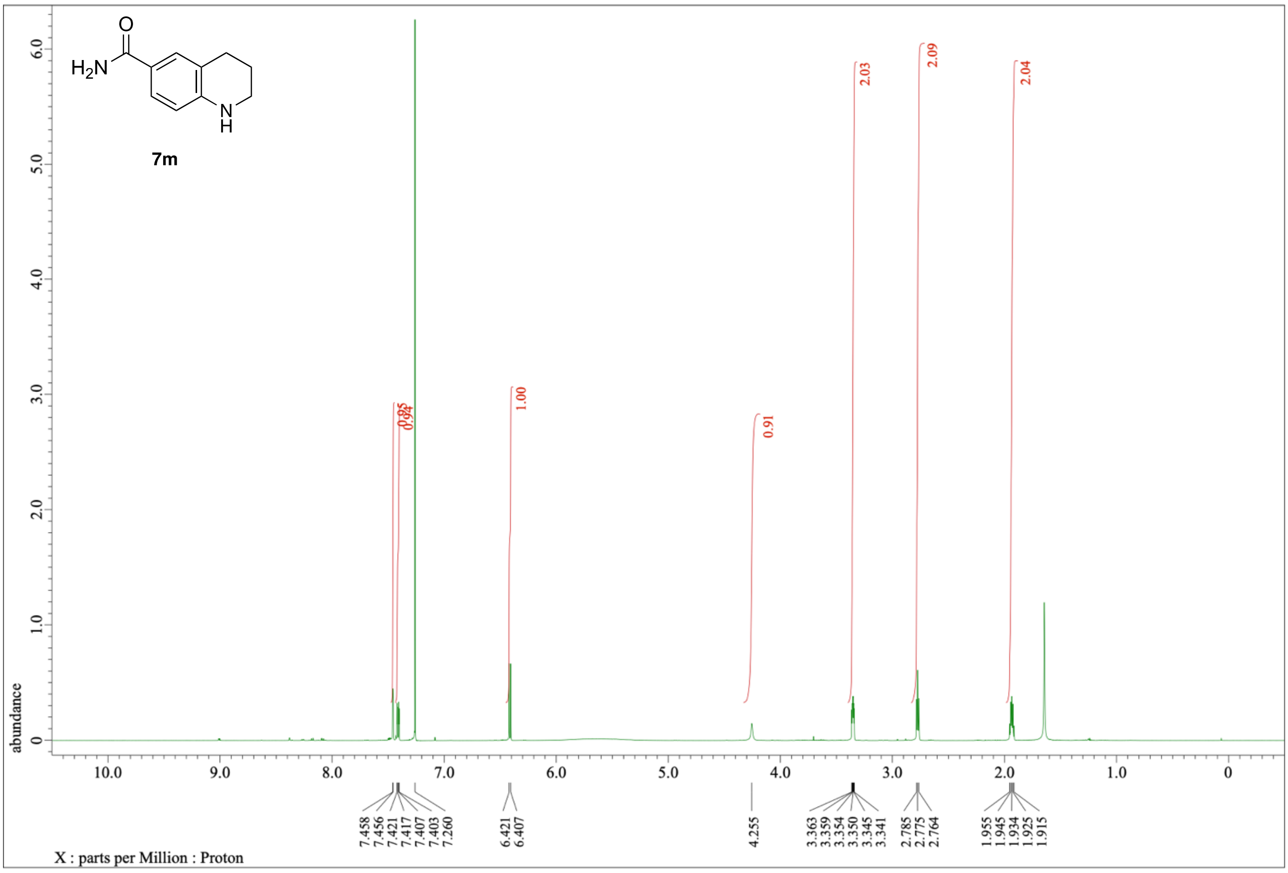

1,2,3,4-Tetrahydroquinoline-6-carboxamide (7m) ^{13}C {^{1}H} NMR (150 MHz, CDCl$_3$)

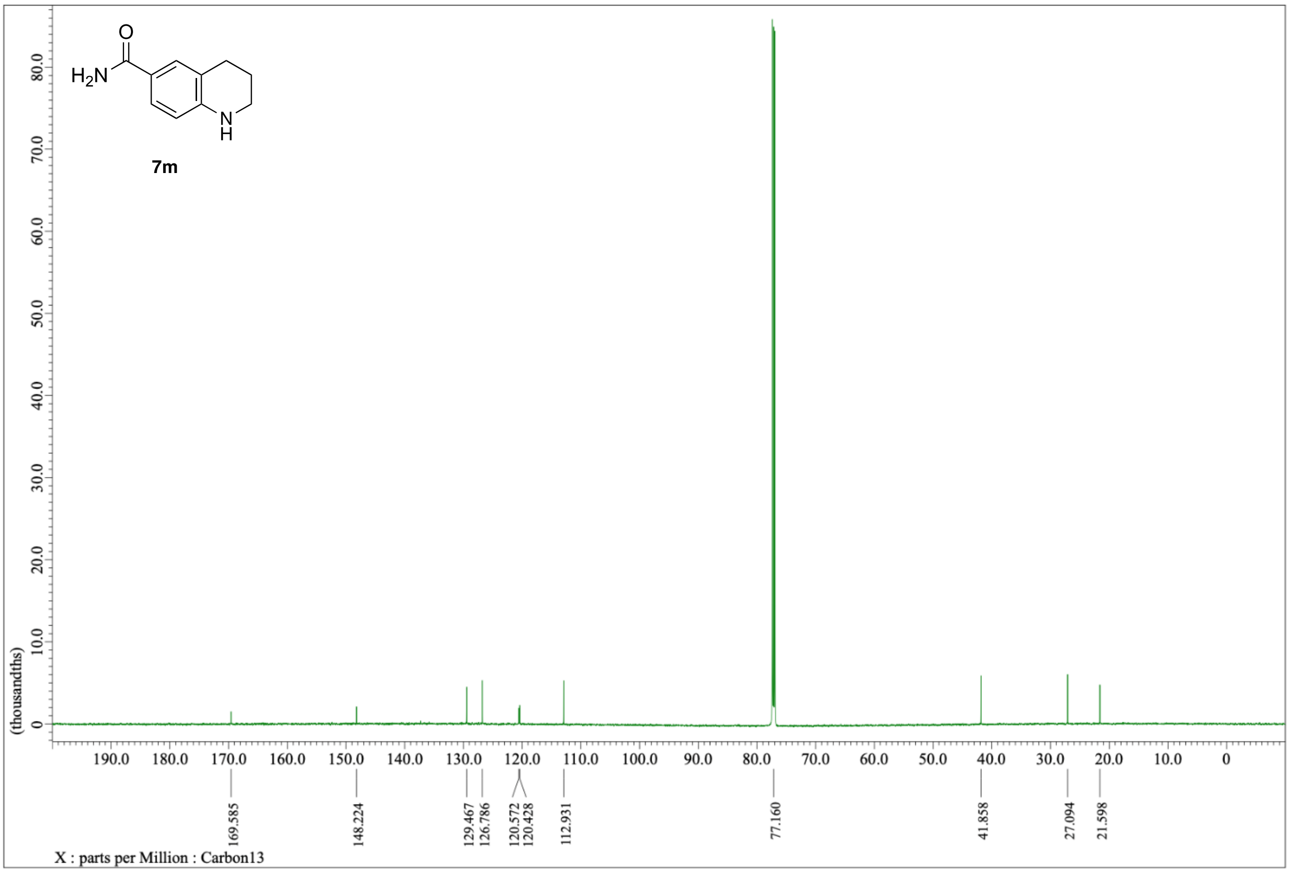

6-(Trifluoromethyl)-1,2,3,4-tetrahydroquinoline (7n) ^{1}H NMR (600 MHz, CDCl$_3$)

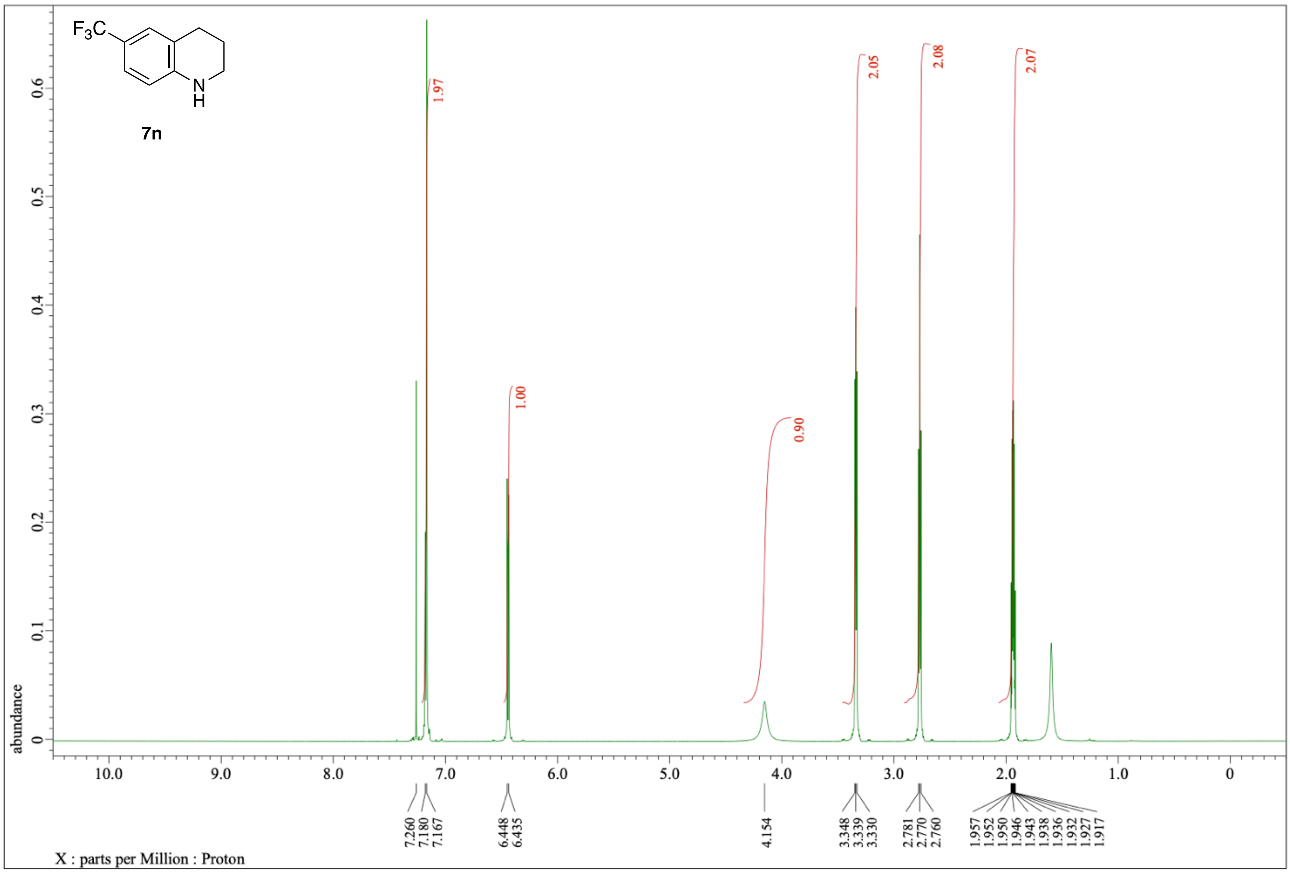

6-(Trifluoromethyl)-1,2,3,4-tetrahydroquinoline (7n) ^{13}C {^{1}H} NMR (150 MHz, CDCl$_3$)

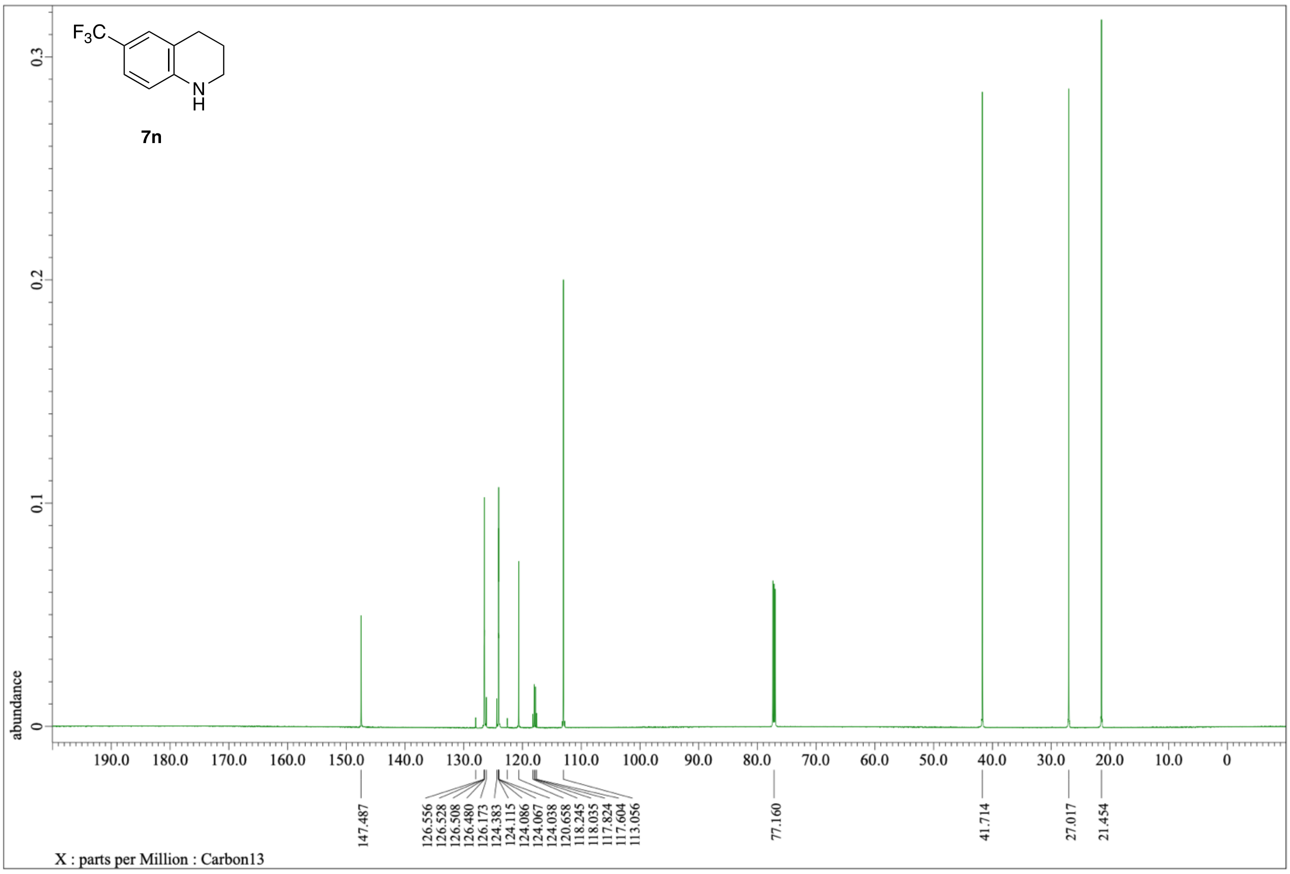

6-(Trifluoromethyl)quinoline (7n) ^{19}F NMR (565 MHz, CDCl$_3$)

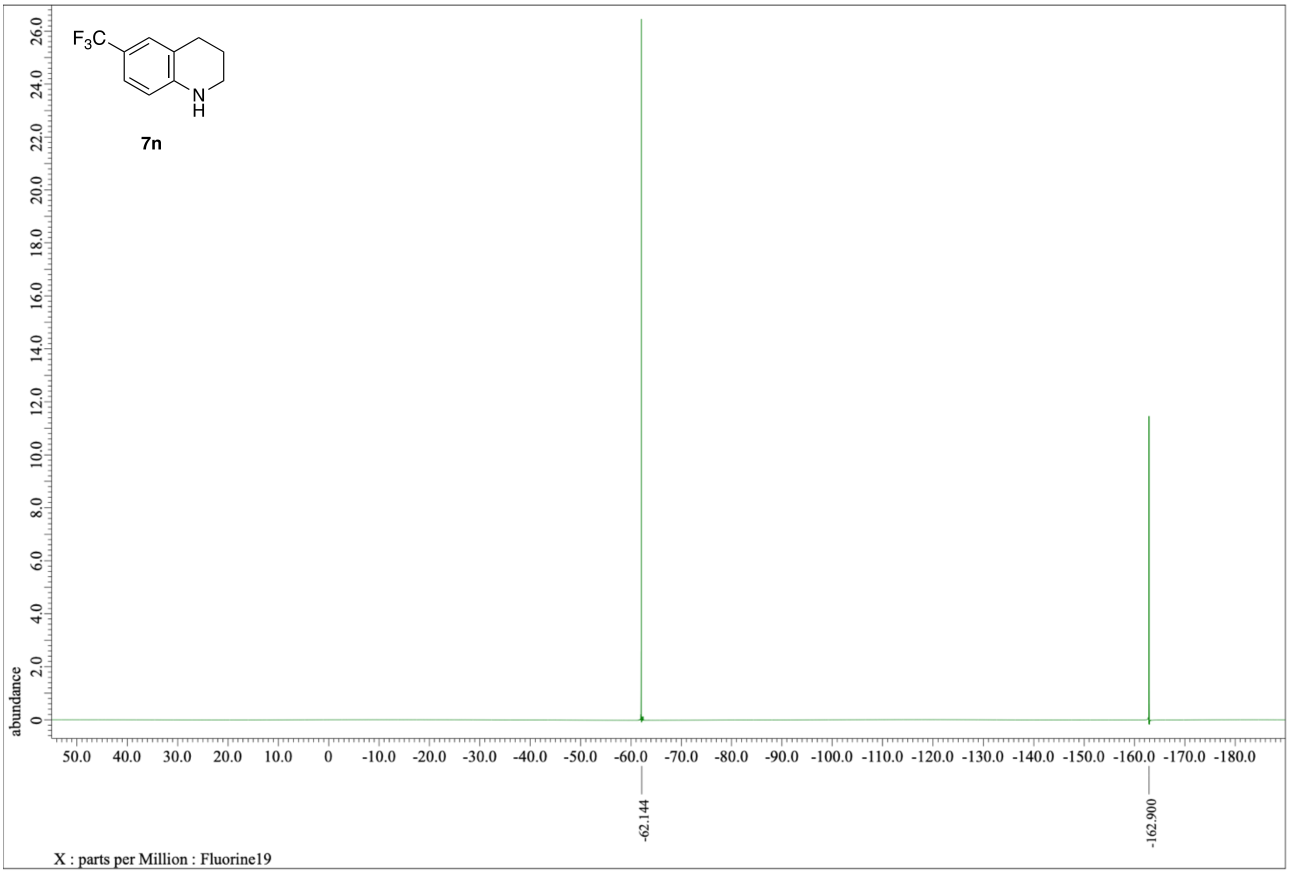

8-Methoxy-1,2,3,4-tetrahydroquinoline (7o) ^{1}H NMR (600 MHz, $CDCl_3$)

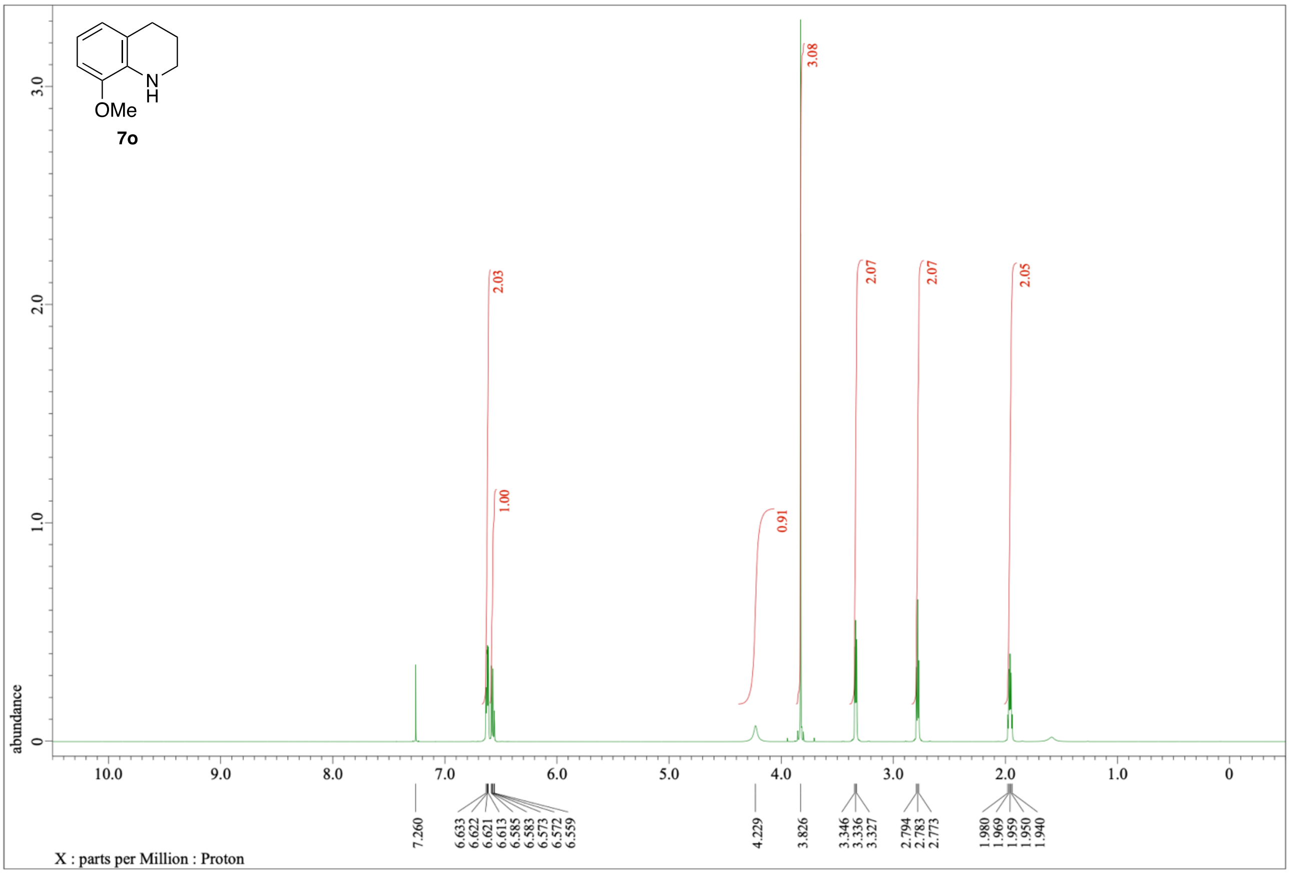

8-Methoxy-1,2,3,4-tetrahydroquinoline (7o) ^{13}C{^{1}H} NMR (150 MHz, $CDCl_3$)

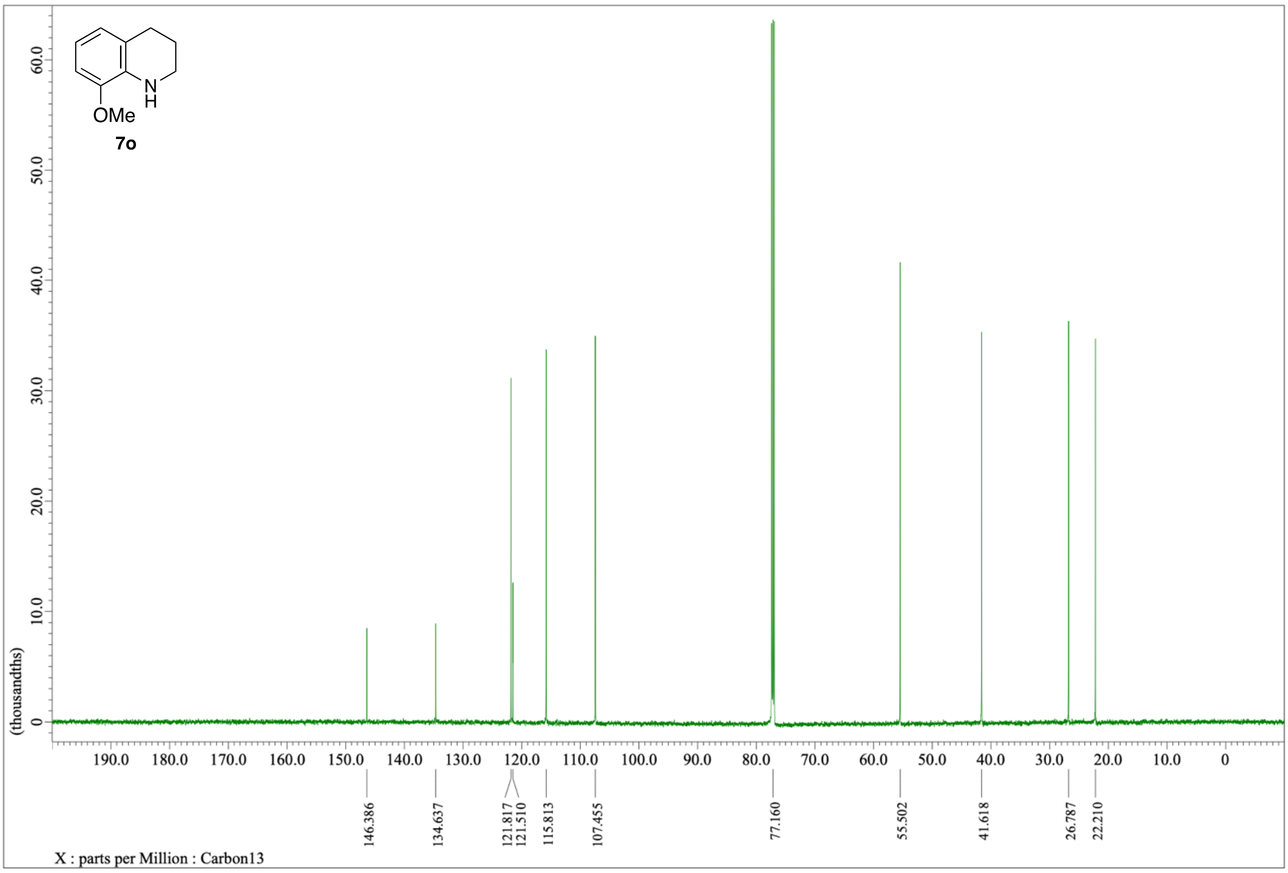

6-Methoxy-1,2,3,4-tetrahydroquinoline (7p) ^{1}H NMR (600 MHz, $CDCl_3$)

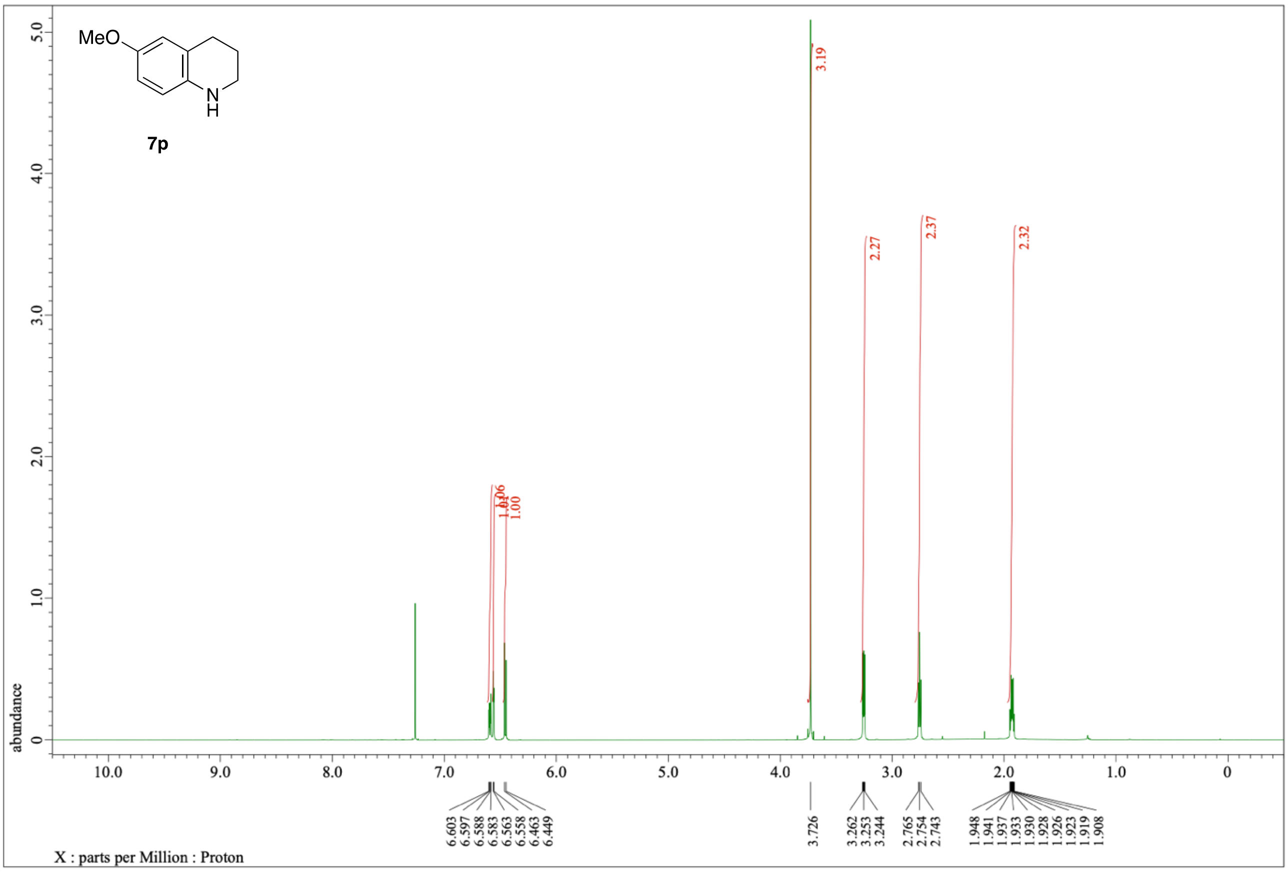

6-Methoxy-1,2,3,4-tetrahydroquinoline (7p) $^{13}C\{^1H\}$ NMR (150 MHz, $CDCl_3$)

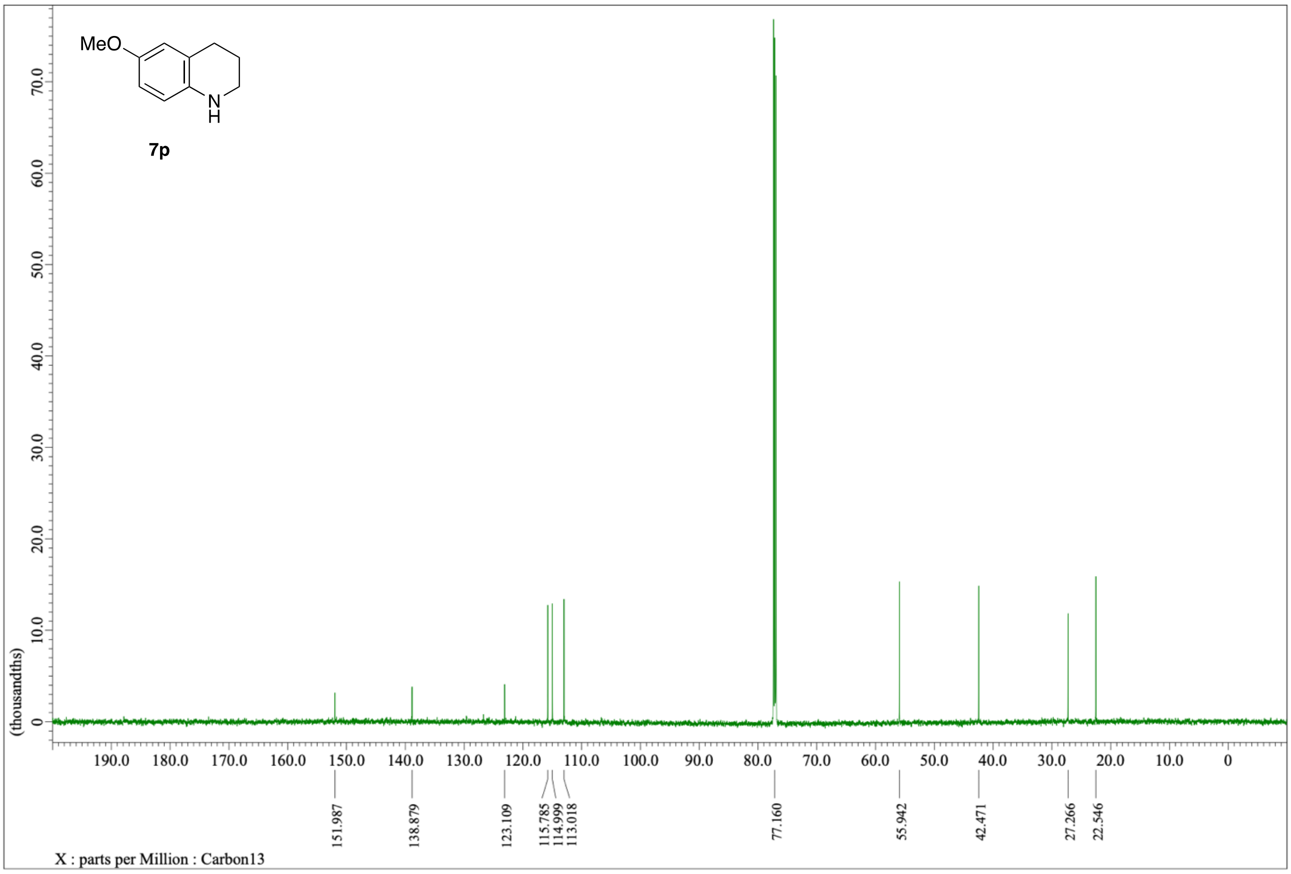

3-Methoxy-1,2,3,4-tetrahydroquinoline (7q) ^{1}H NMR (600 MHz, $CDCl_3$)

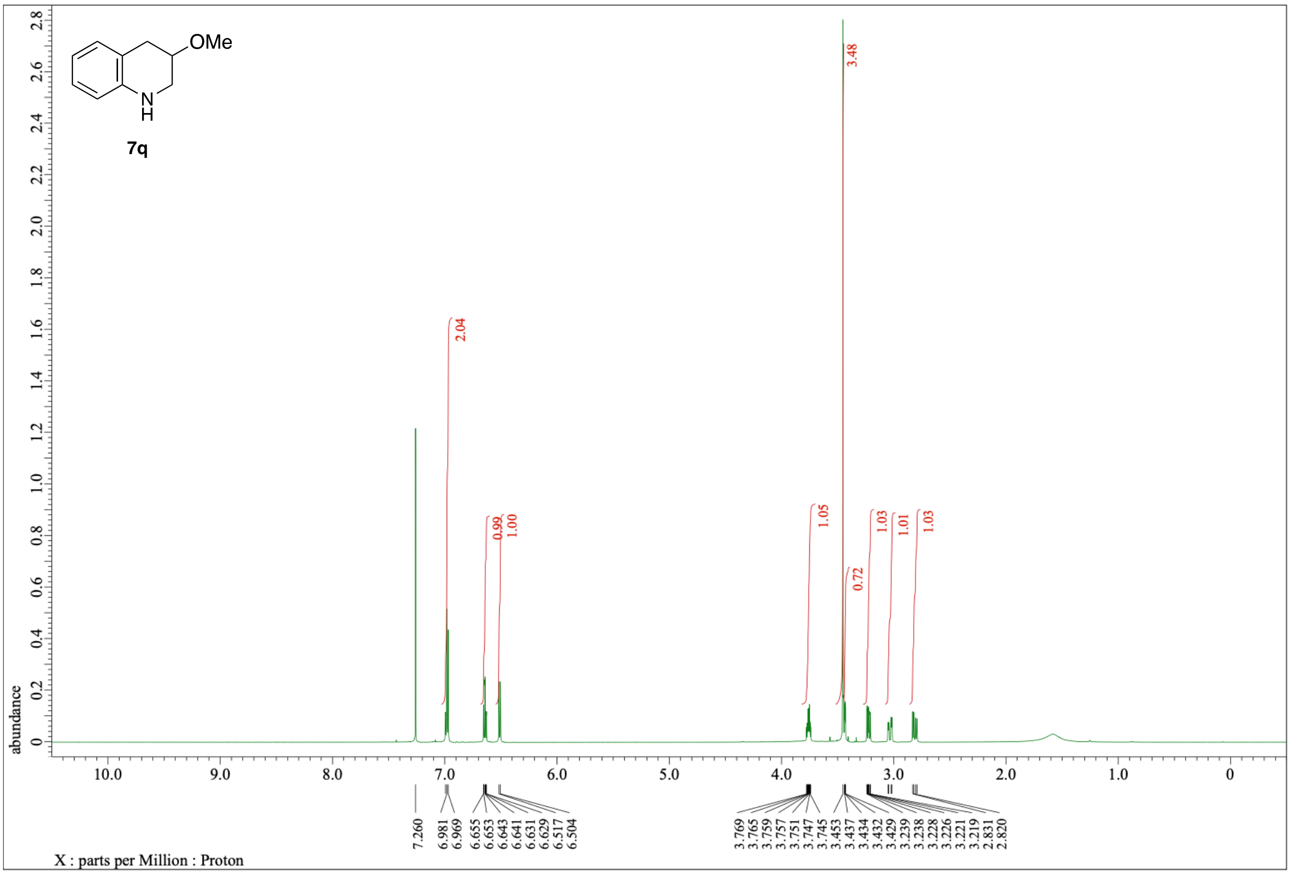

3-Methoxy-1,2,3,4-tetrahydroquinoline (7q) $^{13}C\{^{1}H\}$ NMR (150 MHz, $CDCl_3$)

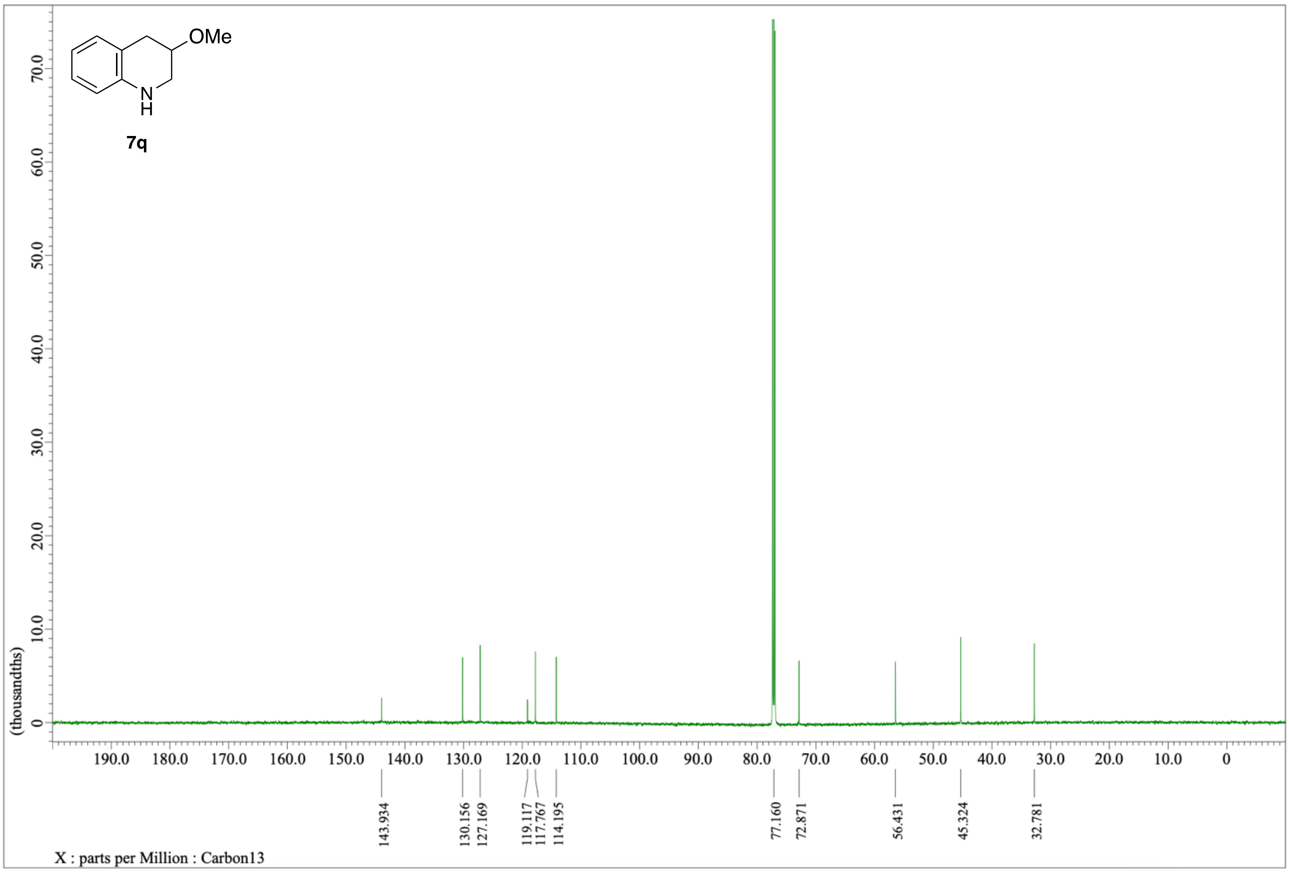

4-Methoxy-5,6,7,8-tetrahydroquinoline (7r′) ^{1}H NMR (600 MHz, $CDCl_3$)

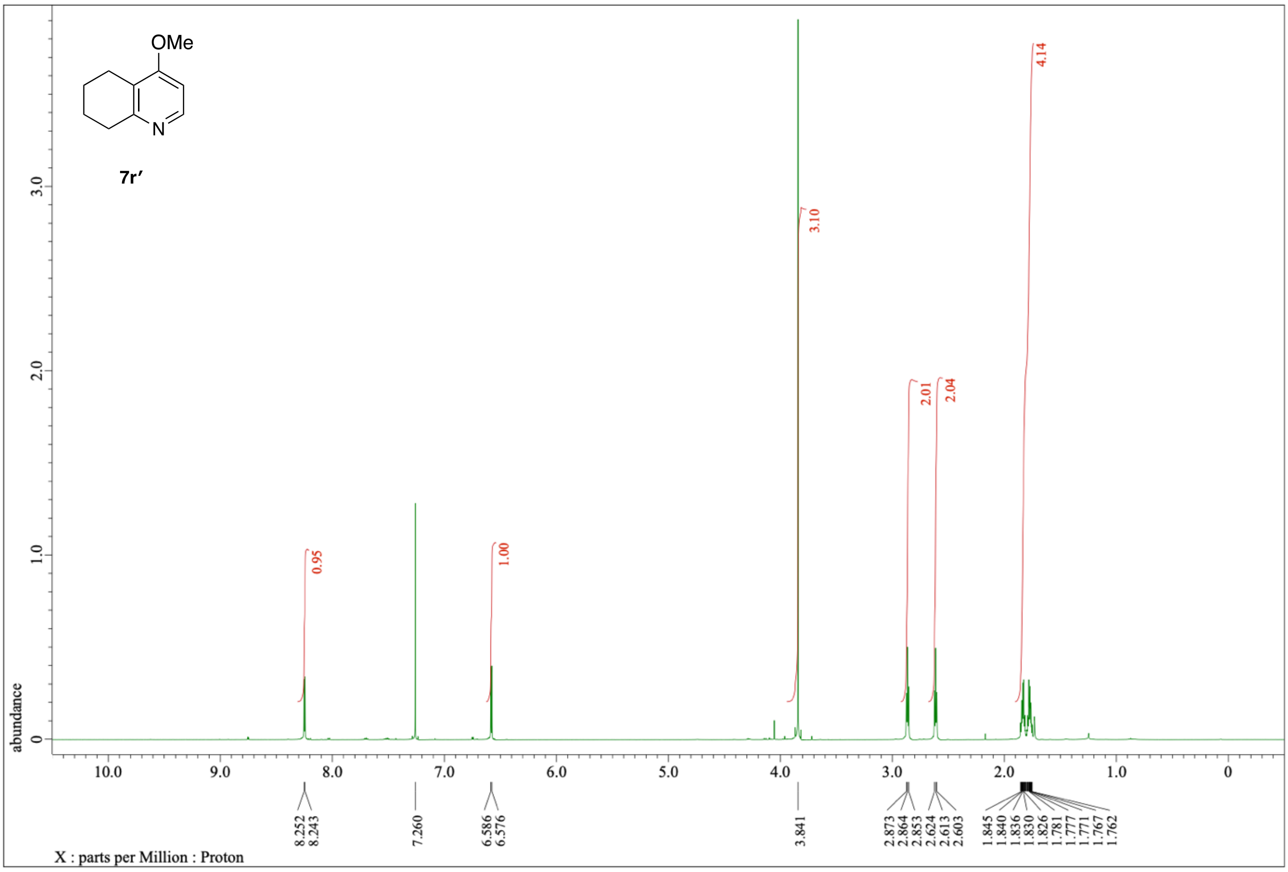

4-Methoxy-5,6,7,8-tetrahydroquinoline (7r′) $^{13}C\{^{1}H\}$ NMR (150 MHz, $CDCl_3$)

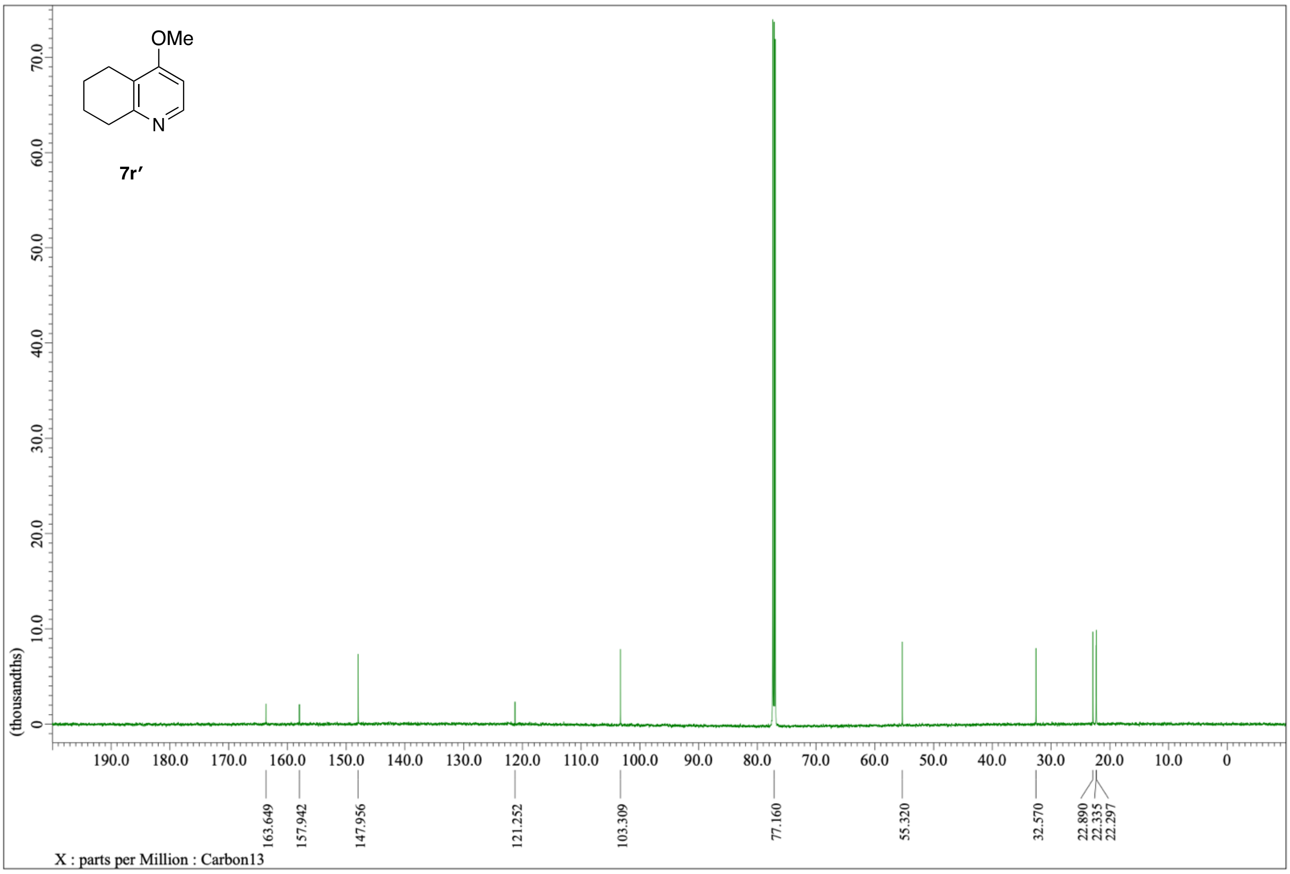

6-Fluoro-1,2,3,4-tetrahydroquinoline (7s) ^{1}H NMR (600 MHz, $CDCl_3$)

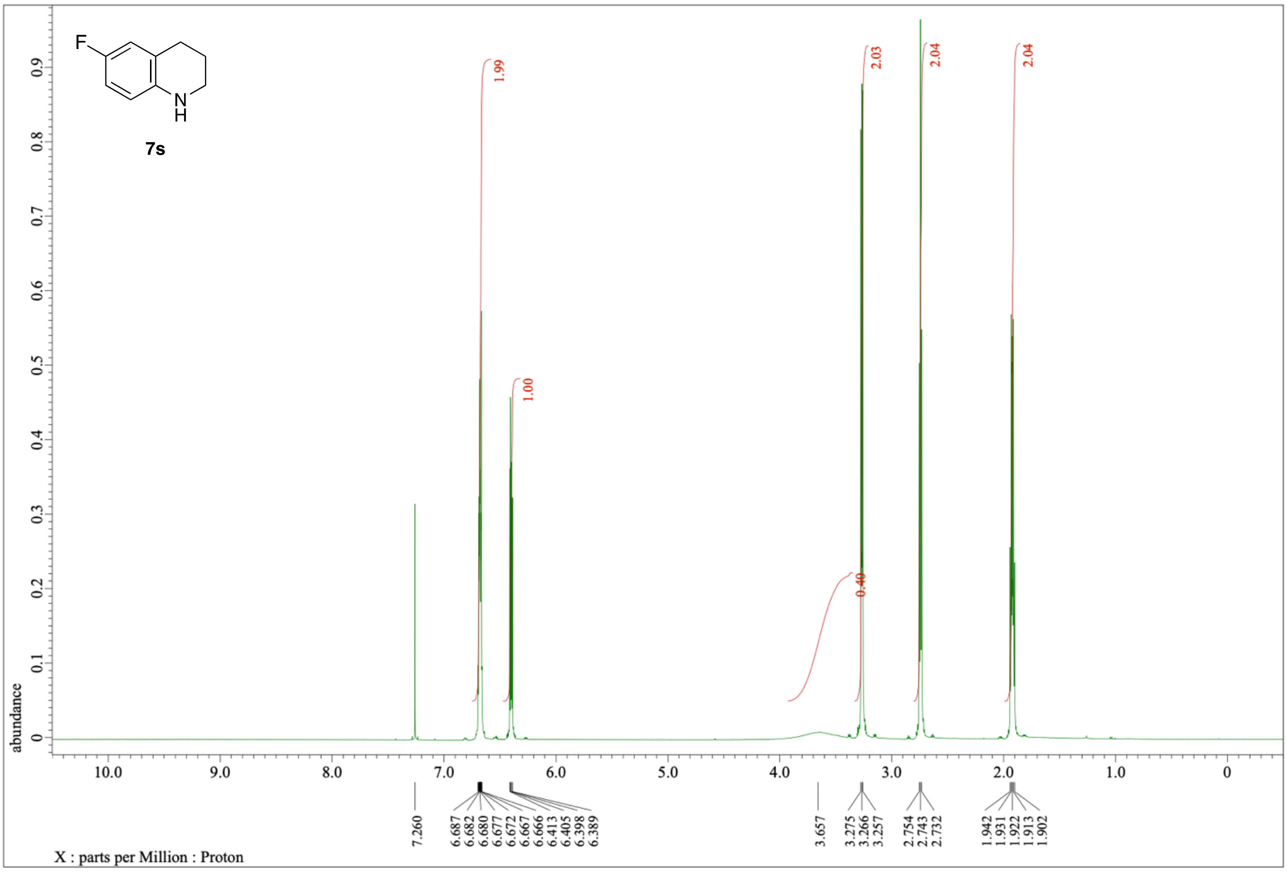

6-Fluoro-1,2,3,4-tetrahydroquinoline (7s) $^{13}C\{^1H\}$ NMR (150 MHz, $CDCl_3$)

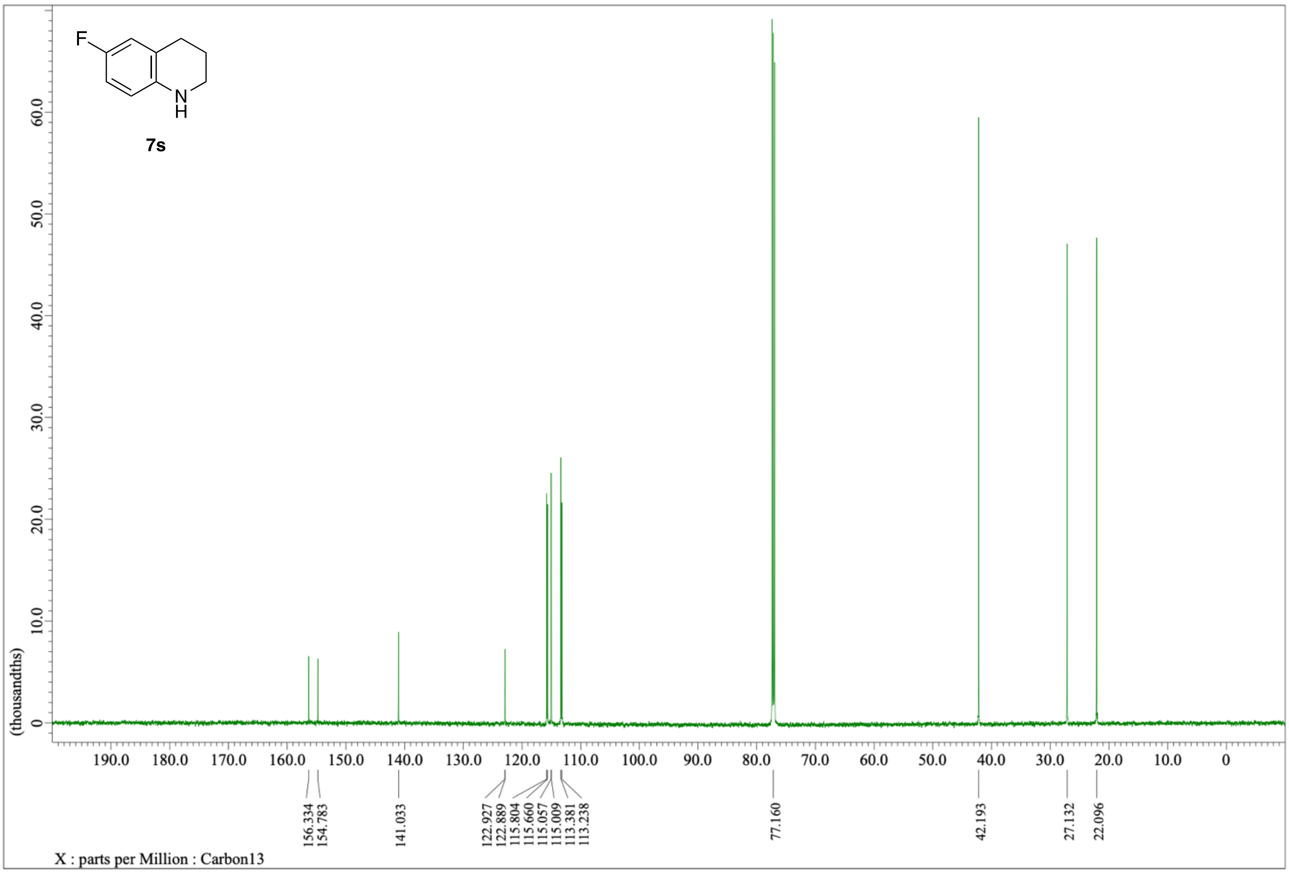

6-Fluoro-1,2,3,4-tetrahydroquinoline (7s) $^{19}F\{^1H\}$ NMR (565 MHz, $CDCl_3$)

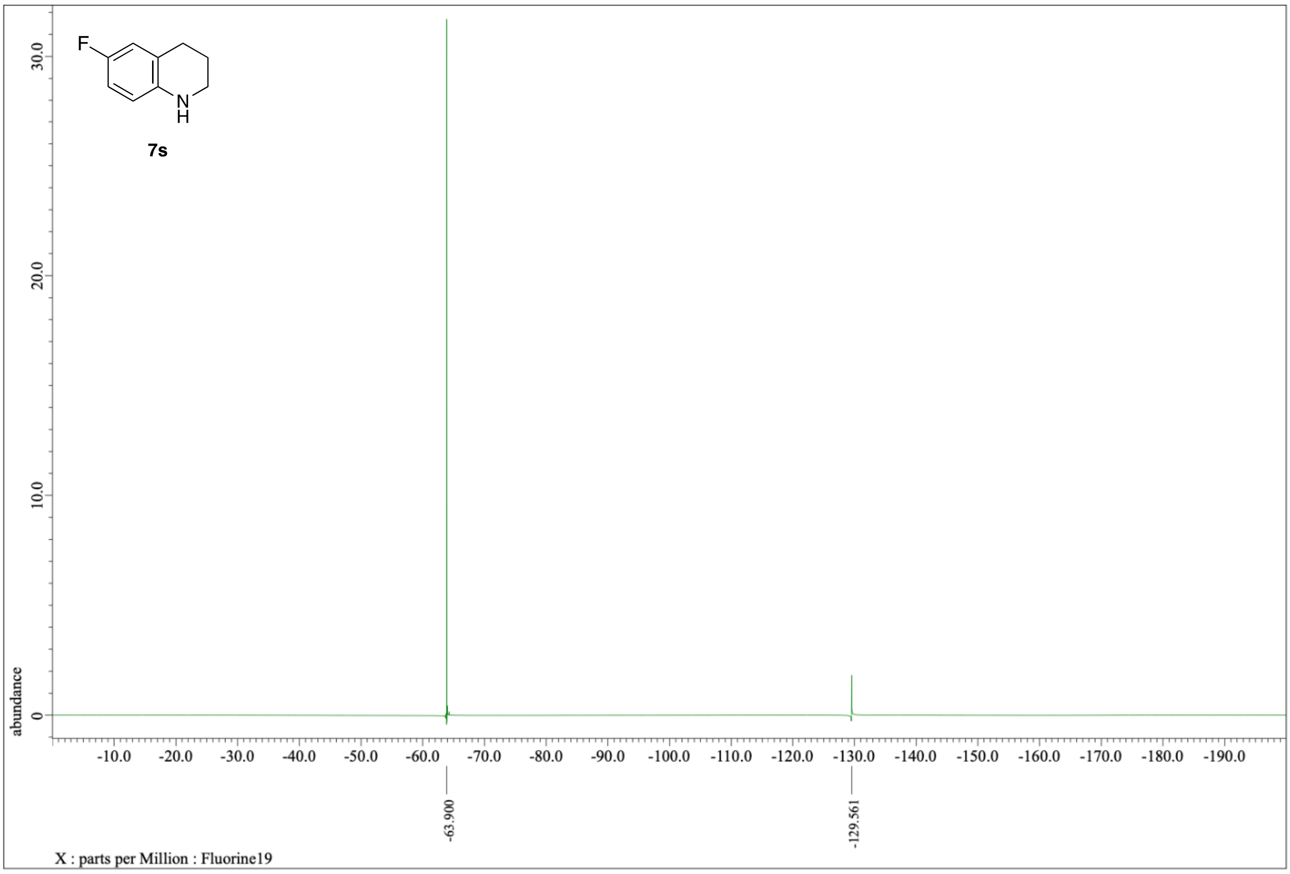

6-Chloro-1,2,3,4-tetrahydroquinoline (7t) ^{1}H NMR (600 MHz, $CDCl_3$)

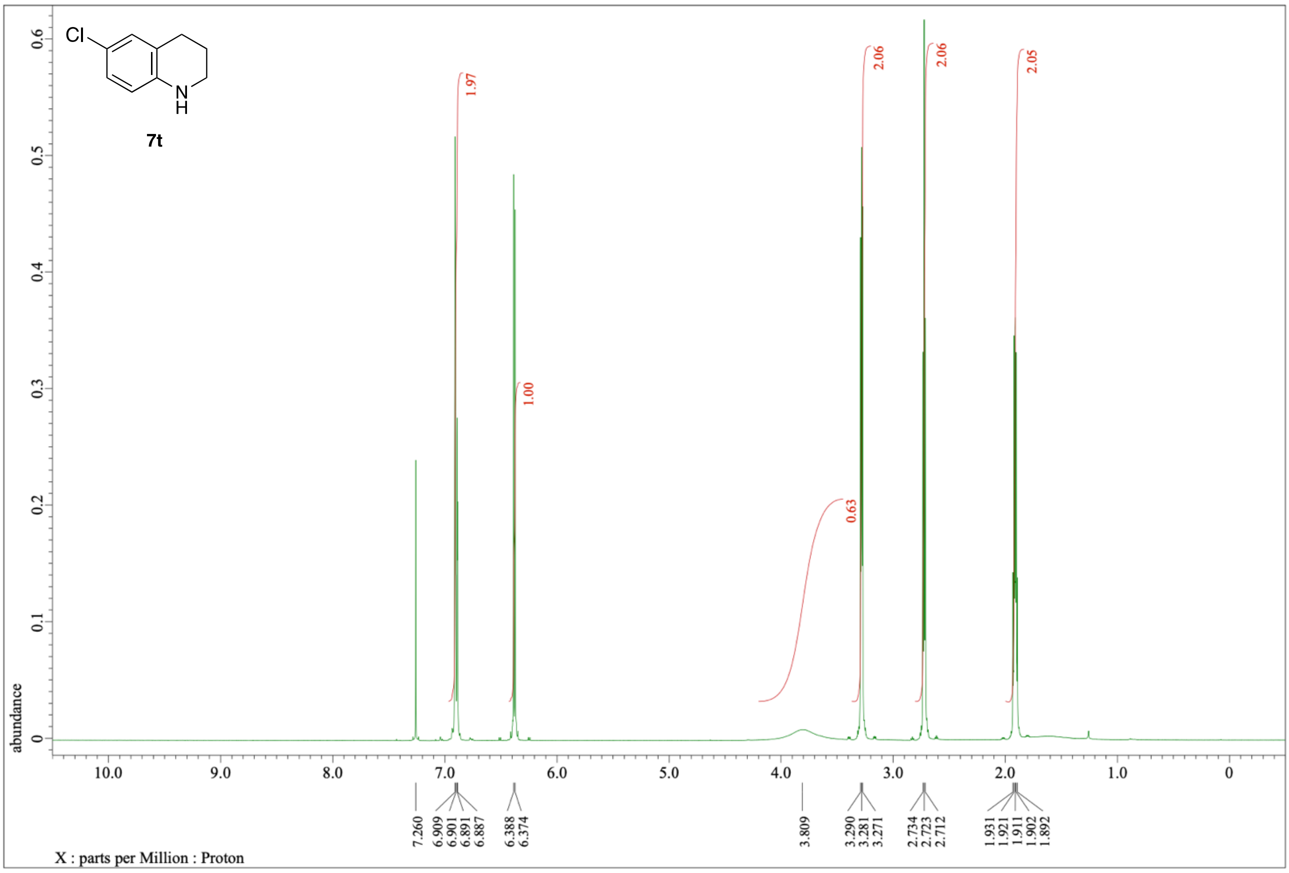

6-Chloro-1,2,3,4-tetrahydroquinoline (7t) ^{13}C{^{1}H} NMR (150 MHz, $CDCl_3$)

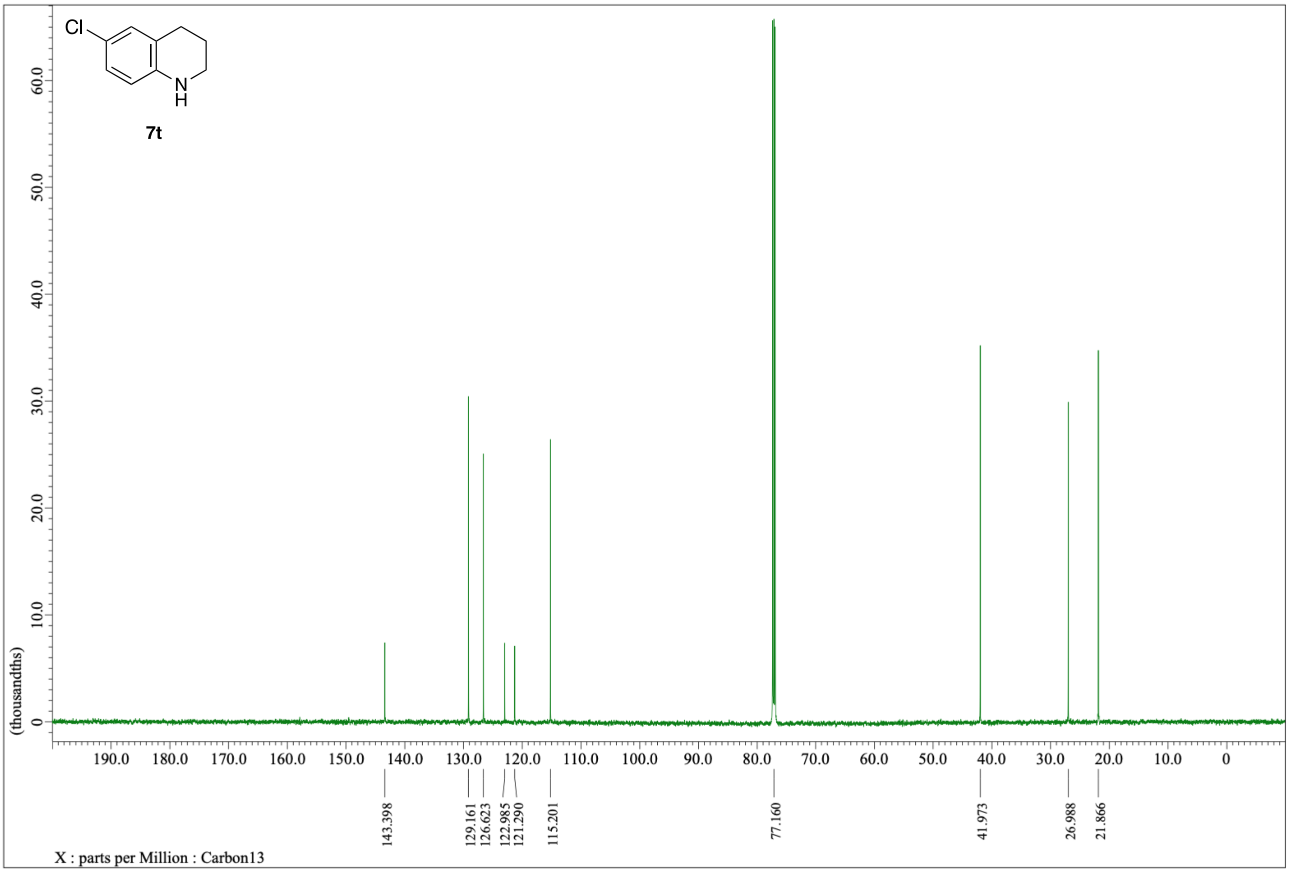

Supplement: File 1 — Experimental part. [file Beilstein_J_Org_Chem-20-1560-s001.pdf]
